# Novel Radioiodinated and Radiofluorinated Analogues of FT-2102 for SPECT or PET Imaging of mIDH1 Mutant Tumours

**DOI:** 10.3390/molecules27123766

**Published:** 2022-06-11

**Authors:** Valérie Weber, Lucie Arnaud, Sladjana Dukic-Stefanovic, Barbara Wenzel, Valérie Roux, Jean-Michel Chezal, Thu-Hang Lai, Rodrigo Teodoro, Klaus Kopka, Elisabeth Miot-Noirault, Winnie Deuther-Conrad, Aurélie Maisonial-Besset

**Affiliations:** 1 Université Clermont Auvergne, Inserm, Imagerie Moléculaire et Stratégies Théranostiques, UMR 1240, F-63000 Clermont-Ferrand, France; valerie.weber@uca.fr (V.W.); lucie.arnaud@uca.fr (L.A.); valerie.roux96@gmail.com (V.R.); j-michel.chezal@uca.fr (J.-M.C.); elisabeth.noirault@uca.fr (E.M.-N.); 2 Helmholtz-Zentrum Dresden-Rossendorf, Institute of Radiopharmaceutical Cancer Research, Research Site Leipzig, 04318 Leipzig, Germany; s.dukic-stefanovic@hzdr.de (S.D.-S.); b.wenzel@hzdr.de (B.W.); t.lai@hzdr.de (T.-H.L.); r.teodoro@life-mi.com (R.T.); k.kopka@hzdr.de (K.K.); 3 Department of Research and Development, ROTOP Pharmaka GmbH, 01328 Dresden, Germany; 4 Technical University Dresden, School of Science, Faculty of Chemistry and Food Chemistry, 01062 Dresden, Germany

**Keywords:** SPECT/PET imaging, mIDH1, FT-2102, radioiodination, radiofluorination

## Abstract

Isocitrate dehydrogenases (IDHs) are metabolic enzymes commonly mutated in human cancers (glioma, acute myeloid leukaemia, chondrosarcoma, and intrahepatic cholangiocarcinoma). These mutated variants of IDH (mIDH) acquire a neomorphic activity, namely, conversion of α-ketoglutarate to the oncometabolite D-2-hydroxyglutarate involved in tumourigenesis. Thus, mIDHs have emerged as highly promising therapeutic targets, and several mIDH specific inhibitors have been developed. However, the evaluation of mIDH status, currently performed by biopsy, is essential for patient stratification and thus treatment and follow-up. We report herein the development of new radioiodinated and radiofluorinated analogues of olutasidenib (FT-2102) as tools for noninvasive single photon emission computed tomography (SPECT) or positron emission tomography (PET) imaging of mIDH1 up- and dysregulation in tumours. Nonradiolabelled derivatives **2** and **3** halogenated at position 6 of the quinolinone scaffold were synthesised and tested in vitro for their inhibitory potencies and selectivities in comparison with the lead compound FT-2102. Using a common organotin precursor, (*S*)-[^125^I]**2** and (*S*)-[^18^F]**3** were efficiently synthesised by radio-iododemetallation and copper-mediated radiofluorination, respectively. Both radiotracers were stable at room temperature in saline or DPBS solution and at 37 °C in mouse serum, allowing future planning of their in vitro and in vivo evaluations in glioma and chondrosarcoma models.

## 1. Introduction

Isocitrate dehydrogenase enzymes (IDH) catalyse the NAD(P)-dependent oxidative decarboxylation of isocitrate to α-ketoglutarate (α-KG). At the cellular level, IDH function is of relevance to prevent oxidative stress, control the redox equilibria, and regulate α-KG-dependent processes [1,2]. Three isoforms of IDH have been described so far. In contrast to IDH1, a cytoplasmic and peroxisome enzyme, IDH2 and IDH3 are more particularly located in the mitochondria [3]. While somatic mutations of *IDH1* and *IDH2* have been reported for several cancers, *IDH3* mutations have rarely been detected in human tumours [4,5]. *IDH1* and *IDH2* are frequently mutated in low-grade gliomas (grade 2–3) and secondary glioblastomas (70 to 90% of patients with glial tumours harboured an *IDH* mutation, mainly mIDH1) [6,7], acute myeloid leukaemia (AML, 15 to 20% of patients presented *IDH* mutations, with equal repartition between both isoforms) [2,8], chondrosarcoma (CHS, 50% of patients with central and periosteal CHS presented *IDH* mutations, mostly in IDH1) [9,10], intrahepatic cholangiocarcinoma (10 to 20%), and other tumours with lower frequencies (e.g., prostate cancer or angioimmunoblastic T-cell lymphoma) [11]. These mutations induce a neomorphic activity, namely, conversion of α-KG to D-2-hydroxyglutarate (D2HG), which has been identified as an important oncometabolite involved in different signalling pathways, such as cell proliferation and apoptosis, but also in inducing DNA and histone methylation changes, thus promoting tumourigenesis [2]. Compared with wildtype (wt) *IDH1/2* tumours, 10- to 100-fold higher concentrations of D2HG were reported in *IDH*-mutated cancers [12,13]. In the last years, the therapeutic potential of several mutated IDH1/2 (mIDH1/2) inhibitors has been intensively investigated to counteract this neomorphic activity and reduce D2HG levels in tumours [14]. Two of these inhibitors, ivosidenib (AG-120, TIBSOVO^®^, Servier/Agios Pharm., Cambridge, MA, USA) and enasidenib (AG-221, IDHIFA^®^, Servier/Agios Pharm., Cambridge, MA, USA), which target variants mIDH1 and mIDH2, respectively, have been approved by the United States Food and Drug Administration (FDA) for the treatment of AML [15,16]. Recently, ivosidenib also received FDA approval for locally advanced or metastatic cholangiocarcinoma with mIDH1 mutations [17]. Further mIDH inhibitors, such as AG-881 (vorasidenib, Servier/Agios Pharm., Cambridge, MA, USA), IDH305 (Novartis, Basel, Switzerland), and olutasidenib (FT-2102, Forma Therapeutics, Watertown, MA, USA), are currently under investigation in several clinical trials [5,18,19]. In parallel to the clinical validation of new targeted therapies, drug approval agencies, such as the FDA, highlight the importance of developing companion diagnostics that can influence the treatment direction. They provide early information on the levels of expression of the biological targets in tumours as well as the potential therapeutic response of individual patients to such personalised medicine approaches. This can be performed, when possible, by gene sequencing, immunohistochemistry, or fluorescence in situ hybridisation analysis after biopsy sampling. However, biopsies do not always reflect the heterogeneity in tumours and might be troublesome for patients. In this context, whole-body noninvasive scintigraphic imaging techniques such as positron emission tomography (PET) and single photon emission computed tomography (SPECT) can be of valuable help to collect data regarding target expression levels during evaluation of potential drug candidates and to define the optimal dose for therapeutic applications. Diagnostic radiopharmaceuticals can also be used to assess therapy response, identify recurrences, and avoid repetitive biopsies during long term follow-up of patients.

**Figure 1:**
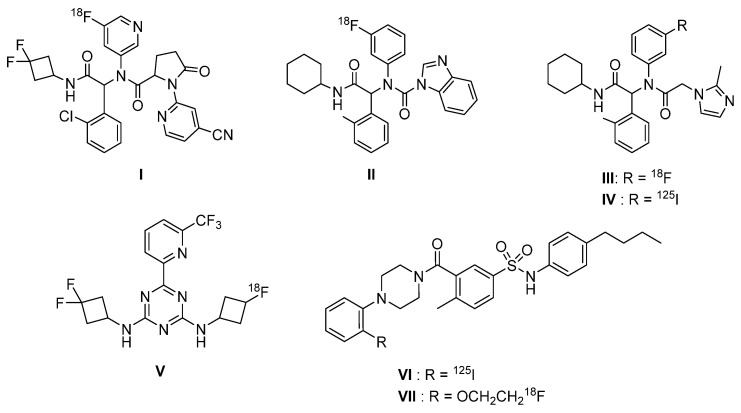
Structures of known mIDH1 targeting radiotracers.

To date, a few radiotracers for scintigraphic imaging of mIDH have been designed based on three chemical scaffolds, headed by the two clinically available drugs, ivosidenib (AG-120, phenyl-glycine) and enasidenib (AG-221, triazine), together with a butyl-phenyl sulfonamide class of mIDH1 inhibitors. However, none of these have been clinically validated so far [20,21,22,23]. Starting from the phenyl-glycine scaffold, compounds with high inhibitory potentials for mIDH1 (IC_50_ value <50 nM) were synthesised as mixtures of stereoisomers and radiolabelled with fluorine-18 or iodine radioisotopes (**I**, **II**, **III**, and **IV**, Figure 1) [21,23]. The preliminary results showed stability against dehalogenation in vivo, but several drawbacks, such as the lack of a diastereoselective (radio)synthesis, low radiochemical yields, and insufficient brain uptake, suggested a need for further developments regarding this class of compounds. The fluorine-18 radiolabelled triazine **V** showed specific uptake in mIDH1 glioma cell lines and in glioma xenografts, but these promising results were hampered by a significant defluorination of the radiotracer in vivo [20]. Butyl-phenyl sulfonamides **VI** and **VII**, radiolabelled with fluorine-18 or iodine-125, suffered from low inhibitory potency (IC_50_ values: 1.7 μM and 2.3 μM, respectively) as well as high nonspecific binding in vivo [22]. Altogether, the development of a radiotracer allowing the detection of IDH mutations in solid tumours by SPECT or PET modalities remains an important challenge.

**Figure 2:**
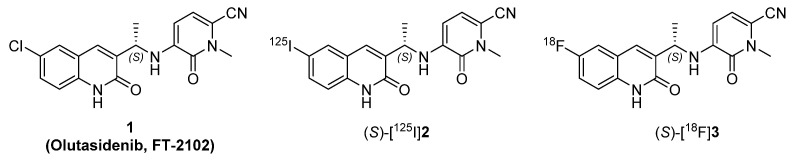
FT-2102 and its corresponding radioiodinated and radiofluorinated analogues.

With this aim, we report herein the development of radioiodinated ((*S*)-[^125^I]**2**) and radiofluorinated ((*S*)-[^18^F]**3**) mIDH1 specific tracers for SPECT and PET imaging of low-grade gliomas and CHS, chosen as central as well as peripheral solid tumours most frequently harbouring *IDH1* mutations (Figure 2).

These radiotracers were designed based on the chemical scaffold of FT-2102 (**1**), chosen for its nanomolar binding affinity towards several mutated forms of IDH1 together with its nonappreciable inhibition against wt IDH1 and IDH2 mutants, favourable blood–brain barrier (BBB) penetration, and low efflux ratio [24]. Among existing radiohalogens, fluorine-18, the most frequently used radionuclide for PET imaging, was selected for its favourable physical characteristics (half-life: 110 min, allowing multistep synthetic approaches and relatively long imaging protocols; 97% β^+^ decay; 635 keV positron energy) and production facilities (routinely produced at the multi-Curie level on widely implemented biomedical cyclotrons using the (p, n) nuclear reaction on an oxygen-18-enriched water target with a relatively low-energy proton beam). The multiple radioisotopes of iodine were also considered, as they offer a wide range of applications in nuclear medicine and provide a convenient bridge between preclinical research and clinical trials. Indeed, iodine-125 (half-life: 59.41 d; γ emitter) is suitable for preclinical in vitro experiments and small animal SPECT imaging, while iodine-123 (half-life: 13.22 h; γ emitter) and iodine-124 (half-life: 4.17 d; β^+^ emitter) are well suited for clinical SPECT and PET imaging purposes, respectively. In addition, iodine-131 (half-life: 8.03 d, β^—^emitter) can be used for targeted radionuclide therapeutic approaches.

Structure–activity relationship studies have provided an extensive elucidation of the interaction mode of FT-2102 with the mIDH1 R132H enzyme [24,25,26]. Accordingly, positions 6 and 7 of the FT-2102 quinolinone core, oriented towards a hydrophobic pocket, can be modified with the lower risk of impact on the biological target affinity. Consequently, we decided to synthesise FT-2102 analogues (*S*)-**2** and (*S*)-**3** with iodine and fluorine atoms grafted on position 6 of the quinolinone ring in replacement of the chlorine atom (Figure 2). The corresponding enantiomers (*R*)-**2** and (*R*)-**3** were also synthesised in order to determine the enantiomeric excess (*e.e.*) of the final compounds.

## 2. Results and Discussion

### 2.1. Chemistry

**Scheme 1:**
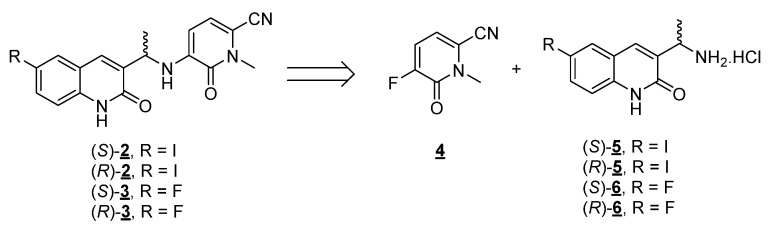
Retrosynthetic approach for the production of iodinated and fluorinated derivatives (*S*)-**2** and (*S*)-**3** and their enantiomers.

Designed final compounds **2** and **3** were obtained by coupling halogenoquinolinones **5** and **6**, each bearing a 1-aminoethyl group at position 3, with the *N*-methyl fluoropyridinone **4** (Scheme 1).

**Scheme 2:**
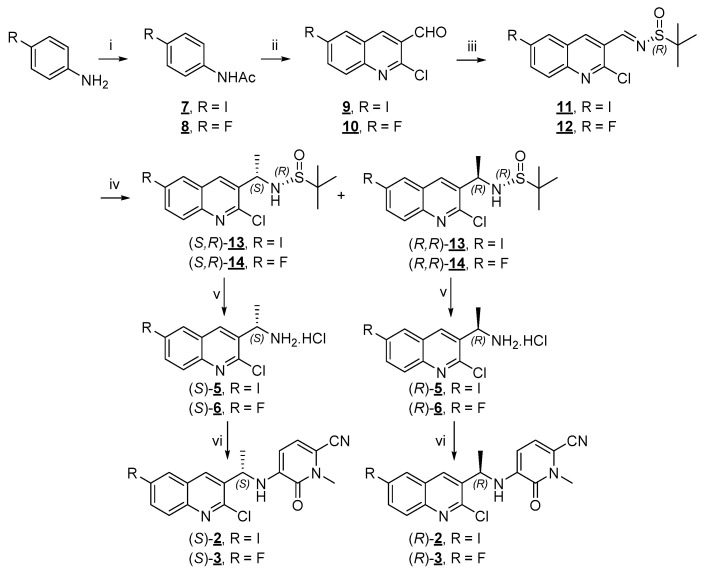
Synthesis of iodinated and fluorinated derivatives (*S*)-**2**, (*S*)-**3** and their enantiomers. Reagents and conditions: (**i**) Ac_2_O, DIPEA, DCM, rt, 1–2.5 h; (**ii**) (a) POCl_3_, anh. DMF, 0 °C, then rt for 15–20 min; (b) **7** or **8**, 80–85 °C, 16–19 h; (**iii**) ^®^-2-methylpropane-2-sulfinamide, DCE, CuSO_4_, 55 °C, 17 h—3 d; (**iv**) MeMgBr 3 M in Et_2_O, anh. DCM, −60 °C for 3 h, then −60 °C to rt overnight; (**v**) 1 N HCl aq., 1,4-dioxane, 105–110 °C, overnight; (**vi**) **4**, DIPEA, DMSO, 110 °C, 15–22 h.

Compound **4** was obtained in four synthesis steps starting from the commercial 5-fluoropicolinonitrile, according to the procedure described by Caravella et al. [24] (see Supplementary Materials) with an average overall yield of 16%. Intermediates **5** and **6** were produced, starting from 4-iodo or 4-fluoroaniline, via a five-step synthetic approach adapted from those developed for chlorinated derivatives and described by Lin et al. [25] and Ashwell et al. [27] (Scheme 2).

Commercial 4-iodoaniline and 4-fluoroaniline were first acetylated using acetic anhydride in the presence of *N,N*-diisopropylethylamine (DIPEA) to produce acetamides **7** and **8** in quantitative yields. Then, the 2-chloro-3-formylquinoline scaffold of derivatives **9** and **10** was obtained according to Meth-Cohn et al. [28] by reaction of acetamides **7** and **8** with the Vilsmeier–Haack reagent, generated in situ by treatment of *N,N*-dimethylformamide (DMF) with phosphorus oxychloride. Using standard reaction conditions usually described in the literature [25,27], compounds **9** and **10** were isolated with low yields, 10–18% (n = 3) and 5–13% (n = 3), respectively. This result can be explained by the competitive and irreversible formation of the corresponding *N*’-(4-halogenophenyl)-*N*,*N*-dimethylformamidine by-product as previously observed for similar *para*-substituted acetanilides (e.g., those bearing halogen atoms or methoxy group) [29]. Consequently, several attempts were performed, mainly on the iodinated derivative **7**, to optimise the reaction yield. Unfortunately, no desired product could be isolated by increasing the temperature or the equivalents of DMF and/or phosphorus oxychloride. Furthermore, activation of the reaction by sonication at lower temperature or by using acetonitrile (ACN) and/or sodium dodecyl sulfate (SDS) as additives according to the protocols of Ali et al. remained unsuccessful [30,31].

After this critical step, a condensation of the aldehyde function of derivatives **9** and **10** with the commercial chiral auxiliary (*R*)-2-methylpropane-2-sulfinamide in the presence of copper sulfate produced the imine compounds **11** and **12** with moderate to high yields (67–87%). Then, the reaction with methyl magnesium bromide at low temperature (−60 °C) allowed creating asymmetric carbon and efficiently produced (*S*,*R*)-**13** and (*S*,*R*)-**14** with yields between 62 and 66%. Interestingly, for each reaction, small quantities of the corresponding diastereomers (*R*,*R*)-**13** and (*R*,*R*)-**14** (10–15% of the *R*,*R* diastereomers determined by ^1^H NMR analysis of the crude mixture) were also isolated in 15% and 11% yields, respectively, after separation by column chromatography. Advantageously, both diastereomers were engaged in the following synthesis steps in order to obtain the enantiomers of each final desired compounds **2** and **3** and facilitate the *e.e.* determination. Accordingly, the acidic cleavage of the chiral auxiliary moiety and concomitant hydrolysis of the 2-chloroquinoline group produced quinolinones (*S*)- and (*R*)-**5**, and (*S*)- and (*R*)-**6** as hydrochloride salts, which were directly coupled with the *N*-methylpyridinone **4**. Thus, the enantiomers (*S*)- and (*R*)-**2** and (*S*)- and (*R*)-**3** were successfully synthesised in around 40% yields over two steps.

**Figure 3:**
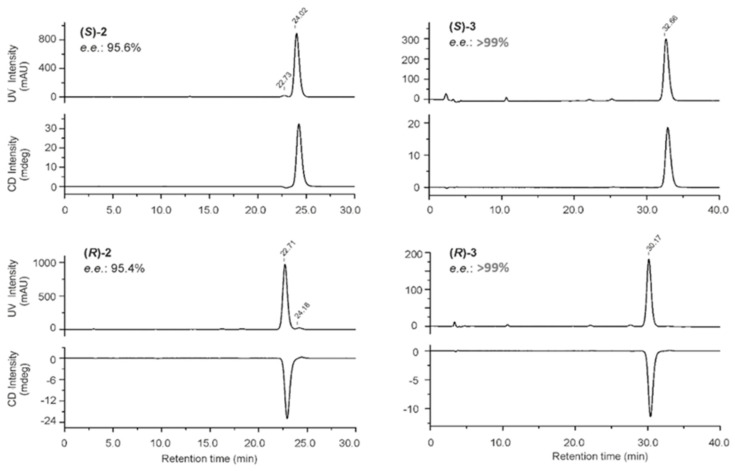
Chromatograms of the chiral HPLC separation of (*S*)- and (*R*)-**2** and (*S*)- and (*R*)-**3**. Conditions: (a) Column: CHIRALPAK IB, (b) Mobile phase composition: ACN/aq. 20 mM NH_4_OAc (46/54, *v*/*v* for (*S*)- and (*R*)-**2** and 34/66, *v*/*v* for (*S*)- and (*R*)-**3**), (c) CD detection at 285 nm.

The *e.e.* of the final compounds were determined by chiral HPLC using UV detection in combination with a circular dichroism (CD) detector. Under these conditions, *e.e.* values were achieved of >95% for the iodinated (*S*)- and (*R*)-**2** enantiomers and up to >99% for the fluorinated (*S*)- and (*R*)-**3** analogues (Figure 3). These results confirmed the stereoselectivity of the methylation step as well as the efficiency of the separation of the corresponding diastereomeric intermediates (*S*,*R*)-**13**/(*R*,*R*)-**13** and (*S*,*R*)-**14**/(*R*,*R*)-**14**.

**Figure 4:**
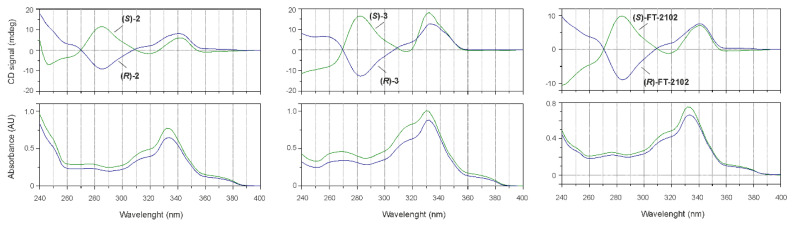
CD and UV spectra of (*S*)- and (*R*)-**2**, (*S*)- and (*R*)-**3,** and (*S*)- and (*R*)-FT-2102 measured with a chiral HPLC in stopped-flow mode (different compositions of ACN/aq. 20 mM NH_4_OAc as mobile phases).

Circular dichroism is the ability of optically active compounds to differently absorb right and left circularly polarised light. This difference is recorded, and the value is referred to as ellipticity, often given in millidegrees (mdeg). As the ellipticity value depends on the absorbance wavelength and thus on the chromophores of a compound, the resulting CD spectrum is a typical characteristic for a chiral compound, and the two enantiomers give mirror-image spectra. Usually, the UV absorbance is recorded simultaneously with the CD spectrum, which can be used to determine further parameters. The selection of the most suitable wavelength for CD monitoring of the new derivatives (*S*)- and (*R*)-**2** and (*S*)- and (*R*)-**3** was the first procedure to be performed, and the corresponding CD spectra were recorded with chiral HPLC in stopped-flow mode. As shown in Figure 4, the compounds had strong CD bands in the ranges of 280–290 and 330–350 nm. At 285 nm, a positive CD band was related to the *S*-configuration, and a negative CD band to the *R*-configuration, of the compounds and was therefore chosen as optimal monitoring wavelength. Interestingly, in the range of 330–350 nm, the CD bands were positive for both enantiomers. We therefore investigated the commercially available enantiomers of FT-2102 and observed the same phenomenon (Figure 4). This CD band was related to the *N*-methylpyridinone chromophore, as illustrated with the CD and UV spectra of the corresponding reactant **4** (Figure S1). The reliable functionality of the CD detector was also verified by scanning (*S*)- and (*R*)-binaphthol as suitable reference enantiomers absorbing in the considered wavelength region (Figure S2).

### 2.2. Biological Evaluation

The synthesised FT-2102 analogues (*S*)-**2** and (*S*)-**3** with iodine or fluorine atoms on position 6 of the quinolinone ring and their enantiomers (*R*)-**2** and (*R*)-**3** were evaluated for their inhibitory potential against different mutated enzymes. The main mutation of the IDH1 enzyme is often due to a missense substitution predominantly affecting the arginine 132 in the substrate-binding site of IDH1 [6,11]. IDH1 R132H is present in 65% of glioma tumours, and IDH1 R132C is the most frequent IDH mutation encountered in CHS (40%) [11]. The selectivity of both compounds towards wt IDH1 and IDH2 was also investigated (Table 1).

With replacement of chlorine with iodine, compound (*S*)-**2** maintained similar potency for all tested IDHs as that of the lead compound FT-2102 (5.23 ± 0.75 nM vs. 4.88 ± 1.63 nM, respectively, on IDH1 R132H and 160 ± 24 nM vs. 178 ± 70 nM, respectively, on IDH1 R132C). The introduction of a fluorine atom in place of chlorine also resulted in good retention of inhibitory potency (22.7 ± 5.2 nM) despite an approximate fourfold decrease when compared with iodinated compound (*S*)-**2**. As the 6-chloro substituent of the quinolinone ring of FT-2102 is positioned in a hydrophobic pocket [24,26], the better inhibitory potency of *(S)*-**2** compared with *(S)*-**3** could be explained by the ability of iodine, due to its lower electronegativity, to form stronger hydrophobic interactions with the neighbouring nonpolar amino acids in the active site. As expected, the *(R)-*methyl enantiomers were substantially less potent, with at least 30-fold lower inhibition of the two mutants (IC_50_: 323 nM and 698 nM on IDH1 R132H and >10,000 nM on IDH1 R132C). Indeed, the *(S)*-methyl substituted benzylic linker was required to adopt the bioactive conformation, which provided reduced entropic cost of ligand binding and potent inhibitory activity against IDH1 mutants [24]. For the inhibition of wt IDH1/2, a concentration in the micromolar range was required for all tested compounds, proving a high selectivity of derivatives (*S*)-**2** and (*S*)-**3** for the mutated enzymes. Hence, based on their promising inhibitory potential and selectivity profiles, both (*S*)-compounds were selected for further radiolabelling and stability studies.

### 2.3. Radiochemistry

**Scheme 3:**
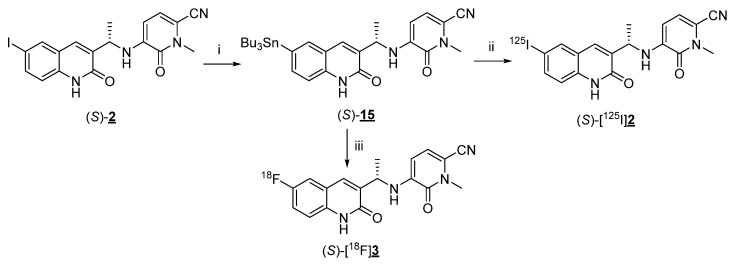
Synthesis of the organotin precursor (*S*)-**15** and corresponding radioiodinated (*S*)-[^125^I]**2** and radiofluorinated (*S*)-[^18^F]**3** tracers. Reagents and conditions: (**i**) Sn_2_Bu_6_, Pd_2_dba_3_, DIPEA, propan-2-ol, rt, 23 h; (**ii**) [^125^I]NaI, MeOH, CAT.3H_2_O, AcOH, rt, 5 min; (**iii**) [^18^F]LiF, Cu(OTf)_2_(py)_4_, DMA, 105–110 °C, 10 min.

To produce the radioiodinated and radiofluorinated tracers, a common organotin precursor (*S*)-**15** was chosen (Scheme 3). Starting from this precursor, the radioiodinated (*S*)-[^125^I]**2** tracer and the radiofluorinated compound (*S*)-[^18^F]**3** were produced by conventional electrophilic radioiododestannylation under mild conditions and copper-mediated nucleophilic aromatic radiofluorination with [^18^F]F^−^, respectively. Classically, organotin derivatives can be synthesised from the corresponding iodinated analogues via a palladium-catalysed I/SnBu_3_ exchange reaction. Accordingly, the key organotin precursor (*S*)-**15** was first produced by reaction of the iodinated derivative (*S*)-**2** with hexabutylditin in the presence of tetrakis(triphenylphosphine)palladium(0) (Pd(PPh_3_)_4_) in refluxing toluene or 1,4-dioxane. Unfortunately, in such conventional conditions, the precursor (*S*)-**15** was obtained only in low yields (21–25%) with a concomitant degradation of both iodinated reactant and organotin product in the reaction media (visualised during the TLC monitoring of the reaction). To circumvent this problem, a milder Pd-catalysed stannylation reaction [32], developed for sensitive multifunctional compounds and compatible with bioactive molecules, was investigated. Accordingly, (*S*)-**2** was treated with hexabutylditin in the presence of tris(dibenzylideneacetone)dipalladium(0) (Pd_2_dba_3_) and DIPEA in propano-2-ol at room temperature for one day. Under these conditions, the desired organotin precursor (*S*)-**15** was successfully isolated in 54% yield with significantly reduced side-product formation.

**Figure 5:**
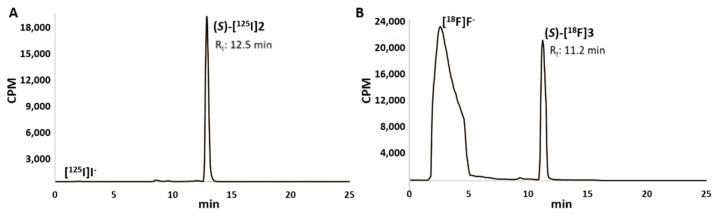
Radioactivity-HPLC chromatograms of the crude reaction mixture of the radioiodination (**A**) and radiofluorination (**B**) from precursor (*S*)-**15**.

Then, we turned our attention to the production of the radioiodinated tracer (*S*)-[^125^I]**2**. The radiolabelling of the organotin precursor (*S*)-**15** with no-carrier-added [^125^I]NaI was performed at high molar activity in the presence of chloramine-T (CAT) trihydrate as a mild oxidative agent under acidic conditions. After 5 min at room temperature, high radiochemical conversion (RCC, 97.7 ± 2.6%, n = 7) was observed (Figure 5A).

**Figure 6:**
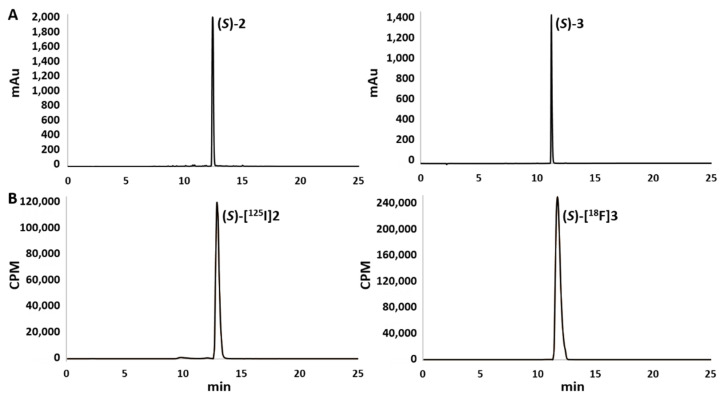
(**A**): UV-HPLC chromatograms (330 nm) of iodinated and fluorinated reference compounds (*S*)-**2** and (*S*)-**3**; (**B**): radioactivity-HPLC chromatograms of the corresponding radiotracers (*S*)-[^125^I]**2** and (*S*)-[^18^F]**3** obtained after semipreparative RP-HPLC purification and formulation. The radioactivity detector was connected in series after the UV detector, which accounts for the slight difference of retention times (≈ 0.3 min) observed between ^125^I/^127^I or ^18^F/^19^F products.

After purification by semipreparative RP-HPLC and C18 solid-phase extraction (SPE), subsequent formulation in a sterile saline solution (ethanol content ca. 5%) provided (*S*)-[^125^I]**2** with good radiochemical yields (RCY, 85 ± 10%, n = 6), high radiochemical purities (RCP, 97.9 ± 1.0%, n = 6, Figure 6), and high molar activities (34.7 ± 8.6 GBq/µmol, n = 3).

**Figure 7:**
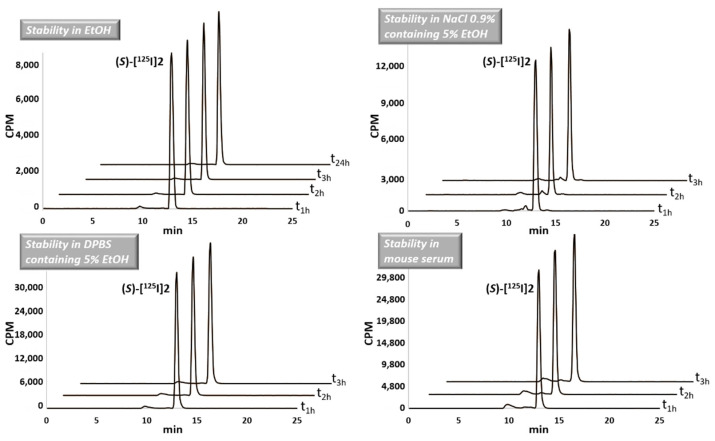
Radioactivity-HPLC chromatograms of the radiotracer (*S*)-[^125^I]**2** incubated, for 1, 2, and 3 h post formulation, at room temperature in ethanol, NaCl 0.9% containing 5% ethanol, and DPBS (pH 7.4) containing 5% ethanol, as well as in a mixture of DPBS and mouse serum at 37 °C.

In the ethanolic solution eluted after the C18 SPE cartridge, as well as in the formulation medium and in Dulbecco’s Phosphate Buffered Saline (DPBS, pH 7.4), the radiotracer was found to be stable over hours at room temperature (RCP: 98.0%, 97.2%, and 97.4%, respectively, at 3 h post formulation, Figure 7). In addition, no significant degradation or deiodination of the radiotracer (*S*)-[^125^I]**2** was detected in mouse serum after incubation for 3 h at 37 °C (RCP: 94.3%).

For the production of the radiofluorinated analogue (*S*)-[^18^F]**3** (Scheme 3), the same organotin precursor (*S*)-**15** was treated with [^18^F]LiF and tetrakis(pyridine)copper(II) triflate (Cu(OTf)_2_(py)_4_) in *N*,*N*-dimethylacetamide (DMA) according to a protocol routinely used in our laboratory for the copper-mediated radiofluorination of nonactivated aromatic derivatives in the presence of oxygen and with small amounts of base [33]. In all reaction conditions tested (variation of temperature, equivalents of Cu(OTf)_2_(py)_4_, molar quantity of precursor, duration of heating, etc.), moderate RCC was achieved (18.6 ± 2.5%, n = 7, Figure 5B). In parallel to the formation of (*S*)-[^18^F]**3**, no radioactive by-products except remaining [^18^F]F^−^ could be identified by radio-TLC monitoring or by analytical HPLC. Interestingly, a fast change in the colour of the reaction mixture (blue to dark brown/black) was noticed immediately after addition of the organotin precursor to the mixture [^18^F]LiF/Cu(OTf)_2_(py)_4_, even before any heating process. As efficiency of copper-mediated radiofluorinations can be affected by various parameters, such as the presence of chelating agents, a detrimental interaction of the *N*-heterocyclic precursor with the copper complex was suspected. In order to confirm this assumption, comparative assays were performed based on the work of Chen and coworkers [34]. A model radiofluorination reaction, namely the production of 7-[^18^F]fluorotryptophan (7-[^18^F]FTrP) starting from the corresponding aryl boronate precursor, was chosen [35]. With the optimised reaction conditions typically used in our laboratory, reproducible high RCC (88.1 ± 3.2%, n = 5, data not shown) was achieved for the production of 7-[^18^F]FTrP via copper-mediated radiofluorination. When adding an equimolar amount of the organotin precursor (*S*)-**15** in the reaction mixture of the radiofluorination of 7-[^18^F]FTrP, the labelling efficiency dramatically decreased to 19%, indicating that precursor (*S*)-**15** appeared to act as a poison of the copper complex. Nevertheless, (*S*)-[^18^F]**3** could be produced in sufficient amounts to perform subsequent purification steps. First, the crude radiofluorination mixture was purified by C18 SPE cartridge. The acetonitrile eluate obtained was then applied on a semipreparative HPLC system, and the collected fraction corresponding to (*S*)-[^18^F]**3** was passed through a second C18 SPE cartridge to remove HPLC solvents. Finally, elution with a small volume of ethanol and formulation provided the radiotracer (*S*)-[^18^F]**3** in a sterile saline solution (ethanol content ca. 5%) with moderate RCY (7.4 ± 2.4% decay corrected, n = 5), high RCP (99.8 ± 0.2%, n = 5, Figure 6), and molar activities of 3.56 ± 0.4 GBq/µmol (n = 3).

**Figure 8:**
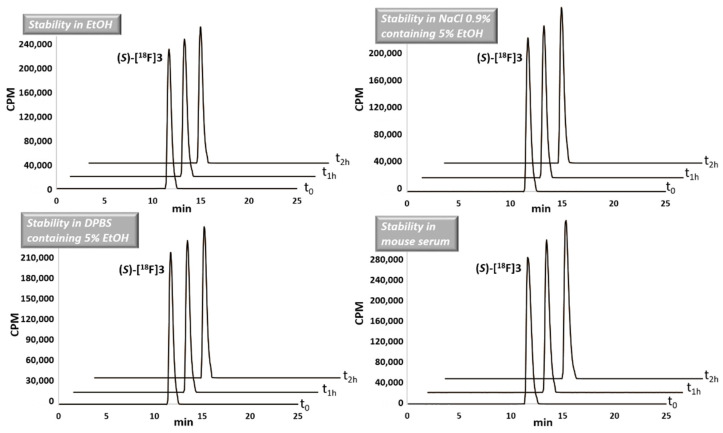
Radioactivity-HPLC chromatograms of the radiotracer (*S*)-[^18^F]**3** incubated, for 0, 1, and 2 h post formulation, at room temperature in ethanol, NaCl 0.9% containing 5% ethanol, and DPBS (pH 7.4) containing 5% ethanol, as well as in a mixture of DPBS and mouse serum at 37 °C.

Similarly to (*S*)-[^125^I]**2**, (*S*)-[^18^F]**3** was stable over 2 h in the ethanolic solution obtained from the final C18 SPE cartridge in the formulation media (NaCl 0.9% or DPBS (pH 7.4) containing 5% ethanol) and during incubation at 37 °C in mouse serum (RCP > 99%, Figure 8).

## 3. Materials and Methods

### 3.1. Chemistry

#### General Information

All commercially available reagents and solvents were purchased at the following commercial suppliers: Sigma Aldrich, Alpha Aesar, ABX, Acros Organics, Fisher Scientific or Carlo Erba Reagents, ABCR, and Bachem. All solvents were dried using common techniques [36]. Unless otherwise noted, moisture-sensitive reactions were conducted under dry argon atmosphere. Temperatures indicated in the protocols correspond to the temperature of the oil bath. Analytical thin layer chromatography (TLC) was performed on precoated silica gel 60 F254 or neutral aluminium oxide 60 F254 plates (Merck or Macherey-Nagel) and visualised with UV light (254 nm) and/or developed with iodine. Flash column chromatography was performed on silica gel 60A normal phase, 35–70 µm (Merck or SDS), or neutral aluminium oxide 90 standardised, 63–200 µm (Merck, column chromatographic adsorption analysis according to Brockmann). Preparative RP-HPLC purification was carried out on a CombiFlash EZ prep system (Teledyne Isco) equipped with a UV–visible detector (360 and 254 nm). Separation was performed on a C18 column (Teledyne, Redisep Prep C18, 15.5 g, 100 Å pore size, 20–40 µm) at room temperature. Uncorrected melting points (mps) were recorded on an electrothermal capillary Digital Melting Point Apparatus IA9300 (Bibby Scientific). Nuclear magnetic resonance (NMR) spectra (500 MHz for ^1^H and 126 MHz for ^13^C) were recorded on a Bruker Avance 500 instrument with chemical shift values (δ) expressed in parts per million (ppm) relative to residual solvent as standard, and coupling constants (*J*) are given in Hz. ^19^F NMR spectra (470.3 MHz) were recorded on the same Bruker Avance 500 apparatus using trifluorotoluene as internal reference (−63.72 ppm). Infrared spectra (IR) were recorded in the range of 4000–440 cm^−1^ on a Nicolet IS10 (Fisher Scientific) with an attenuated total reflectance (ATR) accessory. Compounds were analysed by high-resolution mass spectrometry (HRMS) in positive mode (Waters^®^ Micromass^®^ Q-Tof micro™ Mass Spectrometer). The chemical purity of the final compounds was determined by analytical RP-HPLC (HP1100 system, Hewlett Packard, Les Ulis, France) on a C-18 column (Agilent Zorbax Extend C18, 5 μm, 4.6 × 150 mm, equipped with a precolumn) using the following solvent conditions: water containing 0.1% of trifluoroacetic acid (solvent A) and ACN containing 0.1% of trifluoroacetic acid (solvent B); 0 to 3 min: isocratic elution 95% A; 3 to 15 min: gradient elution 95% → 5% A; 15 to 25 min: isocratic elution 5% A; 25 to 30 min: gradient elution 5% → 95% A with a flow rate of 1 mL/min, λ = 254 and 360 nm. Analytical chiral chromatographic measurements were performed on a JASCO LC-4000 system incorporating a PU-4180-LPG pump, an AS-4050 auto injector (100 µL sample loop), and a UV diode array detector MD-4015 (monitoring at 254 nm) coupled with a circular dichroism chiral detector CD-4095 (monitoring at 285 nm). Data analysis was performed with the ChromNAV 2.3C software (JASCO Deutschland GmbH, Pfungstadt, Germany). The CD spectra were recorded with the CD-4095 detector during the HPLC run in stopped-flow mode, during which which the flow through the detector cell was bypassed using a software-controlled switching valve. Each spectrum was obtained with a scanning speed of 10 nm/sec. The single enantiomers were dissolved in ACN/water, 2/1 (*v*/*v*) and subjected to chiral separations, which were performed on a CHIRALPAK IB column (250 × 4.6 mm, 5 µm, Daicel—Chiral Technologies Europe, France) in isocratic mode with ACN/ aq. 20 mM NH_4_OAc as mobile phase and a flow of 1 mL/min (data in Table S1). The *e.e*. was determined according to E.L. Eliel [37]. The ammonium acetate concentration, stated as aq. 20 mM NH_4_OAc, corresponds to the concentration in the aqueous component of the eluent mixture. (*S*)-FT-2102 and (*R*)-FT-2102 were purchased from Hölzel Diagnostica Handels GmbH (Köln, Germany) and Advanced ChemBlocks Inc. (Hayward, CA, USA), respectively.

***N*-(4-iodophenyl)acetamide (7).** To a solution of 4-iodoaniline (1.00 g, 4.57 mmol) and DIPEA (1.6 mL, 9.19 mmol) in DCM (10 mL) was added acetic anhydride (0.5 mL, 5.49 mmol) dropwise at 0 °C. The reaction mixture was stirred at room temperature for 2.5 h. Then, the reaction was quenched with deionised water (20 mL). After decantation, the aqueous layer was extracted with DCM (6 × 10 mL). The combined organic layers were washed with deionised water (20 mL), dried on MgSO_4_, filtered, and concentrated under vacuum. The residue was purified by column chromatography (Al_2_O_3_, cyclohexane/EtOAc, 6/4, *v*/*v*) to provide derivative **7** (1.17 g, 4.48 mmol, 98%) as a white powder. R_f_ (Al_2_O_3_, cyclohexane/EtOAc, 6/4, *v*/*v*) 0.47. IR (ATR, cm^−1^) 3285, 3251, 3043, 1662, 1596, 1579, 1526, 1482, 1388, 814. Mp 184 ± 1 °C (Lit. mp 183 °C [38]). ^1^H NMR (500 MHz, CDCl_3_) δ 7.61 (d, 2H, *^3^J* = 8.8 Hz, H-3, H-5), 7.28 (d, 2H, *^3^J* = 8.8 Hz, H-2, H-6), 7.21 (br.s, 1H, NH), 2.17 (s, 3H, CH_3_). ^13^C NMR (126 MHz, CDCl_3_) δ 168.39 (1C, CO), 138.06 (2C, C-3, C-5), 137.76 (1C, C-1), 121.76 (2C, C-2, C-6), 87.59 (1C, C-4), 24.82 (1C, CH_3_).
***N*-(4-fluorophenyl)acetamide (8).** To a solution of 4-fluoroaniline (1.00 g, 9.00 mmol) and DIPEA (3.14 mL, 18.0 mmol) in DCM (20 mL) was added acetic anhydride (1 mL, 10.8 mmol) dropwise at 0 °C. The reaction mixture was stirred at room temperature for 1 h. Then, the reaction was quenched with deionised water (5 mL). After decantation, the aqueous layer was extracted with DCM (3 × 10 mL). The combined organic layers were dried on MgSO_4_, filtered, and concentrated under vacuum. The residue was purified by column chromatography (Al_2_O_3_, cyclohexane/EtOAc, 5/5, *v*/*v*) to provide compound **8** (1.36 g, 8.88 mmol, 99%) as a white powder. R_f_ (Al_2_O_3_, cyclohexane/EtOAc, 5/5, *v*/*v*) 0.41. IR (ATR, cm^−1^) 3301, 3271, 1662, 1618, 1556, 1503, 1204, 831. Mp 153 ± 1 °C (Lit. mp 152–153 °C [39]). ^1^H NMR (500 MHz, CDCl_3_) δ 7.89 (br.s, 1H, NH), 7.44 (dd, 2H, *^3^J_H-H_* = 9.0 Hz, *^4^J_H-F_* = 4.8 Hz, H-2, H-6), 6.97 (t, 2H, *^3^J_H-H_* = *^3^J_H-F_* = 8.7 Hz, H-3, H-5), 2.14 (s, 3H, CH_3_). ^13^C NMR (126 MHz, CDCl_3_) δ 168.43 (1C, CO), 159.53 (d, 1C, *^1^J_C-F_* = 244 Hz, C-4), 133.97 (1C, *^4^J_C-F_* = 3 Hz, C-1), 121.92 (d, 2C, *^3^J_C-F_* = 8 Hz, C-2, C-6), 115.77 (d, 2C, *^2^J_C-F_* = 22 Hz, C-3, C-5), 24.56 (1C, CH_3_). ^19^F NMR (470 MHz, CDCl_3_) δ—118.97.
**2-chloro-3-formyl-6-iodoquinoline (9).** Under anhydrous argon atmosphere, POCl_3_ (38 mL, 408 mmol) was added dropwise (over 20 min) to precooled (0 °C) anhydrous DMF (10 mL, 129 mmol). The orange solution was allowed to warm to room temperature (20 min) and then treated with compound **7** (10.0 g, 38.3 mmol). The round-bottom flask was sealed, and the solution was stirred at 80 °C overnight. After cooling to room temperature, the solution was then poured over ice (500 mL). The formed yellow precipitate was filtered, washed with deionised water (100 mL), and dried under vacuum. The crude product was further purified by column chromatography (SiO_2_, cyclohexane/EtOAc, 5/5, *v*/*v*) to provide quinoline **9** (1.53 g, 4.82 mmol, 13%) as a yellow powder. R_f_ (SiO_2_, cyclohexane/EtOAc, 9/1, *v*/*v*) 0.42. IR (ATR, cm^−1^) 1683, 1673, 1596, 1571, 1479, 1360, 829. Mp 169 ± 1 °C. ^1^H NMR (500 MHz, CDCl_3_) δ 10.55 (s, 1H, CHO), 8.64 (s, 1H, H-4), 8.37 (d, 1H, *^4^J* = 2.0 Hz, H-5), 8.11 (dd, 1H, *^3^J* = 8.9 Hz,^*4*^*J* = 2.0 Hz, H-7), 7.80 (d, 1H, *^3^J* = 8.9 Hz, H-8). ^13^C NMR (126 MHz, CDCl_3_) δ 188.90 (1C, CHO), 150.73, 148.64 (2C, C-2, C-3), 142.35 (1C, C-7), 139.00 (1C, C-4), 138.31 (1C, C-5), 130.20 (1C, C-8), 128.27, 127.04 (2C, C-4a, C-8a), 93.80 (1C, C-6). HRMS (ESI) *m*/*z* 317.9171 [M+H]^+^ (calculated for [C_11_H_6_ClINO]^+^ 317.9177), 349.9432 [M+32]^+^ (calculated for [C_11_H_10_ClINO_2_]^+^, 349.9445 methyl hemiacetal form).
**2-chloro-3-formyl-6-fluoroquinoline (10).** Under anhydrous argon atmosphere, POCl_3_ (4.2 mL, 45.7 mmol) was added dropwise to precooled (0 °C) anhydrous DMF (1.25 mL, 16.3 mmol). The solution was allowed to warm to room temperature (15 min) and then treated with compound **8** (1.00 g, 6.53 mmol). The round-bottom flask was sealed, and the solution was stirred at 85 °C for 19 h. After cooling to room temperature, the solution was then poured over ice (100 mL). The yellow precipitate was filtered, washed with deionised water (20 mL), and dried under vacuum to provide compound **10** (182 mg, 0.868 mmol, 13%) as a yellow powder. R_f_ (SiO_2_, cyclohexane/EtOAc, 9/1, *v*/*v*) 0.40. IR (ATR, cm^−1^) 1693 (ν_C=O_), 1581, 1497 (ν_C=C_), 1337 (ν_C-N_), 1220 (ν_C-F_), 1046 (ν_S=O_), 834 (δ_CH op_). Mp 180 ± 1 °C. ^1^H NMR (500 MHz, CDCl_3_) δ 10.38 (s, 1H, CHO), 8.97 (s, 1H, H-4), 8.15-8.10 (m, 2H, H-5, H-7), 7.92 (td, 1H, *^3^J_H-H_* = *^3^J_H-F_* = 8.7 Hz,^*4*^*J_H-H_* = 3.0 Hz, H-7). ^13^C NMR (126 MHz, CDCl_3_) δ 189.08 (1C, CHO), 161.21 (d, 1C, *^1^J_C-F_* = 250 Hz, C-6), 149.60 (d, 1C, *^4^J_C-F_* = 3 Hz, C-8a), 146.82 (1C, C-2 or C-3), 139.65 (d, 1C, *^4^J_C-F_* = 6Hz, C-4), 131.34 (d, 1C, *^3^J_C-F_* = 9 Hz, C-8), 127.49 (d, 1C, *^3^J_C-F_* = 10 Hz, C-4a), 127.05 (1C, C-2 or C-3), 123.98 (d, 1C, *^2^J_C-F_* = 26 Hz, C-7), 112.80 (d, 1C, *^2^J_C-F_* = 22 Hz, C-5). ^19^F NMR (470 MHz, CDCl_3_) δ—110.89. HRMS (ESI) *m*/*z* 210.0113 [M+H]^+^ (calculated for [C_11_H_6_ClFNO]^+^ 210,0116), 242.0375 [M+32] (calculated for [C_11_H_10_ClFNO_2_]^+^ 242.0384, methyl hemiacetal form).
***(R,E)-N*-[(2-chloro-6-iodoquinolin-3-yl)methylene]-2-methylpropane-2-sulfinamide (11).** To a mixture of **9** (600 mg, 1.89 mmol) and (*R*)-2-methylpropane-2-sulfinamide (252 mg, 2.08 mmol) in 1,2-dichloroethane (DCE, 5 mL) was added CuSO_4_ (455 mg, 2.85 mmol). The resulting mixture was stirred at 60 °C for 3 d. The mixture was cooled to room temperature, filtered through a pad of Celite^®^ 545, and rinsed with EtOAc (4 × 17 mL). The filtrate was evaporated under vacuum and purified by column chromatography (SiO_2_, ethanol gradient in DCM from 0 to 5%) to provide **11** (530 mg, 1.26 mmol, 67%) as a yellow powder. R_f_ (SiO_2_, DCM) 0.09. IR (ATR, cm^−1^) 1569, 1475, 1360, 1085, 825. Mp 163 ± 1 °C. ^1^H NMR (500 MHz, CDCl_3_) δ 9.08 (s, 1H, CH=N), 8.72 (s, 1H, H-4), 8.36 (d, 1H,*^4^J* = 1.9 Hz, H-5), 8.05 (dd, 1H, *^3^J* = 8.9 Hz, *^4^J* = 1.9 Hz, H-7), 7.78 (d, 1H, *^3^J* = 8.9 Hz, H-8), 1.32 (s, 9H, CH_3_). ^13^C NMR (126 MHz, CDCl_3_) δ 159.02 (1C, CH=N), 150.72, 147.88 (2C, C-2, C-3), 141.34 (1C, C-7), 137.64 (1C, C-5), 137.52 (1C, C-4), 130.20 (1C, C-8), 128.52, 126.8 (2C, C-4a, C-8a), 93.48 (1C, C-6), 58.74 (1C, *C*(CH_3_)_3_), 22.93 (3C, C(*C*H_3_)_3_). HRMS (ESI) *m*/*z* 420.9624 [M+H]^+^ (calculated for [C_14_H_15_ClIN_2_OS]^+^ 420.9632).
***(R,E)-N*-[(2-chloro-6-fluoroquinolin-3-yl)methylene]-2-methylpropane-2-sulfinamide (12).** To a mixture of **10** (150 mg, 0.716 mmol) and (*R*)-2-methylpropane-2-sulfinamide (96 mg, 0.788 mmol) in DCE (4 mL) was added CuSO_4_ (173 mg, 0.109 mmol). The resulting mixture was stirred at 60 °C for 17 h. The mixture was cooled to room temperature, filtered through a pad of Celite^®^ 545, and rinsed with EtOAc (2 × 10 mL). The filtrate was evaporated under vacuum to provide **12** (194 mg, 0.620 mmol, 87%) as a white powder. R_f_ (SiO_2_, cyclohexane/EtOAc, 9/1, *v*/*v*) 0.17. IR (ATR, cm^−1^) 1564, 1495, 1207, 1082, 829. Mp 155 ± 1 °C. ^1^H NMR (500 MHz, CDCl_3_) δ 9.10 (s, 1H, CH=N), 8.78 (s, 1H, H-4), 8.06 (dd, 1H, *^3^J_H-H_* = 9.2 Hz,*^4^J_H-F_* = 5.1 Hz, H-8), 7.63-7.54 (m, 2H, H-7, H-5), 1.32 (s, 9H, CH_3_). ^13^C NMR (126 MHz, CDCl_3_) δ 161.12 (d, 1C, ^1^*J_C-F_*= 251 Hz, C-6), 159.13 (1C, CH=N), 149.56 (d, 1C, ^4^*J_C-F_*= 3 Hz, C-8a), 146.06 (1C, C-2 or C-3), 138.13 (d, 1C, ^4^*J_C-F_*= 6 Hz, C-4), 131.29 (d, 1C, ^3^*J_C-F_*= 9 Hz, C-8), 127.66 (d, 1C, ^3^*J_C-F_*= 10 Hz, C-4a), 126.85 (1C, C-2 or C-3), 123.96 (d, 1C, ^2^*J_C-F_*= 26 Hz, C-7), 112.04 (d, 1C, ^2^*J_C-F_*= 22 Hz, C-5), 58.70 (1C, *C*(CH_3_)_3_), 22.92 (3C, C(*C*H_3_)_3_). ^19^F NMR (470 MHz, CDCl_3_) δ—111.60. HRMS (ESI) *m*/*z* 313.0568 [M+H]^+^ (calculated for [C_14_H_15_ClFN_2_OS]^+^ 313.0572).
***(R)-N-*[(*S*)-1-(2-chloro-6-iodoquinolin-3-yl)ethyl]-2-methylpropane-2-sulfinamide ((*S*,*R*)-13) and *(R)-N-*[(*R*)-1-(2-chloro-6-iodoquinolin-3-yl)ethyl]-2-methylpropane-2-sulfinamide ((*R*,*R*)-13).** To a solution of **11** (700 mg, 2.38 mmol) in anhydrous DCM (13 mL) was added dropwise, at −60 °C, MeMgBr (3 M solution in diethyl ether, 0.84 mL, 2.51 mmol) under stirring and argon atmosphere. Then, the reaction mixture was stirred at − 60 °C for 3 h and allowed to rise slowly to room temperature overnight. After cooling, a saturated aqueous solution of NH_4_Cl (12 mL) was slowly added. After decantation, the aqueous layer was extracted with DCM (4 × 15 mL). The combined organic layers were dried on MgSO_4_, filtered, and concentrated under reduced pressure. The residue was purified by column chromatography (SiO_2_, cyclohexane/EtOAc, EtOAc gradient varying from 50 to 80%) to provide compound (*S*,*R*)-**13** (452 mg, 1.03 mmol, 62%) and its diastereomer (*R*,*R*)-**13** (110 mg, 0.250 mmol, 15%), both as white solids. **Diastereomer** (*S*,*R*)-**13**: R_f_ (SiO_2_, cyclohexane/EtOAc, 5/5, *v*/*v*) 0.11. IR (ATR, cm^−1^) 3211, 1581, 1475, 1035), 822. Mp 124 ± 1 °C. ^1^H NMR (500 MHz, CDCl_3_) δ 8.20 (d, 1H,^*4*^*J* = 1.9 Hz, H-5), 8.12 (s, 1H, H-4), 7.96 (dd, 1H, *^3^J* = 8.9 Hz, *^4^J* = 1.9 Hz, H-7), 7.74 (d, 1H, *^3^J* = 8.9 Hz, H-8), 5.10 (q, 1H, *^3^J* = 6.7 Hz, CH), 3.47 (br.s, 1H, NH), 1.68 (d, 3H, *^3^J* = 6.7 Hz, CH_3_), 1.26 (s, 9H, C(CH_3_)_3_). ^13^C NMR (126 MHz, CDCl_3_) δ 150.58, 145.91 (2C, C-2, C-3), 139.35 (1C, C-7), 136.50 (1C, C-5), 136.38 (1C, C-4), 135.07 (1C, C-8a or C-4a), 130.01 (1C, C-8), 129.02 (1C, C-8a or C-4a), 92.91 (1C, C-6), 56.26 (1C, *C*(CH_3_)_3_), 52.08 (1C, *C*HCH_3_), 23.58 (1C, CH*C*H_3_), 22.76 (3C, C(*C*H_3_)_3_). HRMS (ESI) *m*/*z* 436.9936 [M+H]^+^ (calculated for [C_15_H_19_ClIN_2_OS]^+^ 436.9945). **Diastereomer** (*R*,*R*)-**13**: R_f_ (SiO_2_, cyclohexane/EtOAc, 5/5, *v*/*v*) 0.18. IR (ATR, cm^−1^) 1581, 1474, 1043, 822. Mp 79 ± 1 °C. ^1^H NMR (500 MHz, CDCl_3_) δ 8.25 (d, 1H,^*4*^*J* = 1,9 Hz, H-5), 8.15 (s, 1H, H-4), 7.95 (dd, 1H, *^3^J* = 8.9 Hz, *^4^J* = 1.9 Hz, H-7), 7.73 (d, 1H, *^3^J* = 8.9 Hz, H-8), 5.02 (qt, 1H, *^3^J* = 6.3 Hz, CH), 3.77 (d, 1H,^*3*^*J* = 5.3 Hz, NH), 1.64 (d, 3H, *^3^J* = 6.6 Hz, CH_3_), 1.25 (s, 9H, C(CH_3_)_3_). ^13^C NMR (126 MHz, CDCl_3_) δ 149.97, 145.77 (2C, C-2, C-3), 139.23 (1C, C-7), 136.63 (1C, C-5), 136.42 (1C, C-4), 134.96 (1C, C-8a or C-4a), 129.78 (1C, C-8), 128.83 (1C, C-8a or C-4a), 92.85 (1C, C-6), 56.16 (1C, *C*(CH_3_)), 51.62 (1C, *C*HCH_3_), 22.63 (1C, C(*C*H_3_)_3_), 22.30 (3C, CH*C*H_3_). HRMS (ESI) *m*/*z* 436.9940 [M+H]^+^ (calculated for [C_15_H_19_ClIN_2_OS]^+^ 436.9945).
***(R)-N-*[(*S*)-1-(2-chloro-6-fluoroquinolin-3-yl)ethyl]-2-methylpropane-2-sulfinamide ((*S*,*R*)-14) and *(R)-N-*[(*R*)-1-(2-chloro-6-fluoroquinolin-3-yl)ethyl]-2-methylpropane-2-sulfinamide ((*R*,*R*)-14).** To a solution of **12** (350 mg, 1.12 mmol) in anhydrous DCM (9 mL) at −60 °C was added dropwise MeMgBr (3 M solution in diethyl ether, 0.56 mL, 1.68 mmol) under stirring and argon atmosphere. Then, the reaction mixture was stirred at −60 °C for 3 h and allowed to rise slowly to room temperature overnight. After cooling, a saturated aqueous solution of NH_4_Cl (15 mL) was added slowly. The aqueous layer was extracted with DCM (3 × 20 mL). The combined organic layers were dried on MgSO_4_, filtered, and concentrated under reduced pressure. The residue was purified by column chromatography (SiO_2_, cyclohexane/EtOAc, 5/5, *v*/*v*) to provide derivative (*S*,*R*)-**14** (242 mg, 0.735 mmol, 66%) and its diastereomer (*R*,*R*)-**14** (41 mg, 0.126 mmol, 11%), both as white solids. **Diastereomer** (*S*,*R*)-**14**: R_f_ (SiO_2_, cyclohexane/EtOAc, 5/5, *v*/*v*) 0.10. IR (ATR, cm^−1^) 3181, 1495, 1211, 1055, 828. Mp 138 ± 1 °C. ^1^H NMR (500 MHz, CDCl_3_) δ 8.18 (s, 1H, H-4), 8.02 (dd, 1H, ^3^*J_H-H_*= 9.2 Hz, ^4^*J_H-F_*= 5.2 Hz, H-8), 7.49 (ddd, 1H, *^3^J_H-H_* = 9.2 Hz, *^3^J_H-F_* = 8.3 Hz, *^4^J_H-H_* = 2.8 Hz, H-7), 7.43 (dd, 1H, *^3^J_H-F_* = 8.6 Hz, *^4^J_H-H_* = 2.8 Hz, H-5), 5.10 (qd, 1H, *^3^J_H-H_* = 4.6 and 6.7 Hz, CH), 3.48 (d, 1H,^*3*^*J_H-H_* = 4.4 Hz, NH), 1.70 (d, 3H, *^3^J_H-H_* = 6.7 Hz, CH_3_), 1.25 (s, 9H, C(CH_3_)_3_). ^13^C NMR (126 MHz, CDCl_3_) δ 160.92 (d, 1C, ^1^*J_C-F_*= 249 Hz, C-6), 149.31 (d, 1C, ^4^*J_C-F_*= 3 Hz, C-8a), 143.99, 136.46 (2C, C-2, C-3), 135.69 (d, 1C, ^4^*J_C-F_*= 5 Hz, C-4), 130.90 (d, 1C, ^3^*J_C-F_*= 9 Hz, C-8), 128.07 (d, 1C, ^3^*J_C-F_*= 10 Hz, C-4a), 120.77 (d, 1C, ^2^*J_C-F_*= 26 Hz, C-7), 110.84 (d, 1C, ^2^*J_C-F_*= 22 Hz, C-5), 56.24 (1C, *C*(CH_3_)_3_), 52.15 (1C, *C*HCH_3_), 23.53 (1C, CH*C*H_3_), 22.74 (3C, C(*C*H_3_)_3_). ^19^F NMR (470 MHz, CDCl_3_) δ—112.88. HRMS (ESI) *m*/*z* 329.0877 [M+H]^+^ (calculated for [C_15_H_19_ClFN_2_OS]^+^ 329.0885). **Diastereomer** (*R*,*R*)-**14**: R_f_ (SiO_2_, cyclohexane/EtOAc, 5/5, *v*/*v*) 0.12. IR (ATR, cm^−1^) 3463, 1496, 1040-1008, 822. Mp 116 ± 1 °C. ^1^H NMR (500 MHz, CDCl_3_) δ 8.20 (s, 1H, H-4), 8.01 (dd, 1H, ^3^*J_H-H_*= 9.2 Hz, ^4^*J_H-F_*= 5.2 Hz, H-8), 7.52-7.43 (m, 2H, H-5, H-7), 5.03 (qt, 1H, *^3^J_H-H_* = 6.5 Hz, CH), 3.81 (d, 1H,^*3*^*J_H-H_* = 5.4 Hz, NH), 1.65 (d, 3H, *^3^J_H-H_* = 6.6 Hz, CH_3_), 1.26 (s, 9H, C(CH_3_)_3_). ^13^C NMR (126 MHz, CDCl_3_) δ 160.95 (d, 1C, ^1^*J_C-F_*= 249 Hz, C-6), 148.90 (d, 1C, ^4^*J_C-F_*= 3 Hz, C-8a), 144.08, 136.63 (2C, C-2, C-3), 135.62 (d, 1C, ^4^*J_C-F_*= 5 Hz, C-4), 130.90 (d, 1C, ^3^*J_C-F_*= 9 Hz, C-8), 128.10 (d, 1C, ^3^*J_C-F_*= 10 Hz, C-4a), 120.88 (d, 1C, ^2^*J_C-F_*= 26 Hz, C-7), 111.00 (d, 1C, ^2^*J_C-F_*= 22 Hz, C-5), 56.26 (1C, *C*(CH_3_)_3_), 51.80 (1C, *C*HCH_3_), 22.35 (1C, CH*C*H_3_), 22.70 (3C, C(*C*H_3_)_3_). ^19^F NMR (470 MHz, CDCl_3_) δ—112.79. HRMS (ESI) *m*/*z* 329.0877 [M+H]^+^ (calculated for [C_15_H_19_ClFN_2_OS]^+^ 329.0885).
***((S)*-3-(1-aminoethyl)-6-iodoquinolin-2*(1H)*-one hydrochloride salt ((*S*)-5).** A mixture of (*S*,*R*)-**13** (450 mg, 1.03 mmol) in 1,4-dioxane (3.5 mL) and aqueous 1 N HCl solution (3.5 mL, 3.52 mmol) was heated at 110 °C overnight. After cooling to room temperature, the mixture was evaporated under reduced pressure, and the yellow powder obtained was washed with diethyl ether (5 × 5 mL) to provide quinolinone (*S*)-**5** (158 mg, quantitative) as a yellow solid, which was used in the next step without further purification. HRMS (ESI) *m*/*z* 314.9981 [M+H]^+^ (calculated for [C_11_H_12_IN_2_O]^+^ 314.9988).
***((R)*-3-(1-aminoethyl)-6-iodoquinolin-2*(1H)*-one hydrochloride salt ((*R*)-5).** Using the same procedure as described for compound (*S*)-**5**, the enantiomer (*R*)-**5** was obtained as a yellow solid. Yield 95%. HRMS (ESI) *m*/*z* 314.9983 [M+H]^+^ (calculated for [C_11_H_12_IN_2_O]^+^ 314.9989).
**((*S*)-3-(1-aminoethyl)-6-fluoroquinolin-2*(1H)*-one hydrochloride salt ((*S*)-6).** Using the same procedure as described for compound (*S*)-**5**, the fluorinated analogue (*S*)-**6** was obtained as a yellow solid. Yield 80%. HRMS (ESI) *m*/*z* 207.0925 [M+H]^+^ (calculated for [C_11_H_12_FN_2_O]^+^ 207.0928).
**((*R*)-3-(1-aminoethyl)-6-fluoroquinolin-2*(1H)*-one hydrochloride salt ((*R*)-6).** Using the same procedure as described for compound (*S*)-**5**, the compound (*R*)-**6** was obtained as a yellow solid. Yield 88%. HRMS (ESI) *m*/*z* 207.0926 [M+H]^+^ (calculated for [C_11_H_12_FN_2_O]^+^ 207.0928).
**(*S*)-5-{[1-(6-iodo-2-oxo-1,2-dihydroquinolin-3-yl)ethyl]-amino}-1-methyl-6-oxo-1,6-dihydropyridine-2-carbonitrile ((*S*)-2)**. DIPEA (750 µL, 4.28 mmol) was added to a solution of **4** (254 mg, 1.67 mmol) and amine (*S*)-**5** (500 mg, 1.43 mmol) in dimethylsulfoxyde (DMSO, 11 mL). The resulting solution was stirred at 110 °C for 15 h. After cooling to room temperature, deionised water (150 mL) was added, and the resulting solution was extracted with EtOAc (3 × 100 mL). The organic extracts were combined, washed with deionised water (200 mL) to eliminate residual traces of DMSO, dried on MgSO_4_, filtered, and concentrated under reduced pressure. The crude product was purified by reversed-phase flash chromatography (flow 30 mL.min^−1^; water/ACN: 95/5, *v*/*v* for 2 min; 95/5 to 0/100, *v*/*v* for 13 min; and 0/100, *v*/*v* for 5 min) to provide end-product (*S*)-**2** (341 mg, 0.764 mmol, 54%) as a yellow powder. R_f_ (SiO_2_, cyclohexane/EtOAc, 3/7, *v*/*v*) 0.35. IR (ATR, cm^−1^) 2208, 1647, 1591, 1544, 809. Mp 172 ± 1 °C. ^1^H NMR (500 MHz, CDCl_3_) δ 12.36 (s, 1H, NH), 7.86 (d, 1H,^*4*^*J* = 1.7 Hz, H-5), 7.76 (dd, 1H, *^3^J* = 8.6 Hz,^*4*^*J* = 1.8 Hz, H-7), 7.59 (s, 1H, H-4), 7.21 (d, 1H,^*3*^*J* = 8.6 Hz, H-8), 6.68 (d, 1H,^*3*^*J* = 7.8 Hz, H-3′), 6.29 (br.s, 1H, NH), 5.90 (d, 1H, *^3^J* = 7.9 Hz, H-4′), 4.85 (q, 1H,^*3*^*J* = 6.6 Hz, C*H*CH_3_), 3.75 (s, 3H, NCH_3_), 1.66 (d, 3H, *^3^J* = 6.7 Hz, CHC*H*_3_). ^13^C NMR (126 MHz, CDCl_3_) δ 163.06 (1C, C-2), 156.94 (1C, C-6′), 141.31 (1C, C-5′ or C-2′), 139.09 (1C, C-7), 137.03 (1C, C-8a or C-4a), 136.45 (1C, C-5), 134.25 (1C, C-3), 134.16 (1C, C-4), 121.96 (1C, C-8a or C-4a), 119.42 (1C, C-3′), 117.70 (1C, C-8), 114.81 (1C, CN), 106.11 (1C, C-5′ or C-2′), 104.60 (1C, C-4′), 85.89 (1C, C-6), 47.91 (1C, *C*HCH_3_), 34.81 (1C, N*C*H_3_), 21.53 (1C, CH*C*H_3_). HRMS (ESI) *m*/*z* 447.0302 [M+H]^+^ (calculated for [C_18_H_16_IN_4_O_2_]^+^ 447.0312). Analytical HPLC characterisation: *R*_t_: 12.49 min, 95.2% purity at 254 and 360 nm.
**(*R*)-5-{[1-(6-iodo-2-oxo-1,2-dihydroquinolin-3-yl)ethyl]-amino}-1-methyl-6-oxo-1,6-dihydropyridine-2-carbonitrile ((*R*)-2).** DIPEA (150 µL, 0.861 mmol) was added to a solution of **4** (52 mg, 0.343 mmol) and amine (*R*)-**5** (100 mg, 0.285 mmol) in DMSO (2.25 mL). The resulting solution was stirred at 110 °C for 22 h. After cooling to room temperature, deionised water (30 mL) was added, and the resulting solution was extracted with EtOAc (3 × 20 mL). The organic extracts were combined, dried on MgSO_4_, filtered, and concentrated under reduced pressure. The crude product was purified first by silica column chromatography (SiO_2_, cyclohexane/EtOAc, 4/6, *v*/*v*) and then by alumina column chromatography (Al_2_O_3_, cyclohexane/EtOAc, EtOAc gradient varying from 80 to 100%, and then methanol gradient in EtOAc from 1 to 5%) to provide derivative (*R*)-**2** (55 mg, 0.123 mmol, 43%) as a green powder. R_f_ (SiO_2_, cyclohexane/EtOAc, 3/7, *v*/*v*) 0.49. IR (ATR, cm^−1^) 2209, 1633, 1589, 1544, 811. Mp 257 ± 1 °C. ^1^H NMR (500 MHz, CDCl_3_) δ 11.92 (s, 1H, NH), 7.85 (d, 1H,^*4*^*J* = 1.8 Hz, H-5), 7.76 (dd, 1H, *^3^J* = 8.6 Hz,^*4*^*J* = 1.8 Hz, H-7), 7.57 (s, 1H, H-4), 7.17 (d, 1H,^*3*^*J* = 8.6 Hz, H-8), 6.68 (d, 1H,^*3*^*J* = 7.8 Hz, H-3′), 6.26 (br.d, 1H, NH), 5.90 (d, 1H, *^3^J* = 7.9 Hz, H-4′), 4.88-4.81 (m, 1H, C*H*CH_3_), 3.75 (s, 3H, NCH_3_), 1.65 (d, 3H, *^3^J* = 6.7 Hz, CHC*H*_3_). ^13^C NMR (126 MHz, CDCl_3_) δ 163.12 (1C, C-2), 156.93 (1C, C-6′), 141.31 (1C, C-5′ or C-2′), 139.09 (1C, C-7), 137.03 (1C, C-8a or C-4a), 136.45 (1C, C-5), 134.20 (1C, C-3), 134.17 (1C, C-4), 121.93 (1C, C-8a or C-4a), 119.42 (1C, C-3′), 117.69 (1C, C-8), 114.81 (1C, CN), 106.11 (1C, C-5′ or C-2′), 104.59 (1C, C-4′), 85.81 (1C, C-6), 47.92 (1C, *C*HCH_3_), 34.80 (1C, NCH_3_), 21.53 (1C, CH*C*H_3_). HRMS (ESI) *m*/*z* 447.0303 [M+H]^+^ (calculated for [C_18_H_16_IN_4_O_2_]^+^ 447.0312). Analytical HPLC characterisation: *R*_t_: 12.52 min, 95.3% and 96.5% purity at 254 and 360 nm, respectively.
**(*S*)-5-{[1-(6-fluoro-2-oxo-1,2-dihydroquinolin-3-yl)ethyl]-amino}-1-methyl-6-oxo-1,6-dihydropyridine-2-carbonitrile ((*S*)-3).** DIPEA (206 µL, 1.18 mmol) was added to a solution of **4** (71 mg, 0.467 mmol) and amine (*S*)-**6** (96 mg, 0.396 mmol) in DMSO (3 mL). The resulting solution was stirred at 110 °C during 19 h. After cooling to room temperature, deionised water (30 mL) was added, and the aqueous layer was extracted with EtOAc (3 × 20 mL). The organic extracts were combined, dried on MgSO_4_, filtered, and concentrated under reduced pressure. The crude product was purified by column chromatography (Al_2_O_3_, cyclohexane/EtOAc, EtOAc gradient varying from 80 to 100%, and then methanol gradient in EtOAc from 1 to 5%) to provide derivative (*S*)-**3** (56 mg, 0.166 mmol, 42%) as a brown powder. R_f_ (Al_2_O_3_, MeOH 3% in EtOAc) 0.34. IR (ATR, cm^−1^) 2209, 1652, 1627, 1594, 1544, 1227, 814. Mp 171 ± 1 °C. ^1^H NMR (500 MHz, CDCl_3_) δ 12.90 (s, 1H, NH), 7.65 (s, 1H, H-4), 7.47 (dd, 1H, *^3^J_H-H_* = 8.9 Hz,^*4*^*J_H-F_* = 4.5 Hz, H-8), 7.27 (td, *^3^J_H-F_* = *^3^J_H-H_* = 8.6 Hz, *^4^J_H-H_* = 2.6 Hz, 1H, H-7), 7.19 (dd, 1H,^*3*^*J_H-F_* = 8.5 Hz, *^4^J_H-H_* = 2.6 Hz, H-5), 6.68 (d, 1H,^*3*^*J_H-H_* = 7.9 Hz, H-3′), 6.38 (br.s, 1H, NH), 5.94 (d, 1H, *^3^J_H-H_* = 7.9 Hz, H-4′), 4.92–4.84 (m, 1H, C*H*CH_3_), 3.75 (s, 3H, NCH_3_), 1.67 (d, 3H, *^3^J_H-H_* = 6.7 Hz, CHC*H*_3_). ^13^C NMR (126 MHz, CDCl_3_) δ 163.25 (1C, C-2), 158.31 (d, 1C, ^1^*J_C-F_*= 243 Hz, C-6), 156.95 (1C, C-6′), 141.39 (1C, C-5′ or C-2′), 134.65 (d, 1C, ^4^*J_C-F_*= 3 Hz, C-4), 134.39 (d, 1C, ^4^*J_C-F_*= 4 Hz, C-8a), 134.38 (1C, C-3), 120.52 (d, 1C, ^3^*J_C-F_*= 9 Hz, C-4a), 119.47 (1C, C-3′), 118.97 (d, 1C, ^2^*J_C-F_*= 25 Hz, C-7), 117.60 (d, 1C, ^3^*J_C-F_*= 8 Hz, C-8), 114.81 (1C, CN), 112.64 (d, 1C, ^2^*J_C-F_*= 23 Hz, C-5), 105.93 (1C, C-5′ or C-2′), 104.53 (1C, C-4′), 47.97 (1C, *C*HCH_3_), 34.76 (1C, NCH_3_), 21.50 (1C, CH*C*H_3_). ^19^F NMR (470 MHz, CDCl_3_) δ—119.21. HRMS (ESI) *m*/*z* 339.1245 [M+H]^+^ (calculated for [C_18_H_16_FN_4_O_2_]^+^ 339.1252). Analytical HPLC characterisation: *R*_t_: 11.27 min, 97.2% and 96.4% purity at 254 and 360 nm, respectively.
**(*R*)-5-{[1-(6-fluoro-2-oxo-1,2-dihydroquinolin-3-yl)ethyl]-amino}-1-methyl-6-oxo-1,6-dihydropyridine-2-carbonitrile ((*R*)-3).** DIPEA (150 µL, 0.861 mmol) was added to a solution of **4** (53 mg, 0.346 mmol) and amine (*R*)-**6** (71 mg, 0.291 mmol) in DMSO (3 mL). The resulting solution was stirred at 110 °C during 19 h. After cooling to room temperature, deionised water (25 mL) was added, and the aqueous layer was extracted with EtOAc (3 × 20 mL). The organic extracts were combined, dried on MgSO_4_, filtered, and concentrated under reduced pressure. The crude product was purified by column chromatography (SiO_2_, cyclohexane/EtOAc, 2/8, *v*/*v*) to provide derivative (*R*)-**3** (39 mg, 0.115 mmol, 40%) as a brown powder. R_f_ (SiO_2_, cyclohexane/EtOAc, 2/8, *v*/*v*) 0.33. IR (ATR, cm^−1^) 2208, 1652, 1626, 1592, 1544, 1227, 815. Mp 166 ± 1 °C. ^1^H NMR (500 MHz, CDCl_3_) δ 12.69 (br.s, 1H, NH), 7.65 (s, 1H, H-4), 7.46 (dd, 1H, *^3^J_H-H_* = 8.9 Hz,^*4*^*J_H-F_* = 4.5 Hz, H-8), 7.27 (td, 1H, *^3^J_H-F_* = *^3^J_H-H_* = 8.4 Hz, *^4^J_H-H_* = 2.8 Hz, H-7), 7.19 (dd, 1H,^*3*^*J_H-F_* = 8.5 Hz, *^4^J_H-H_* = 2.7 Hz, H-5), 6.68 (d, 1H,^*3*^*J_H-H_* = 7.9 Hz, H-3′), 6,34 (br.s, 1H, NH), 5.93 (d, 1H, *^3^J_H-H_* = 7.9 Hz, H-4′), 4.87 (q, 1H, *^3^J_H-H_* = 6.7 Hz, C*H*CH_3_), 3.75 (s, 3H, NCH_3_), 1.67 (d, 3H, *^3^J_H-H_* = 6.7 Hz, CHC*H*_3_). ^13^C NMR (126 MHz, CDCl_3_) δ 163.12 (1C, C-2), 158.35 (d, 1C, ^1^*J_C-F_* = 243 Hz, C-6), 156.96 (1C, C-6′), 141.39 (1C, C-5′ or C-2′), 134.69 (d, 1C, ^4^*J_C-F_* = 3 Hz, C-4), 134.44 (1C, C-3), 134.32 (s, 1C, C-8a), 120.56 (d, 1C, ^3^*J_C-F_* = 9 Hz, C-4a), 119.47 (1C, C-3′), 119.02 (d, 1C, ^2^*J_C-F_* = 25 Hz, C-7), 117.59 (d, 1C, ^3^*J_C-F_* = 8 Hz, C-8), 114.82 (1C, CN), 112.69 (d, 1C, ^2^*J_C-F_* = 23 Hz, C-5), 105.98 (1C, C-5′ or C-2′), 104.55 (1C, C-4′), 47.97 (1C, *C*HCH_3_), 34.77 (1C, N*C*H_3_), 21.53 (1C, CH*C*H_3_). ^19^F NMR (470 MHz, CDCl_3_) δ—119.10. HRMS (ESI) *m*/*z* 339.1245 [M+H]^+^ (calculated for [C_18_H_16_FN_4_O_2_]^+^ 339.1252). Analytical HPLC characterisation: *R*_t_: 12.26 min, 95.6% and 95.9% purity at 254 and 360 nm, respectively.
**5-((S)-1-(6-(tributylstannyl)-1,2-dihydro-2-oxoquinolin-3-yl)ethylamino)-1,6-dihydro-1-methyl-6-oxopyridine-2-carbonitrile ((*S*)-15).** To a solution of iodinated compound (*S*)-**2** (150 mg, 0.336 mmol) in anhydrous propan-2-ol (17 mL), beforehand degassed under argon flow, were successively added, under stirring, tris(dibenzylidenacetone)palladium(0) (7.7 mg, 8.4 µmol), hexabutylditin (187 µL, 0.370 mmol), and DIPEA (146 µL, 0.84 mmol). The reaction mixture was then stirred at room temperature for 21 h before further addition of tris(dibenzylidenacetone)palladium(0) (7.7 mg, 8.4 µmol). After 2 h stirring at room temperature, the mixture was filtered on Celite 545 and washed with EtOAc (3 × 50 mL). After evaporation of the filtrate under reduced pressure, the crude residue was purified by column chromatography (SiO_2_, cyclohexane/EtOAc, 7/3, *v*/*v* and then cyclohexane/EtOAc, 3/7, *v*/*v*) to provide derivative (*S*)-**15** (111 mg, 0.182 mmol, 54%) as a light yellow solid to be stored at 4 °C under argon atmosphere and protected from light. R_f_ (SiO_2_, cyclohexane/EtOAc, 5/5, *v*/*v*) 0.23. IR (ATR, cm^−1^) 2955, 2921, 2870, 1850, 2209, 1650, 1635, 1594, 1548, 815. Mp 82 ± 1 °C. ^1^H NMR (500 MHz, CDCl_3_) δ ^1^H NMR (500 MHz, CDCl_3_) δ 10.94 (s, 1H, NH), 7.67 (s, 1H, H-4), 7.61–7.48 (m, 2H, H-5, H-7), 7.29 (d, 1H,^*3*^*J* = 8.1 Hz, H-8), 6.69 (d, 1H,^*3*^*J* = 7.8 Hz, H-3′), 6.26 (d, 1H, *^3^J* = 6.6 Hz, NH), 5.96 (d, 1H, *^3^J* = 7.8 Hz, H-4′), 4.88 (qt, 1H, *^3^J* = 6.7 Hz, C*H*CH_3_), 3.76 (s, 3H, NCH_3_), 1.64 (d, 3H, *^3^J* = 6.6 Hz, CHC*H*_3_), 1.60–1.45 (m, 6H, Sn(CH_2_C*H*_2_CH_2_CH_3_)_3_, 1.39–1.26 (m, 6H, Sn(CH_2_CH_2_C*H*_2_CH_3_)_3_), 1.15–0.98 (m, 6H, Sn(CH_2_CH_2_C*H*_2_CH_3_)_3_), 0.95–0.84 (m, 9H, Sn(C*H*_2_CH_2_CH_2_C*H*_3_)_3_). ^13^C NMR (126 MHz, CDCl_3_) δ 162.61 (1C, C-2),157.05 (1C, C-6′), 141.50 (1C, C-5′ or C-2′), 138.28 (1C, C-7), 137.52 (1C, C-8a or C-4a), 136.08 (1C, C-5), 135.27 (1C, C-3), 133.16 (1C, C-4), 130.64 (1C, C-6), 120.01 (1C, C-8a or C-4a), 119.67, 119.59 (2C, C-3′, C-8), 114.88 (1C, CN), 105.82 (1C, C-5′ or C-2′), 104.68 (1C, C-4′), 47.67 (1C, *C*HCH_3_), 34.77 (1C, N*C*H_3_), 21.81 (1C, CH*C*H_3_), 27.49 (3C, ^3^*J*_119Sn/117Sn-C_ = 62 Hz, Sn(CH2*C*H2CH2CH3)3), 29,20 (3C, Sn(CH2CH2*C*H2CH3)3), 13.82 (3C, Sn(CH2CH2CH2*C*H3)3), 9.87 (3C, Sn(*C*H2CH2CH2CH3)3). HRMS (ESI) *m*/*z* 611.2399 [M+H]^+^ (calculated for [C_30_H_43_N_4_O_2_^120^Sn]^+^ 611.2403).

### 3.2. Biological Evaluation

#### 3.2.1. Mutant IDH1 Enzyme Assay for Determination of the Inhibitory Potency

Determination of the activity and inhibition of mutant IDH1 recombinant proteins (IDH1 R132H and IDH1 R132C) is based on the reduction of α-KG to D2HG accompanied by a concomitant oxidation of NADPH to NADP. The amount of NADPH remaining at the end of the reaction time is measured in a secondary diaphorase/resazurin reaction in which the NADPH is consumed in a 1:1 molar ratio with the conversion of resazurin to the highly fluorescent resorufin. For the determination of inhibitory potential of the investigated ligands, the IDH1 R132H Assay Kit (BPS-79376, BPS Bioscience, San Diego, CA, USA) and IDH1 R132C from ActiveMotif (ACM-31713 Carlsbad, CA, USA) were used. The enzyme activity assay was performed in a volume of 100 µL Buffer (20 mM TRIS buffer (pH = 7.5), 150 mM NaCl, 10 mM MgCl_2_, 0.05% bovine serum albumin (BSA), and 4 mM β-mercaptoethanol) containing 0.5 ng/µL IDH1 R132H or IDH1 R132C enzyme, 2 mM α-KG, and 12 μM NADPH. For inhibition assays, triplicate samples of the compounds in the concentration range from 10^−5^ to 10^−10^ M were incubated with the enzyme for 30 min prior to addition of α-KG and NADPH to initiate the reaction. The reaction ran for 60 min at room temperature and was terminated with the addition of 25 μL of Detection Buffer (36 μg/mL diaphorase, 30 mM resazurin) to 50 µL of the reaction solution. The conversion of resazurin to resorufin by diaphorase was measured fluorometrically at Ex544/Em590 (Synergy H1 microplate reader, BioTek. Winooski, VT, USA). The data were imported into GraphPad Prism 4.1 (GraphPad Inc.; La Jolla; CA, USA), and the IC_50_ values were calculated with a standard dose–response curve fitting (Figure S3).

#### 3.2.2. wt IDH Enzyme Assay for Determination of Inhibitory Potency

wt IDH enzyme assay is a modified version of the assay used for mIDH1. With the conversion of isocitrate to α-KG, this enzyme stoichiometrically converts NADP to NADPH. The produced NADPH, which directly couples to the diaphorase/resazurin system and the resorufin production, can be measured. The protocols for the determination of inhibition of both wt IDH1 and wt IDH2 were conducted as described by Wang et al. [40]. The wt IDH1 (ab113858) was purchased from abcam (Cambridge, UK), and the wt IDH2, from BPS Bioscience (San Diego, CA, USA).

Briefly, wt IDH1 assays were conducted in 50 µL buffer (20 mM TRIS buffer (pH = 7.5), 150 mM NaCl, 10 mM MgCl_2_, 0.05% BSA, and 4 mM β-mercaptoethanol) containing 50 µM NADP, 70 µM D/L-isocitrate, and 0.04 µg/mL wt IDH1 enzyme (reaction time 1 h at RT). For inhibition assays, triplicate samples of the compounds in the concentration range from 10^−5^ to 10^−10^ M were incubated with the wt IDH1 for 1 h before addition of D/L-isocitrate and NADP to initiate the reaction together with the direct detection system comprising 20 µg/mL diaphorase and 4 µM resazurin. The reaction was terminated by addition of 25 μL of 2% SDS and read on a Synergy H1 microplate reader at Ex544/Em590.

Assays for wt IDH2 were conducted in 50 µL buffer (20 mM TRIS buffer (pH = 7.5), 150 mM NaCl, 10 mM MgCl_2_, 0.05% BSA, and 4 mM β-mercaptoethanol) containing 50 µM NADP, 70 µM D/L-isocitrate, and 0.05 µg/mL wt IDH2 enzyme (reaction time 1 h at RT). For inhibition assays, triplicate samples of the compounds in the concentration range from 10^−5^ to 10^−10^ M were incubated with the wt IDH2 and NADP for 1 h before addition of D/L-isocitrate to initiate the reaction together with the direct detection system comprising 20 µg/mL diaphorase and 4 µM resazurin. The reaction was stopped by addition of 5 μL 10% SDS and read as described above. The data were imported into GraphPad Prism, and the IC_50_ values were calculated with a standard dose−response curve fitting.

### 3.3. Radiochemistry with Iodine-125

#### 3.3.1. General Information

[^125^I]NaI (3.58 GBq/mL, 643.8 GBq/mg) was purchased from PerkinElmer Life and Analytical Sciences (331 Treble Cove Road, Billerica, MA 01862, USA) as a no-carrier-added solution in reductant-free 1.0 × 10^−5^ M aqueous sodium hydroxide solution (pH 8–11). The radio TLC strips (Macherey-Nagel, Precoated TLC sheets ALUGRAM^®^ Xtra SIL G/UV_254_) were developed with EtOAc/cyclohexane (65/35, *v*/*v*) and measured on a miniGITA Dual radio-TLC instrument (Elysia-Raytest). Semipreparative RP-HPLC purifications were performed on a Perkin Elmer system equipped with a Flexar LC autosampler, a Series 200 pump, a Peltier column oven, a vacuum degasser, a photodiode array detector (PAD), and a GabiStar detector (Raytest). The separation was carried out on a C-18 column (Agilent Zorbax, 5 μm, 4.6 × 150 mm) using the following conditions: water containing 0.1% of trifluoroacetic acid (solvent A) and ACN containing 0.1% of trifluoroacetic acid (solvent B); 0 to 1 min: isocratic elution 80% A; 1 to 11 min: gradient elution 80% → 0% A; 11 to 21 min: isocratic elution 0% A; 21 to 26 min: gradient elution 0% → 80%; 26 to 30 min: isocratic elution 80% A with a flow rate of 1.1 mL/min, λ = 254 and 360 nm. Analytical HPLC measurements were performed on a system consisting of a HP1100 (Hewlett Packard, Les Ulis, France) and a Flo-one A_500_ Radiomatic detector (Packard, Canberra, Australia). The separation was carried out on a C-18 column (Agilent Zorbax Extend C18, 5 μm, 4.6 × 150 mm, equipped with a guard column) using the following solvent conditions: water containing 0.1% of trifluoroacetic acid (solvent A) and ACN containing 0.1% of trifluoroacetic acid (solvent B); 0 to 3 min: isocratic elution 95% A; 3 to 15 min: gradient elution 95% → 5% A; 15 to 25 min: isocratic elution 5% A with a flow rate of 1 mL/min, λ = 254 and 330 nm. Sep-Pak^®^ C18 Plus Light cartridges (130 mg, 55–105 µm) were purchased from Waters. All radiolabelled compounds were compared by TLC or analytical HPLC to the authentic nonradioactive material and found to be free of significant UV-absorbing chemical and radiochemical impurities.

#### 3.3.2. Radiolabelling with Iodine-125

To a solution of organotin precursor (*S*)-**15** in methanol (50 µL, 1 mg/mL) were successively added [^125^I]NaI (10-50 µL, 2.1–15.3 MBq), glacial acetic acid (2.5 µL), and a solution of CAT trihydrate (5 µL, 1 mg/mL). The reaction vial was sealed and stirred at room temperature for 5 min before addition of a solution of Na_2_S_2_O_5_ in deionised water (5 µL, 1 mg/mL). The reaction mixture was purified by semipreparative RP-HPLC. The collected fraction was further diluted with deionised water (10 mL) and passed through a Sep-Pak^®^ Plus Light cartridge (beforehand conditioned successively with 10 mL of deionised water, 10 mL of methanol, and 10 mL of deionised water). The cartridge was washed with deionised water (2 mL) and dried with air before elution of the radioiodinated tracer (*S*)-[^125^I]**2** with ethanol (500 µL). The radiotracer was finally formulated in saline or DPBS (pH 7.4) to reach an ethanol content of ca. 5% for stability studies.

### 3.4. Radiochemistry with Fluorine-18

#### 3.4.1. General Information

No-carrier-added fluorine-18 was produced by Curium Pharma via the [^18^O(p, n)^18^F] nuclear reaction by irradiation of a 2.8 mL > 97%-enriched [^18^O]H_2_O target (Bruce Technology) on a PETtrace cyclotron (16 MeV proton beam, GE healthcare). The radio TLC strips (Macherey-Nagel, Precoated TLC sheets ALUGRAM^®^ Xtra SIL G/UV_254_) were developed with EtOAc/cyclohexane (80/20, *v*/*v*) and measured on a miniGITA Dual radio-TLC instrument (Elysia-Raytest). Analytical HPLC measurements were performed on a system consisting of an Agilent HP series 1100 (Hewlett Packard, Les Ulis, France) combined with a Flo-one A500 Radiomatic detector (Packard, Canberra, Australia). The separation was carried out on a C-18 column (Agilent Zorbax Extend C18, 5 μm, 4.6 × 150 mm, equipped with a guard column) using the following solvent conditions: water containing 0.1% of trifluoroacetic acid (solvent A) and ACN containing 0.1% of trifluoroacetic acid (solvent B); 0 to 3 min: isocratic elution 95% A; 3 to 15 min: gradient elution 95% → 5% A; 15 to 25 min: isocratic elution 5% A with a flow rate of 1 mL/min, λ = 254 and 330 nm. Preparation of anhydrous [^18^F]LiF was performed using a SynChrom R&D EVOI synthesis module (Raytest). The semipreparative HPLC purifications were performed on a Perkin Elmer system equipped with a Flexar LC autosampler, a Series 200 pump, a Peltier column oven, a vacuum degasser, a PAD, and a GabiStar detector (Raytest). The separation was carried out on a Waters Symmetryprep C18 column (300 × 7.8 mm; 7 µm; Waters) using the following conditions: water containing 0.1% of trifluoroacetic acid (solvent A) and ACN containing 0.1% of trifluoroacetic acid (solvent B); 0 to 0.5 min: isocratic elution 80% A; 1 to 16 min: gradient elution 80% → 0% A; 16 to 23 min: isocratic elution 0% A; 23 to 25 min: gradient elution 0% → 80% A with a flow rate of 2.5 mL/min, λ = 254 and 360 nm. Sep-Pak^®^ Light Accell Plus QMA carbonate cartridges (130 mg, 37–55 µm) and Sep-Pak^®^ C18 Plus Light cartridges (130 mg, 55–105 µm) were purchased from Waters. All radiolabelled compounds were compared by TLC or analytical HPLC to the authentic nonradioactive material and to be free of significant UV-absorbing chemical and radiochemical impurities.

#### 3.4.2. Radiolabelling with Fluorine-18

The aqueous solution of [^18^F]F^−^ in [^18^O]H_2_O was passed through an anion exchange resin (Sep-Pak^®^ Light Accell Plus QMA carbonate cartridge) preconditioned with 10 mL of ethanol, 10 mL of an aqueous solution of lithium trifluoromethanesulfonate (90 mg/mL), and 10 mL of deionised water. Then, a solution of potassium carbonate (50 µg) and lithium trifluoromethanesulfonate (10 mg) in water (550 µL) was passed through the cartridge to elute the radioactivity to the reactor before addition of anhydrous ACN (1 mL). In such conditions, more than 97% of the radioactivity was usually recovered from the cartridge. The solution was dried by azeotropic distillation under reduced pressure and He flow at 100 °C for 12 min. After cooling to 25 °C, anhydrous DMA (2 mL) was added to the reactor. Then, 200 µL of the resulting solution (40–245 MBq, n = 10) was transferred in a glass vial equipped with a magnetic stirrer before addition of a solution of tetrakis(pyridine)copper(II) triflate (Cu(OTf)_2_(py)_4_, 14 mg, 20.6 µmol) in anhydrous DMA (250 µL) and a solution of the tin precursor (*S*)-**15** (6.1 mg, 10.0 µmol) in anhydrous DMA (100 µL). The reaction mixture was stirred at 110 °C for 10 min. After cooling to 25 °C, deionised water (25 mL) was added, and the reaction mixture was passed through a Sep-Pak^®^ C18 Plus Light cartridge preconditioned with 10 mL of deionised water, 10 mL of methanol, and 10 mL of deionised water. The cartridge was washed with deionised water (2 mL) and flushed with air before elution of the desired ^18^F-labelled radiotracer with ACN (500 µL). For isolation of the radiotracer, the eluate was subjected to semipreparative RP-HPLC. The collected fraction was diluted with deionised water (25 mL) and passed through a Sep-Pak^®^ C18 Plus Light cartridge preconditioned with 10 mL of deionised water, 10 mL of methanol, and 10 mL of deionised water. The cartridge was washed with deionised water (2 mL) and flushed with air before elution of the final (*S*)-[^18^F]**3** radiotracer with ethanol (500–600 µL). The radiotracer was finally formulated in saline or DPBS (pH 7.4) to reach an ethanol content of ca. 5% for stability studies.

## 4. Conclusions

Two novel iodinated and fluorinated derivatives, analogues of the FT-2102 mIDH1 inhibitor, were successfully synthesised together with their respective enantiomers to allow the determination of the *e.e.* at the end of the syntheses. First, biological screening in vitro revealed that the iodinated compound (*S*)-**2** presented similar inhibitory potency on IDH1 mutants (4.88 ± 1.63 on mIDH1 R132H) and high selectivity towards the wt IDH1/2 enzymes compared with the reference FT-2102. The fluorinated derivative (*S*)-**3** presented a slightly lower inhibitory potency, albeit still in the nanomolar range (22.7 ± 5.2 nM on mIDH1 R132H), together with an excellent selectivity. The corresponding radioiodinated and radiofluorinated tracers (*S*)-[^125^I]**2** and (*S*)-[^18^F]**3** were then successively produced starting from a common organotin precursor. Both radiotracers were stable in all incubation media tested (at room temperature in ethanol, DPBS pH 7.4, and NaCl 0.9% and at 37 °C in mouse serum). In the next steps, these radiotracers will be further evaluated in vitro and in vivo in gliomas and CHS models to assess their potential for targeting mIDH1 by SPECT or PET imaging.

## Figures and Tables

**Table 1:** Overview of synthesised derivatives and their inhibition potency towards mIDH1 and wt IDH1/2.

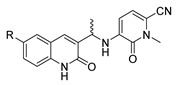					
		IC_50_ (nM) ^a^			
Compound	R	IDH1 R132H	IDH1 R132C	wt IDH1	wt IDH2
(*S*)-FT-2102	Cl	4.88 ± 1.63	178 ± 70	>10,000	>10,000
(*S*)-**2**	I	5.23 ± 0.75	160 ± 24	>10,000	>10,000
(*R*)-**2**	I	323 ± 83	>10,000	>10,000	>10,000
(*S*)-**3**	F	22.7 ± 5.2	579 ± 110	>10,000	>10,000
(*R*)-**3**	F	698 ± 145	>10,000	>10,000	>10,000

^a^ Enzymatic IC_50_ values for mIDH1 and wt IDH1/2 are the mean of at least three determinations performed as described in Material and Methods (mean value ± standard deviation).

## Data Availability

Not applicable.

## Supplementary Materials

The following supporting information can be downloaded at: https://www.mdpi.com/article/10.3390/molecules27123766/s1. Experimental procedures for the synthesis of compound **4**, Figure S1: CD and UV spectra of compound **4**, Figure S2: CD and UV spectra of (*S*)- and (*R*)-binaphthol used as standard reference, Figure S3: Representative inhibitory curves for A) IDH1 R132H and B) IDH1 R132C, Table S1: HPLC conditions and retention times for the chiral separation of the compounds, ^1^H NMR and ^13^C NMR spectra for all synthesised compounds.
