# Supplementary material for: Novel Radioiodinated and Radiofluorinated Analogues of FT-2102 for SPECT or PET Imaging of mIDH1 Mutant Tumours
Source: Molecules. 2022 Jun 11;27(12):3766. doi: 10.3390/molecules27123766 (PMC9228733; doi:10.3390/molecules27123766)
Supplement: Supplementary file 1 [file molecules-27-03766-s001.zip › molecules-1759310-supplementary.pdf]

Supporting information

Novel Radioiodinated and Radiofluorinated Analogues of FT-2102 for SPECT or PET Imaging of mIDH1 Mutant Tumours

Valérie Weber 1,†, Lucie Arnaud 1,†, Sladjana Dukic-Stefanovic 2, Barbara Wenzel 2, Valérie Roux 1, Jean-Michel Chezal 1, Thu-Hang Lai 2,3, Rodrigo Teodoro 2, Klaus Kopka 2,4, Elisabeth Miot-Noirault 1, Winnie Deuther-Conrad 2,* and Aurélie Maisonial-Besset 1,*

1 Université Clermont Auvergne, Inserm, Imagerie Moléculaire et Stratégies Théranostiques, UMR 1240, F-63000 Clermont-Ferrand, France

2 Helmholtz-Zentrum Dresden-Rossendorf, Institute of Radiopharmaceutical Cancer Research, Research Site Leipzig, 04318 Leipzig, Germany

3 Department of Research and Development, ROTOP Pharmaka GmbH, 01328 Dresden, Germany

4 Technical University Dresden, School of Science, Faculty of Chemistry and Food Chemistry, 01062 Dresden, Germany

† Both authors contributed equally to this work.

Table of content

1) Chemistry

1.1. General information

1.2. Synthesis of 5-fluoro-6-methyl-1,6-dihydropyridine-2-carbonitrile (**4**)

2) Determination of enantiomeric excess and circular dichroism

Figure S1. CD and UV spectrum of compound **4**

Figure S2. CD and UV spectrum of (*S*)- and (*R*)-binaphthol used as standard reference

Table S1. HPLC conditions and retention times for the chiral separation of the compounds

3) Mutant IDH1 enzyme assay for determination of inhibitory potency

Figure S3. Representative inhibitory curves for A) IDH1 R132H and B) IDH1 R132C

4) ^{1}H NMR and ^{13}C NMR Spectra for all synthesized compounds

1) Chemistry

1.1) General information

All commercially available reagents and solvents were purchased at the following commercial suppliers: Sigma Aldrich, Alpha Aesar, ABX, Acros Organics, Fisher Scientific or Carlo Erba Reagents, ABCR, Bachem. All solvents were dried using common techniques [1]. Unless otherwise noted, moisture sensitive reactions were conducted under dry argon atmosphere. Temperatures indicated in the protocols correspond to the temperature of the oil bath. Analytical thin layer chromatographies (TLC) were performed on precoated silica gel 60 F254 or neutral aluminium oxide 60 F254 plates (Merck or Macherey-Nagel) and visualized with UV light (254 nm) and/or developed with iodine. Flash column chromatography was performed on silica gel 60A normal phase, 35–70 µm (Merck or SDS) or neutral aluminium oxide 90 standardized 63-200 µm (Merck, column chromatographic adsorption analysis acc. to Brockmann). Uncorrected melting points (mp) were recorded on an electrothermal capillary Digital Melting Point Apparatus IA9300 (Bibby Scientific). Nuclear magnetic resonance (NMR) spectra (500 MHz for ^{1}H and 126 MHz for ^{13}C) were recorded on Bruker Avance 500 instrument with chemical shift values (δ) expressed in parts per million (ppm) relative to residual solvent as standard and coupling constants (*J*) are given in Hz. Infrared spectra (IR) were recorded in the range of 4000–440 cm^{-1} on a Nicolet IS10 (Fisher Scientific) with attenuated total reflectance (ATR) accessory.

1.2) Synthesis of 5-fluoro-6-methyl-1,6-dihydropyridine-2-carbonitrile (4).

2-cyano-5-fluoropyridine-1-oxide. A solution of 5-fluoropicolinonitrile (0.53 g, 4.34 mmol) in DCE (4 mL) was added dropwise to a solution of 3-chloroperoxybenzoic acid (*m*-CPBA, < 77 %, 1.52 g, 6.78 mmol) in DCE (11 mL). The solution was stirred at 60 °C for 4 d. Then, *m*-CPBA (< 77%, 0.75 g, 3.35 mmol) was added and the reaction mixture was then refluxed for 1 d. After cooling to room temperature, sodium sulfite (1.67 g, 13.2 mmol) and DCM (5 mL) were added. After stirring at room temperature for 3 h, the resulting solution was diluted with DCM (15 mL), filtered, and the white precipitate collected was washed with DCM (30 mL). The filtrate was washed with a saturated aqueous $NaHCO_3$ solution (30 mL). After decantation, the aqueous layer was extracted with DCM (2 x 30 mL). The organic layers were combined, dried on $MgSO_4$, filtered, and evaporated under vacuum. The crude product was purified by column chromatography (Al_2O_3, cyclohexane/EtOAc, 5/5, v/v) to provide intermediate 2-cyano-5-fluoropyridine-1-oxide (350 mg, 2.53 mmol, 58%) as a white powder. R_f (Al_2O_3, cyclohexane/EtOAc, 5/5, v/v) 0.36. IR (ATR, cm^{-1}) 2244, 1609, 1497, 1309, 1178, 854, 828. Mp 144 ± 1 °C. ^{1}H NMR (500 MHz, $CDCl_3$) δ 8.22 (dd, 1H, $^{3}J_{H-F}$ = 4.2 Hz, $^{4}J_{H-H}$ = 2.2 Hz, H-6), 7.68 (dd, 1H, $^{3}J_{H-H}$ = 9.0 Hz, $^{4}J_{H-F}$ = 6.5 Hz, H-3), 7.15 (ddd, 1H, $^{4}J_{H-H}$ = 2.2 Hz, $^{3}J_{H-F}$ = 6.4 Hz, $^{3}J_{H-H}$ = 8.9 Hz, H-4). ^{13}C NMR (126 MHz, $CDCl_3$) δ 162.30 (d, 1C, $^{1}J_{C-F}$ = 263 Hz, C-5, 131.48 (d, 1C, $^{3}J_{C-F}$ = 11 Hz, C-3), 130.97 (d, 1C, $^{2}J_{C-F}$ = 36 Hz, C-6), 123,55 (1C, C-2), 113.47 (d, 1C, $^{2}J_{C-F}$ = 21 Hz, C-4), 111.12 (1C, CN).

6-cyano-3-fluoropyridin-2-yle acetate. A mixture of 2-cyano-5-fluoropyridine-1-oxide (300 mg, 2.17 mmol) in acetic anhydride (5 mL) was refluxed (150 °C) for 3 d. After cooling to room temperature, the solution was concentrated under reduced pressure. The residue was dissolved in MeOH (5 mL) and stirred for 1 h at room temperature. The solution was evaporated under vacuum and purified by column chromatography (SiO_2, cyclohexane/EtOAc, 7/37, v/v) to yield compound 6-cyano-3-fluoropyridin-2-yle acetate (220 mg, 1.22 mmol, 56%) as a white powder. R_f (SiO_2, cyclohexane/EtOAc, 6/4, v/v) 0.64. IR (ATR, cm^{-1}) 3088, 2247, 1774, 1587, 1460, 1183, 1107, 857. Mp 77 ± 1 °C. ^{1}H NMR (500 MHz, $CDCl_3$) δ 7.71 (dd, 1H, $^{4}J_{H-F}$ = 3.5 Hz, $^{3}J_{H-H}$ = 8.2 Hz, H-3), 7.68 (dd, 1H, $^{3}J_{H-F}$ = 16.0 Hz, $^{3}J_{H-H}$ = 8.2 Hz, H-4), 2.41 (s, 3H, CH_3). ^{13}C NMR (126 MHz, $CDCl_3$) δ 167.27 (s, 1C, CO), 152.80 (d, 1C, $^{1}J_{C-F}$ = 270 Hz,

C-5), 147.66 (d, 1C, $^{2}J_{C-F}$ = 16 Hz, C-6), 129.37 (d, 1C, $^{3}J_{C-F}$ = 5 Hz, C-3), 127.08 (d, 1C, $^{4}J_{C-F}$ = 6 Hz, C-2), 126.68 (d, 1C, $^{2}J_{C-F}$ = 18 Hz, C-4), 115.67 (1C, CN), 20.56 (1C, CH_3).

5-fluoro-6-oxo-1,6-dihydropyridine-2-carbonitrile. To a solution of 6-cyano-3-fluoropyridin-2-yle acetate (200 mg, 1.11 mmol) in MeOH (3 mL) was added potassium carbonate (313 mg, 2.27 mmol). The mixture was stirred at room temperature for 4 h before evaporation under reduced pressure. After dissolution of the residue in water (15 mL), the resulting solution was acidified to pH < 1 by addition of a 1 M aqueous HCl solution (ca. 8 mL) and extracted with EtOAc (5 x 10 mL). The combined organic extracts were dried on $MgSO_4$, filtered, and evaporated under vacuum to provide compound 5-fluoro-6-oxo-1,6-dihydropyridine-2-carbonitrile (147 mg, 1.07 mmol, 98%) as a white solid. R_f (SiO_2, cyclohexane/EtOAc, 7/3, v/v) 0.15. IR (ATR, cm^{-1}) 1651, 1611, 1573 ($\nu_{C=C}$), 1245, 1175, 836 ($\delta_{CH\ op}$). Mp 204 ± 1 °C. ^{1}H NMR (500 MHz, DMSO-d_6): δ 12.87 (s, 1H, H-1), 7.70 (m, 1H, H-4), 7.40 (d, 1H, $^{3}J_{H-H}$ = 3.7 Hz, H-3). ^{13}C NMR (126 MHz, DMSO-d_6) δ 154.53 (d, 1C, $^{2}J_{C-F}$ = 18 Hz, C=O), 151.17 (d, 1C, $^{1}J_{C-F}$ = 253 Hz, C-5), 123.68 (1C, C-4), 120.65 (1C, C-3), 115.67 (1C, CN), 30.72 (1C, C-2).

5-fluoro-1-methyl-6-oxo-1,6-dihydropyridine-2-carbonitrile (<u>4</u>). In a sealed vial, a mixture of 5-fluoro-6-oxo-1,6-dihydropyridine-2-carbonitrile (108 mg, 0.780 mmol) and potassium carbonate (228 mg, 1.65 mmol) in DMF (2 mL) was stirred for 20 min at room temperature. A solution of MeI (55 µL, 0.88 mmol) in DMF (0.5 mL) was added, and the mixture was stirred at room temperature during 2.5 h. The solution was filtered and the filtrate was evaporated under reduced pressure. The residue was purified by column chromatography (SiO_2, cyclohexane/EtOAc, 7/3, v/v) to provide derivative **<u>4</u>** (92.6 mg, 0.609 mmol, 78%) as a white powder. R_f (SiO_2, cyclohexane/EtOAc, 7/3, v/v) 0.21. IR (ATR, cm^{-1}) 2225, 1660, 1616, 1249, 1208, 858. Mp 109 ± 1 °C. ^{1}H NMR (500 MHz, DMSO-d_6) δ 7.51 (dd, 1H, $^{3}J_{H-F}$ = 9.4 Hz, $^{3}J_{H-H}$ = 7.8 Hz, H-4), 7.16 (dd, 1H, $^{3}J_{H-H}$ = 7.8 Hz, $^{4}J_{H-F}$ = 4.7 Hz, H-3), 3.60 (s, 3H, CH_3). ^{13}C NMR (126 MHz, DMSO-d_6) δ 154.83 (d, 1C, $^{1}J_{C-F}$ = 257 Hz, C-5), 154.57 (d, 1C, $^{2}J_{C-F}$ = 27 Hz, C-6), 119.36 (d, 1C, $^{2}J_{C-F}$ = 20 Hz, C-4), 117.20 (d, 1C, $^{4}J_{C-F}$ = 5 Hz, C-2), 114.76 (d, 1C, $^{4}J_{C-F}$ = 7 Hz, C-3), 112.93 (d, 1C, $^{5}J_{C-F}$ = 2 Hz, CN), 34.60 (1C, $^{4}J_{C-F}$ = 2 Hz, CH_3).

2) Determination of enantiomeric excess and circular dichroism

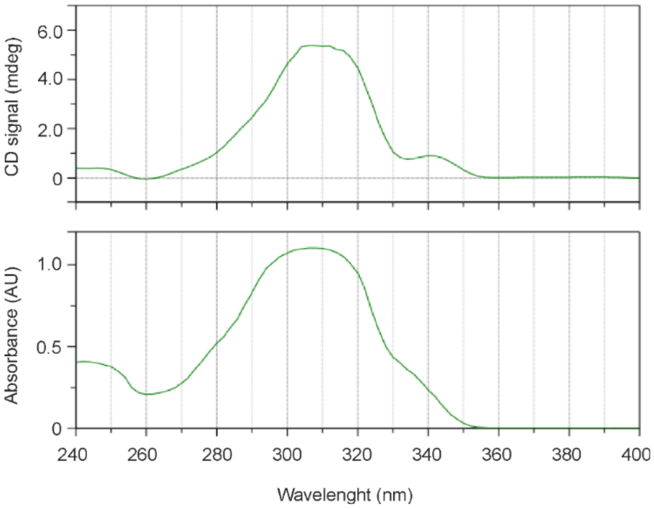

Figure S1. CD and UV spectra of compound **4**

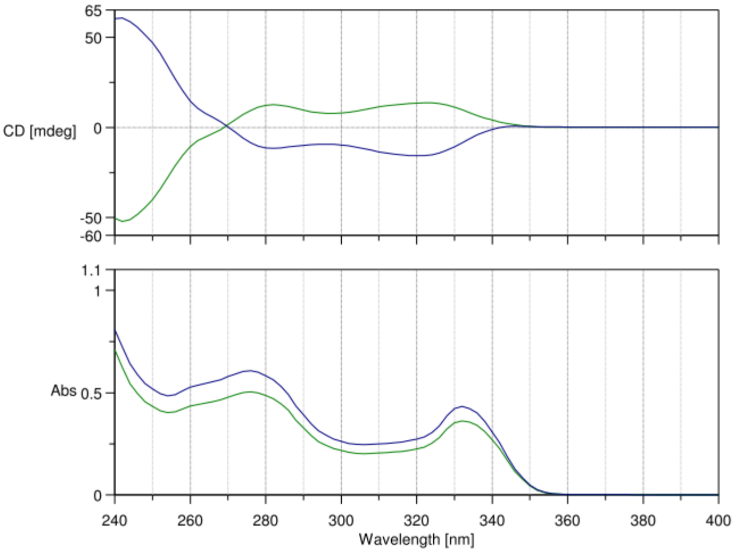

Figure S2. CD and UV spectrum of (*S*)- and (*R*)-binaphthol used as standard reference

Table S1. HPLC conditions and retention times for the chiral separation of the compounds

Compound	ACN/20 mM NH_4OAc (v/v)	Retention time (min)
(*S*)-**2**	46:54	24.02
(*R*)-**2**	46:54	22.71
(*S*)-**3**	34:66	32.66
(*R*)-**3**	34:66	30.17
(*S*)-**FT-2102**	38:62	29.44
(*R*)-**FT-2102**	38:62	28.09
**4**	30:70	6.41

3) Mutant IDH1 enzyme assay for determination of inhibitory potency

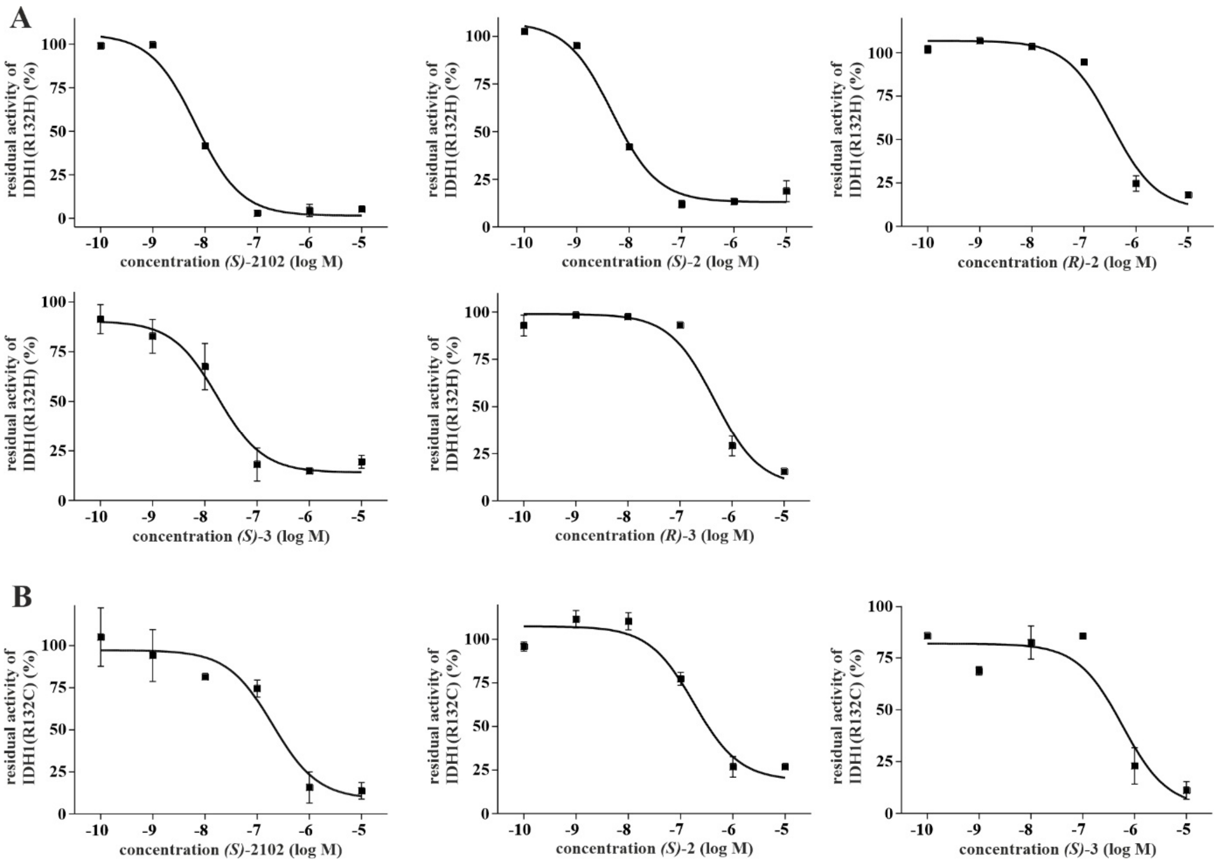

Figure S3. Representative inhibitory curves for A) IDH1 R132H and B) IDH1 R132C

Reference

1. Armarego, W.L.F.; Chai, C.L.L. *Purification of Laboratory Chemicals*; 6. ed.; Elsevier, Butterworth-Heinemann: Amsterdam, 2009; ISBN 978-1-85617-567-8.

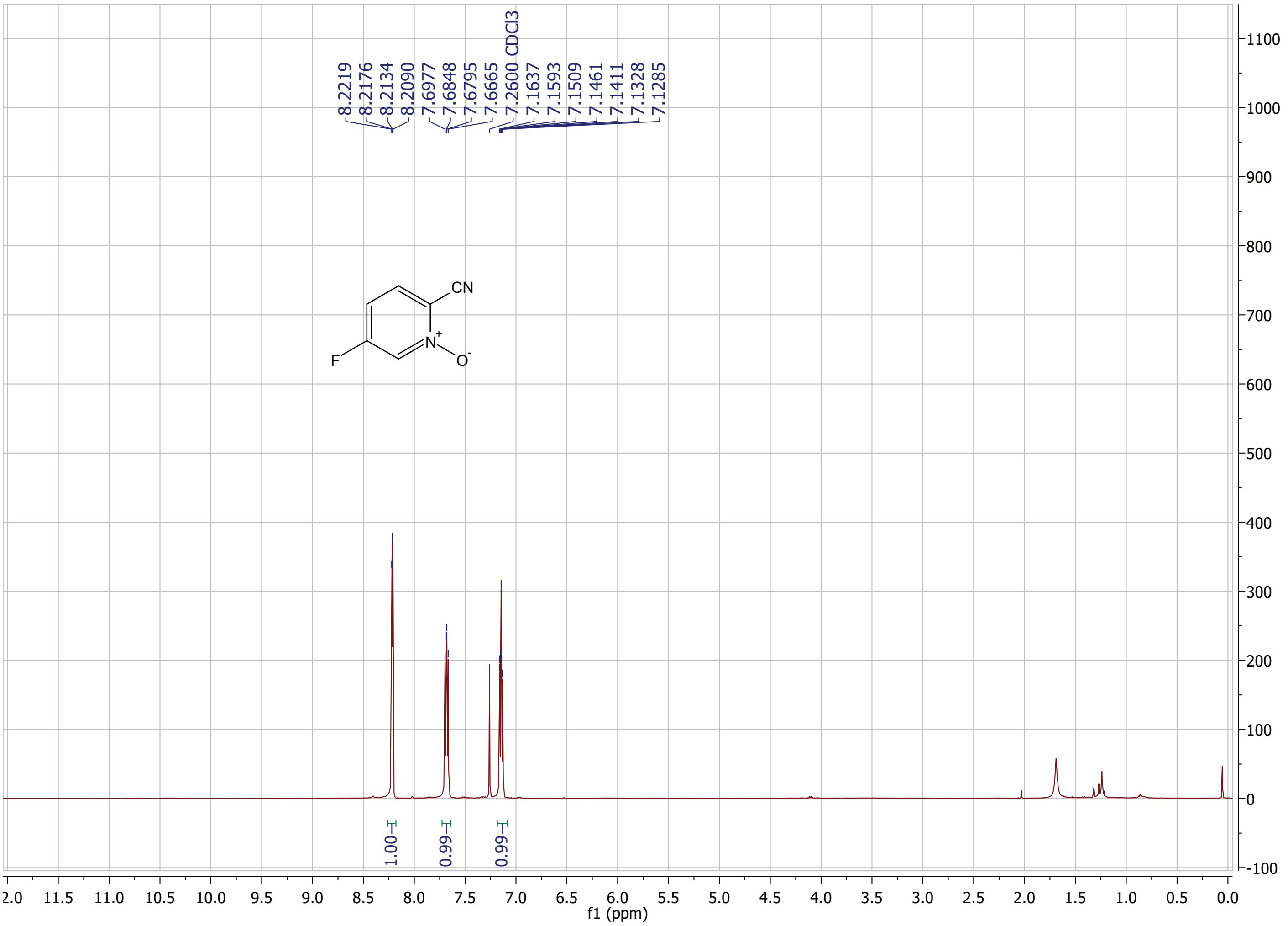
8.2219
8.2176
8.2134
8.2090
7.6977
7.6848
7.6795
7.6665
7.2600 CDCl3
7.1637
7.1593
7.1509
7.1461
7.1411
7.1328
7.1285
CN
N+
O-
F
1.00
0.99
0.99
f1 (ppm)

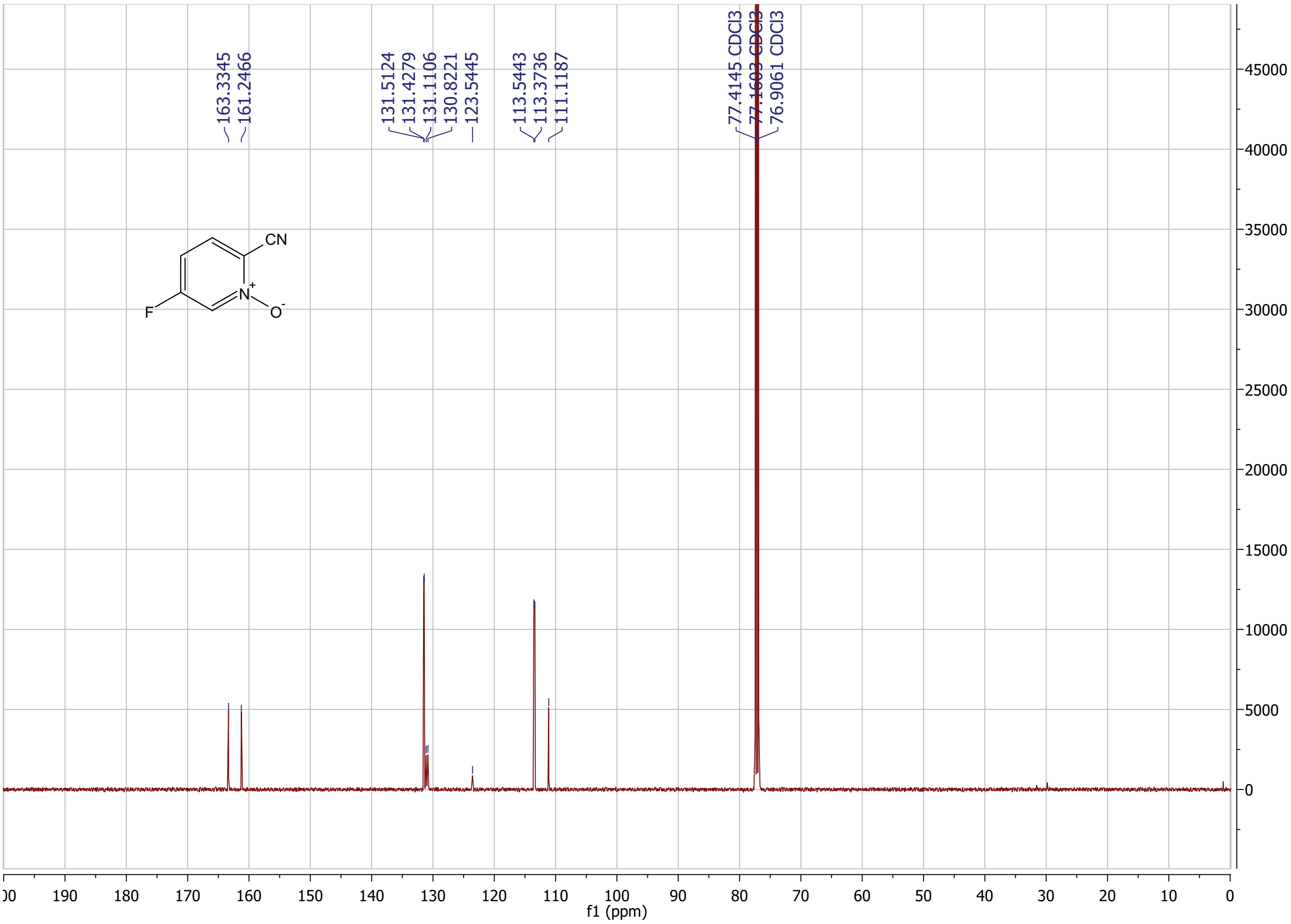
163.3345
161.2466
131.5124
131.4279
131.1106
130.8221
123.5445
113.5443
113.3736
111.1187
77.4145 CDCl3
77.1603 CDCl3
76.9061 CDCl3
CN
N+
O-
F
f1 (ppm)

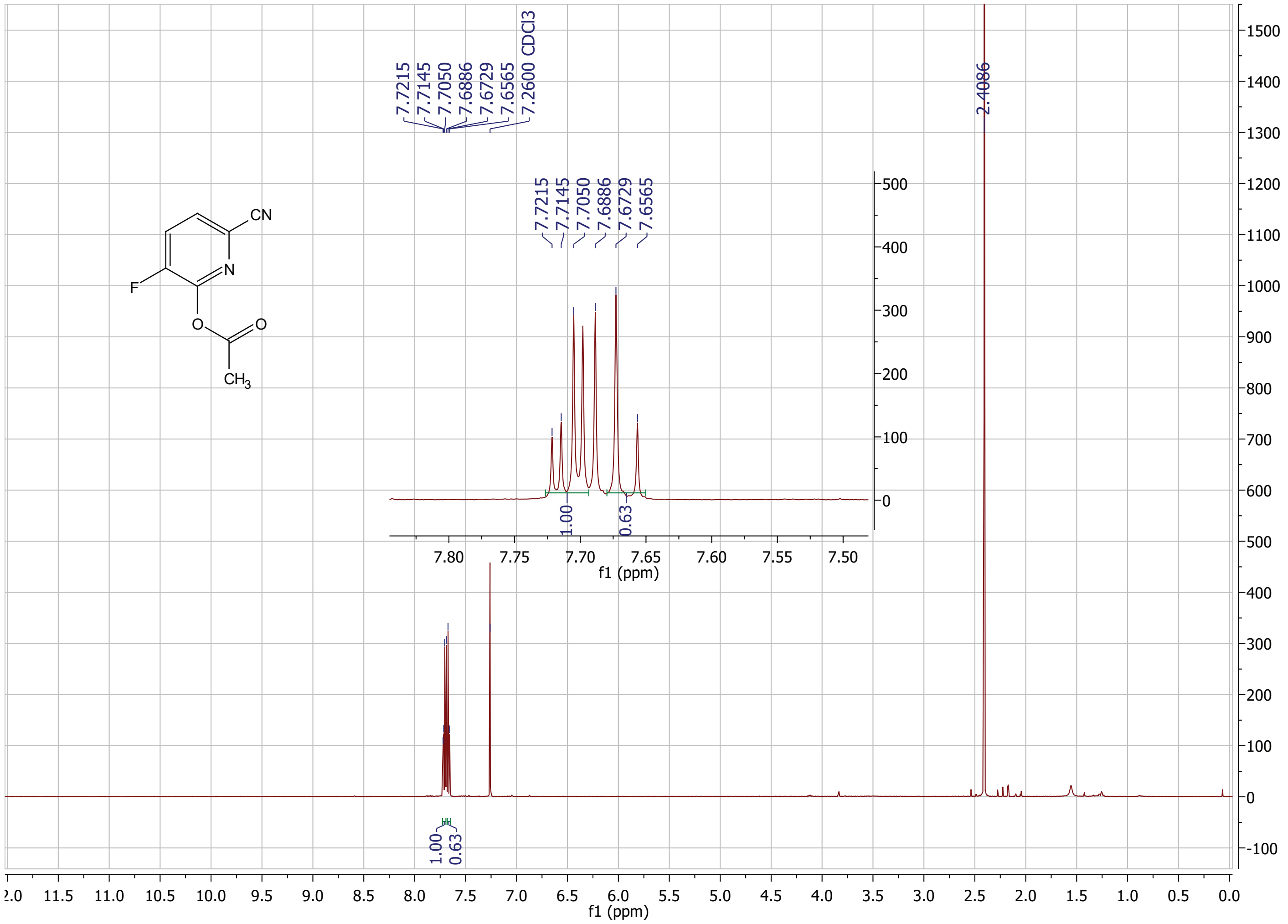
7.7215
7.7145
7.7050
7.6886
7.6729
7.6565
7.2600 CDCl3
2.4086
CN
N
F
O
O
CH3
1.00
0.63
f1 (ppm)

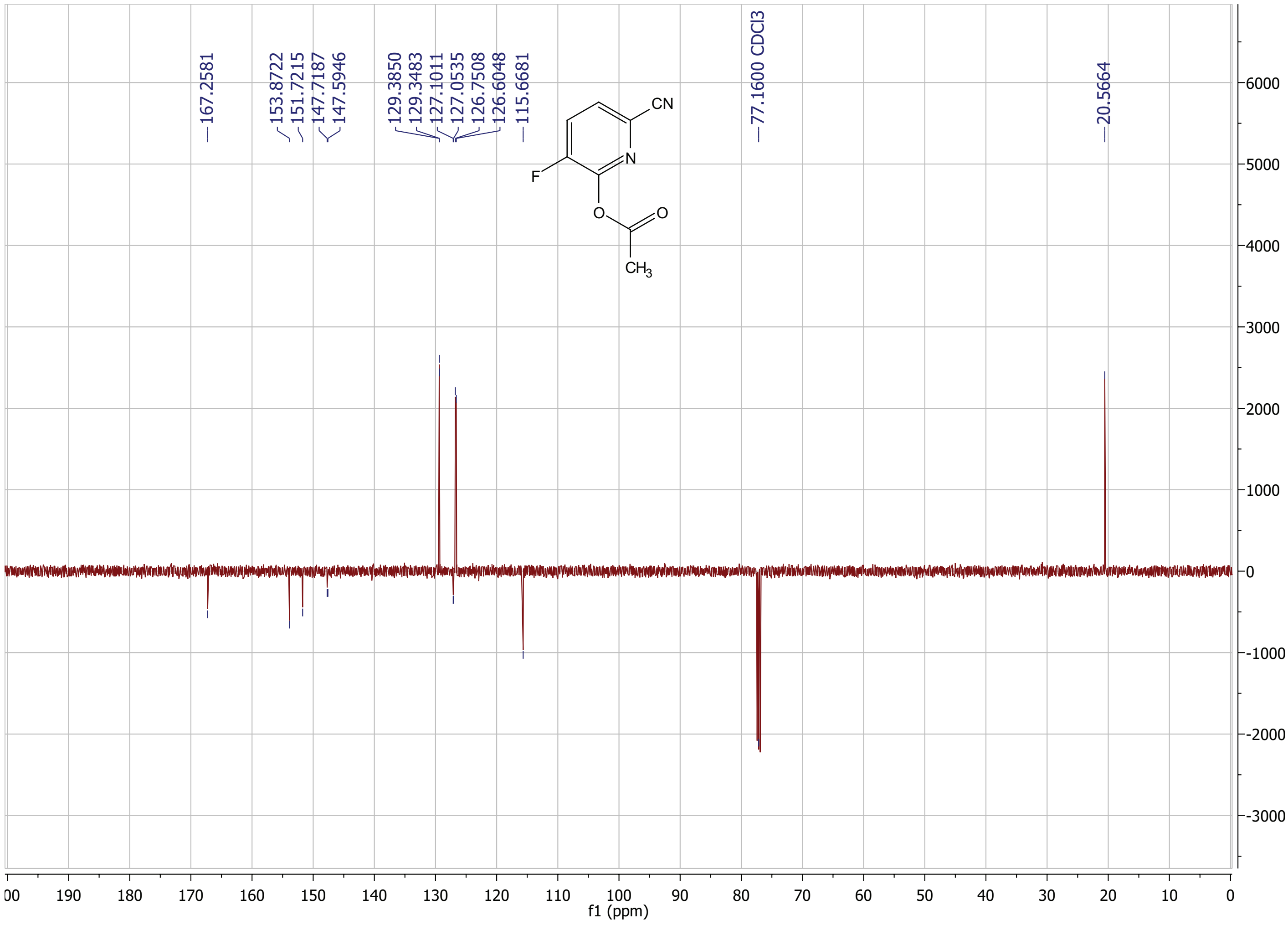
167.2581
153.8722
151.7215
147.7187
147.5946
129.3850
129.3483
127.1011
127.0535
126.7508
126.6048
115.6681
77.1600 CDCl3
20.5664
CN
N
F
O
O
CH3
f1 (ppm)

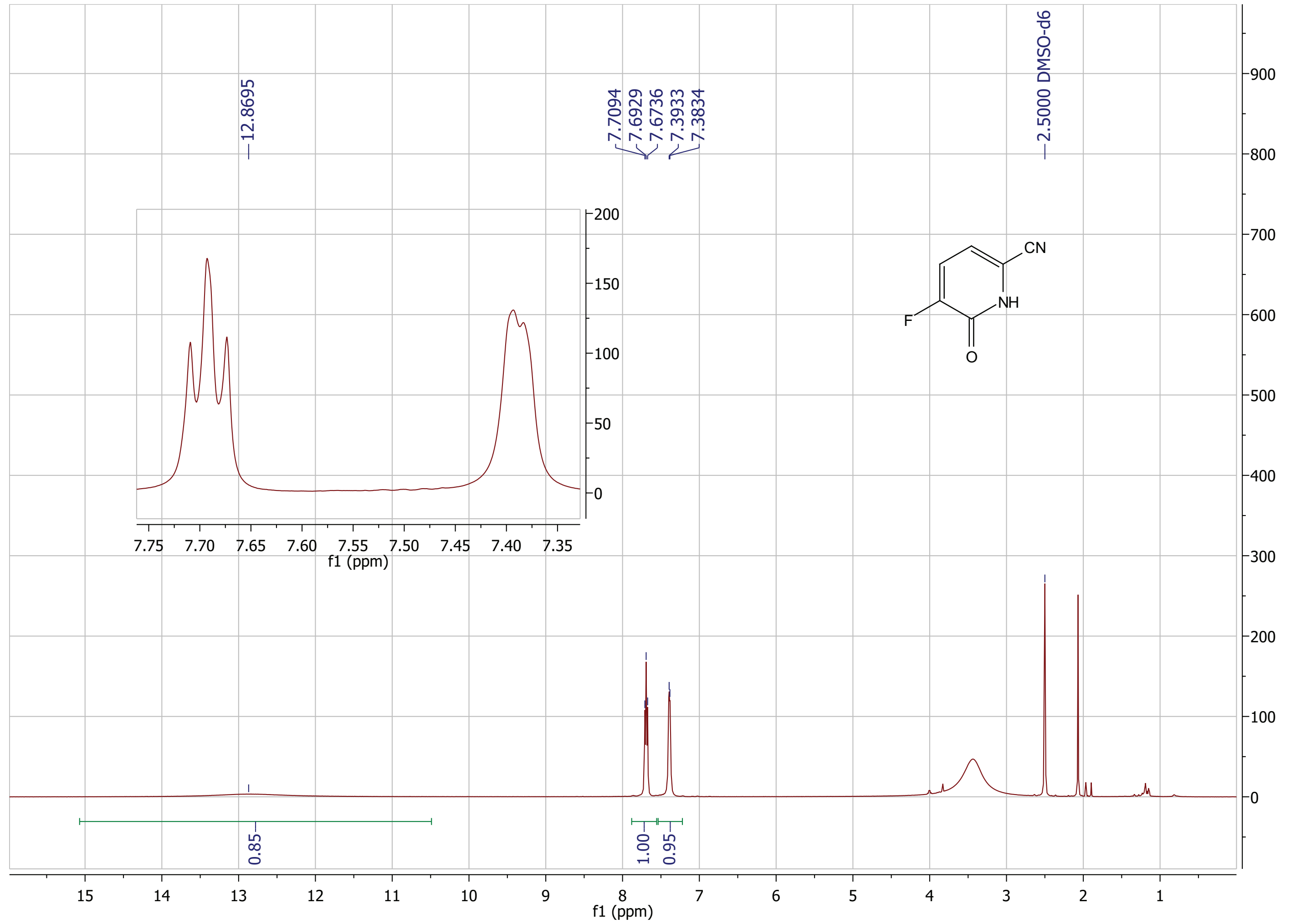

12.8695
7.7094
7.6929
7.6736
7.3933
7.3834
2.5000 DMSO-d6
CN
NH
F
O
0.85
1.00
0.95
f1 (ppm)

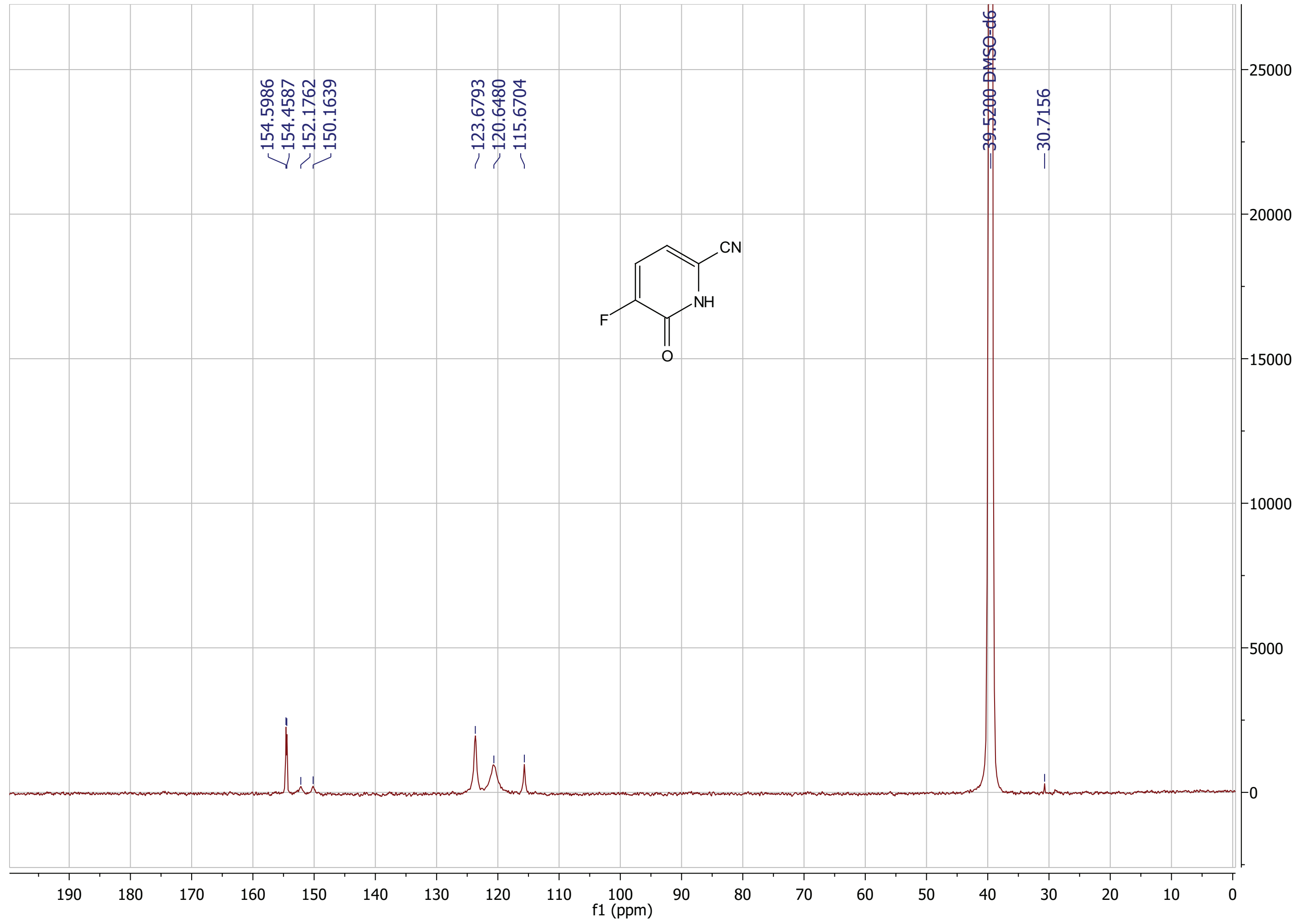

154.5986
154.4587
152.1762
150.1639
123.6793
120.6480
115.6704
39.5200 DMSO-d6
30.7156
CN
NH
F
O
f1 (ppm)

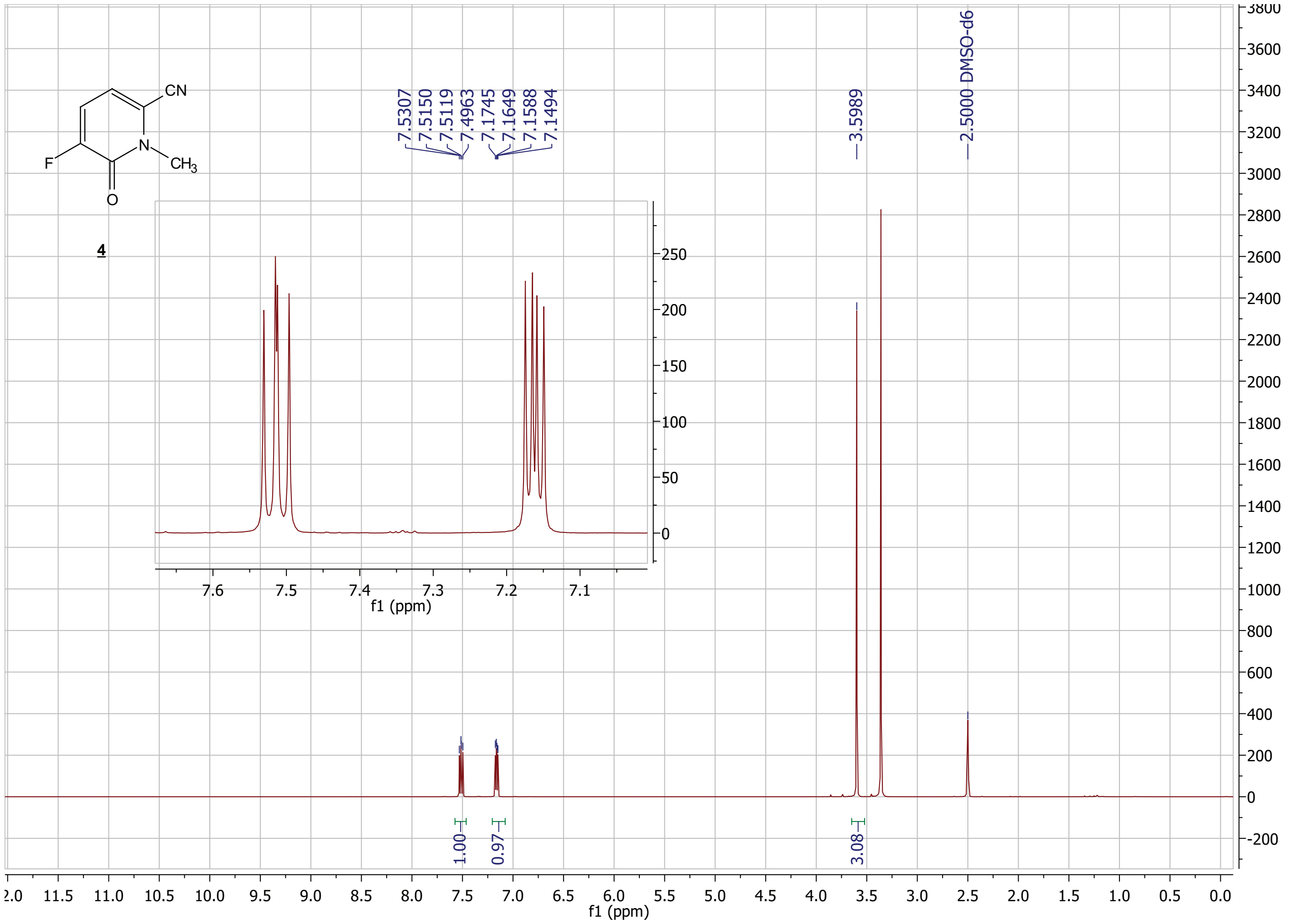
CN
F
N
CH3
O
4
7.5307
7.5150
7.5119
7.4963
7.1745
7.1649
7.1588
7.1494
3.5989
2.5000 DMSO-d6
1.00
0.97
3.08
f1 (ppm)

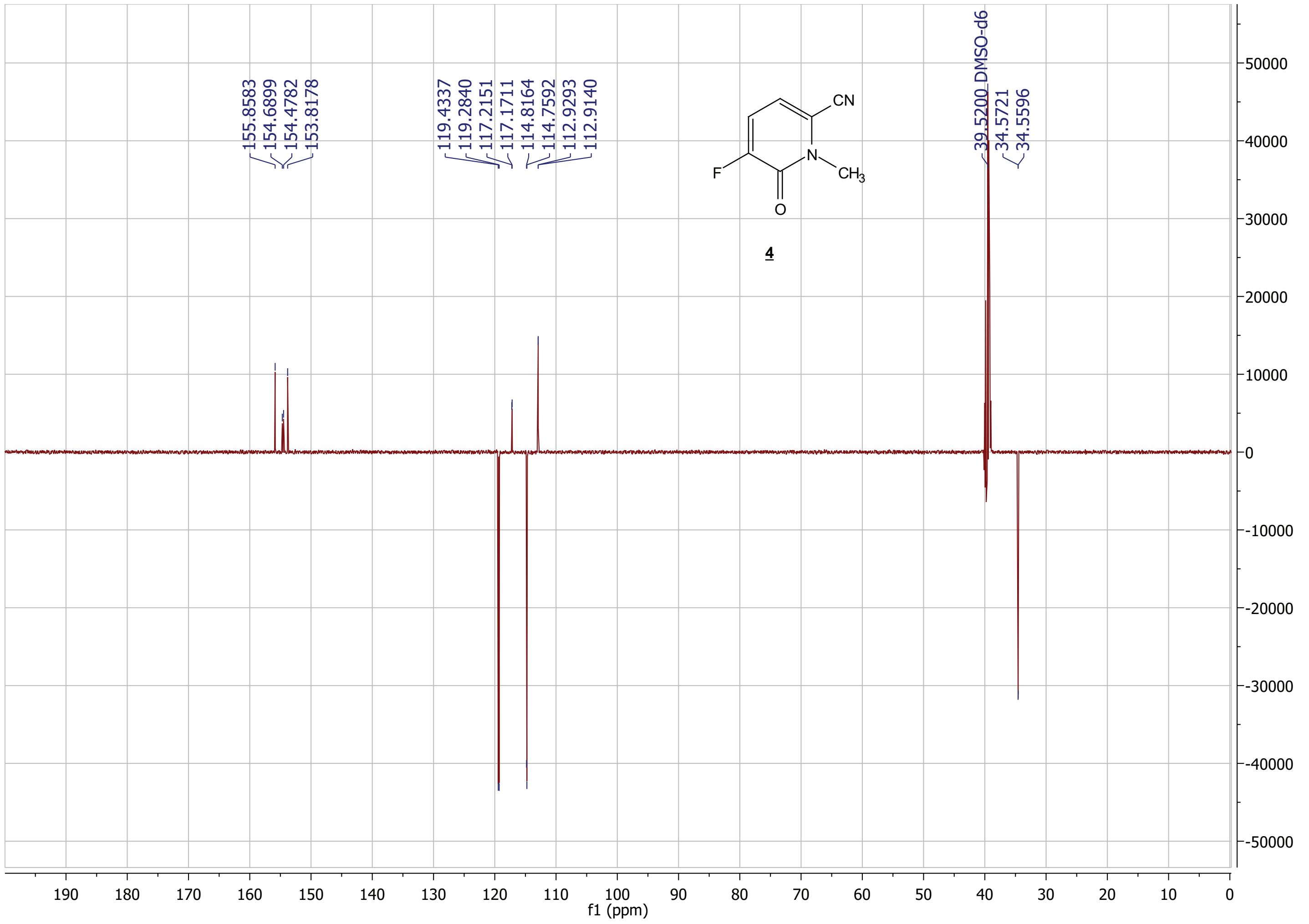
155.8583
154.6899
154.4782
153.8178
119.4337
119.2840
117.2151
117.1711
114.8164
114.7592
112.9293
112.9140
39.5200 DMSO-d6
34.5721
34.5596
CN
F
N
CH3
O
4
f1 (ppm)

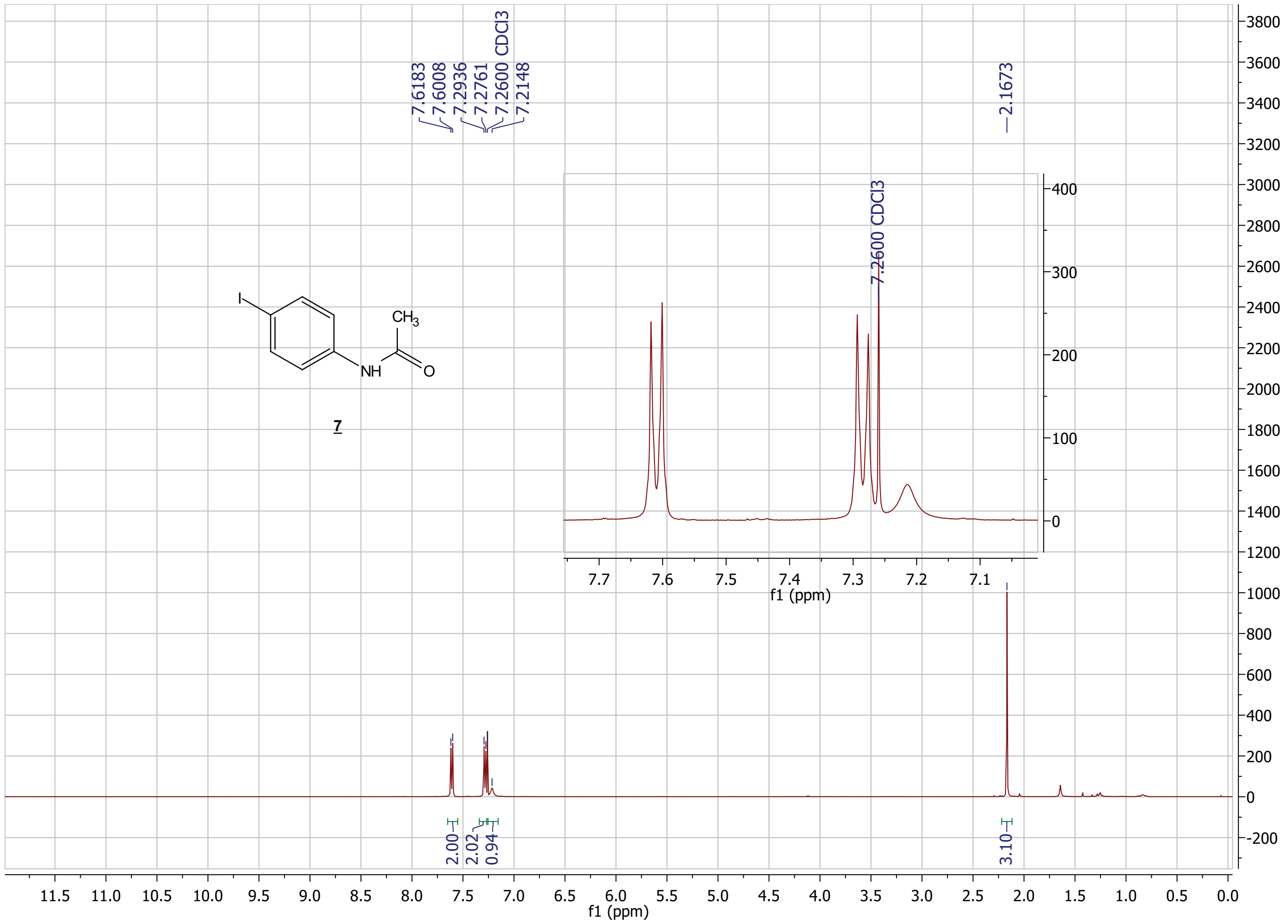
7.6183
7.6008
7.2936
7.2761
7.2600 CDCl3
7.2148
2.1673
CH3
NH
O
I
7
2.00
2.02
0.94
3.10
f1 (ppm)

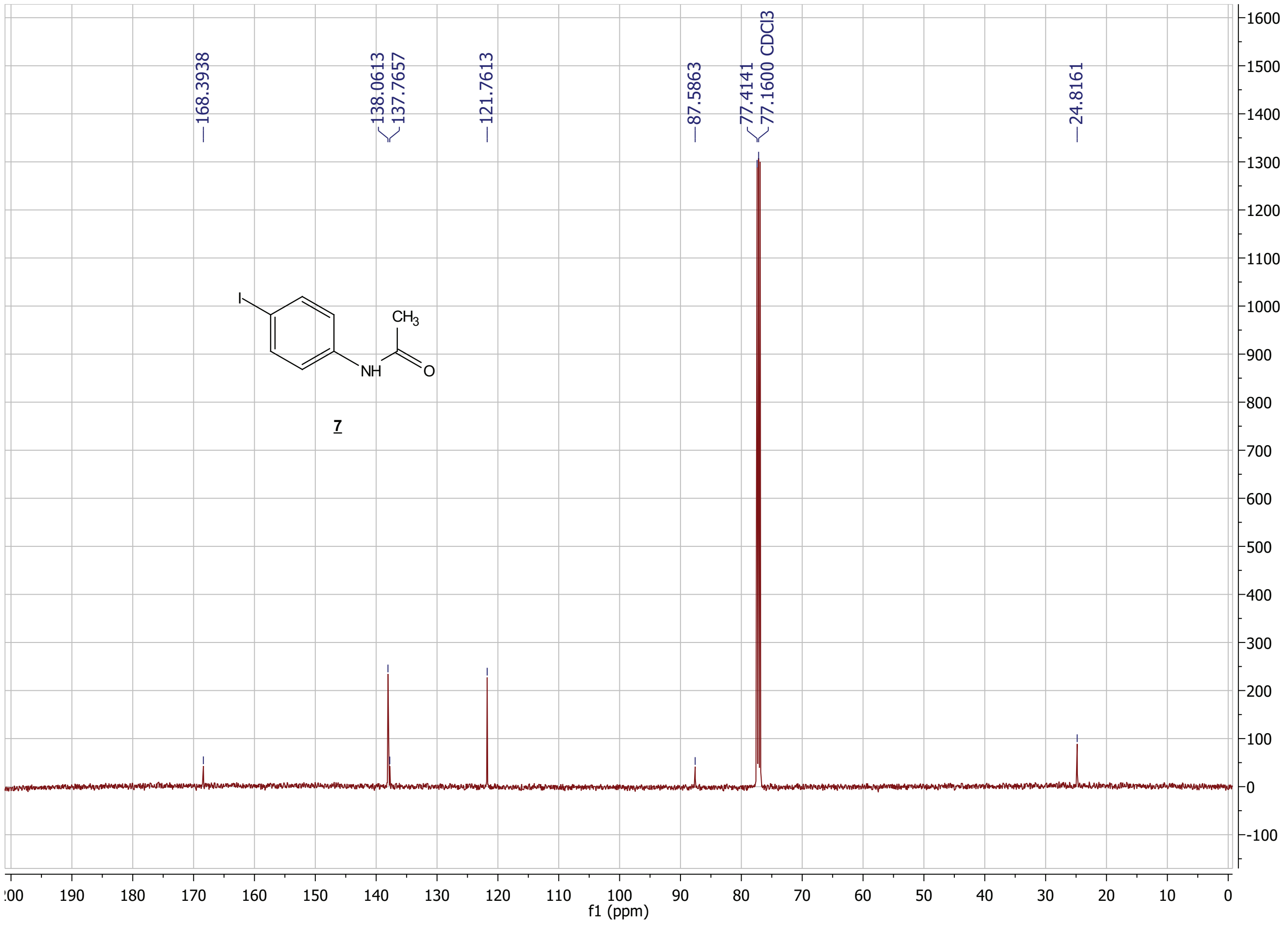
168.3938
138.0613
137.7657
121.7613
87.5863
77.4141
77.1600 CDCl3
24.8161
CH3
NH
O
I
7
f1 (ppm)

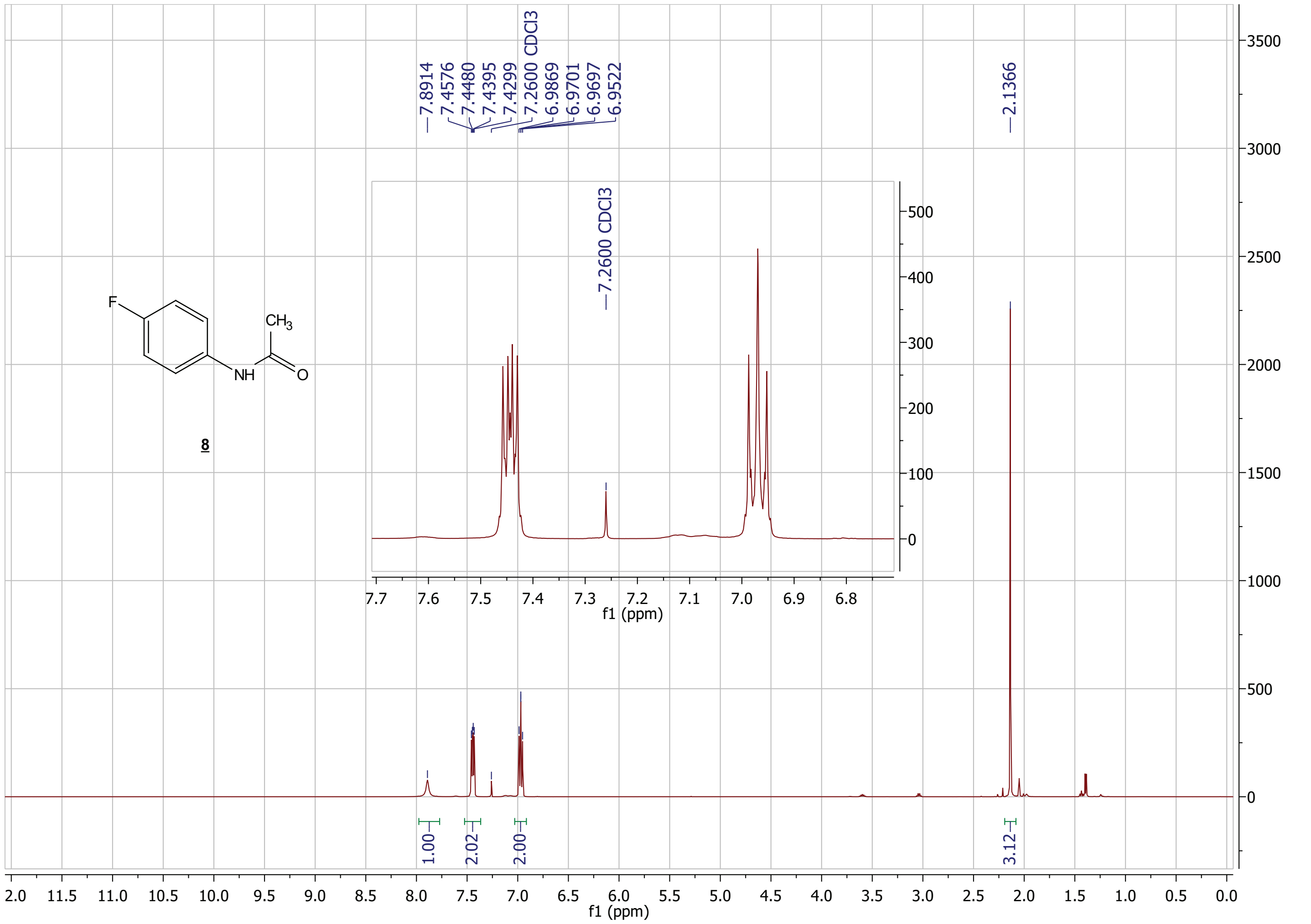

7.8914
7.4576
7.4480
7.4395
7.4299
7.2600 CDCl3
6.9869
6.9701
6.9697
6.9522
2.1366
1.00
2.02
2.00
3.12
8
f1 (ppm)

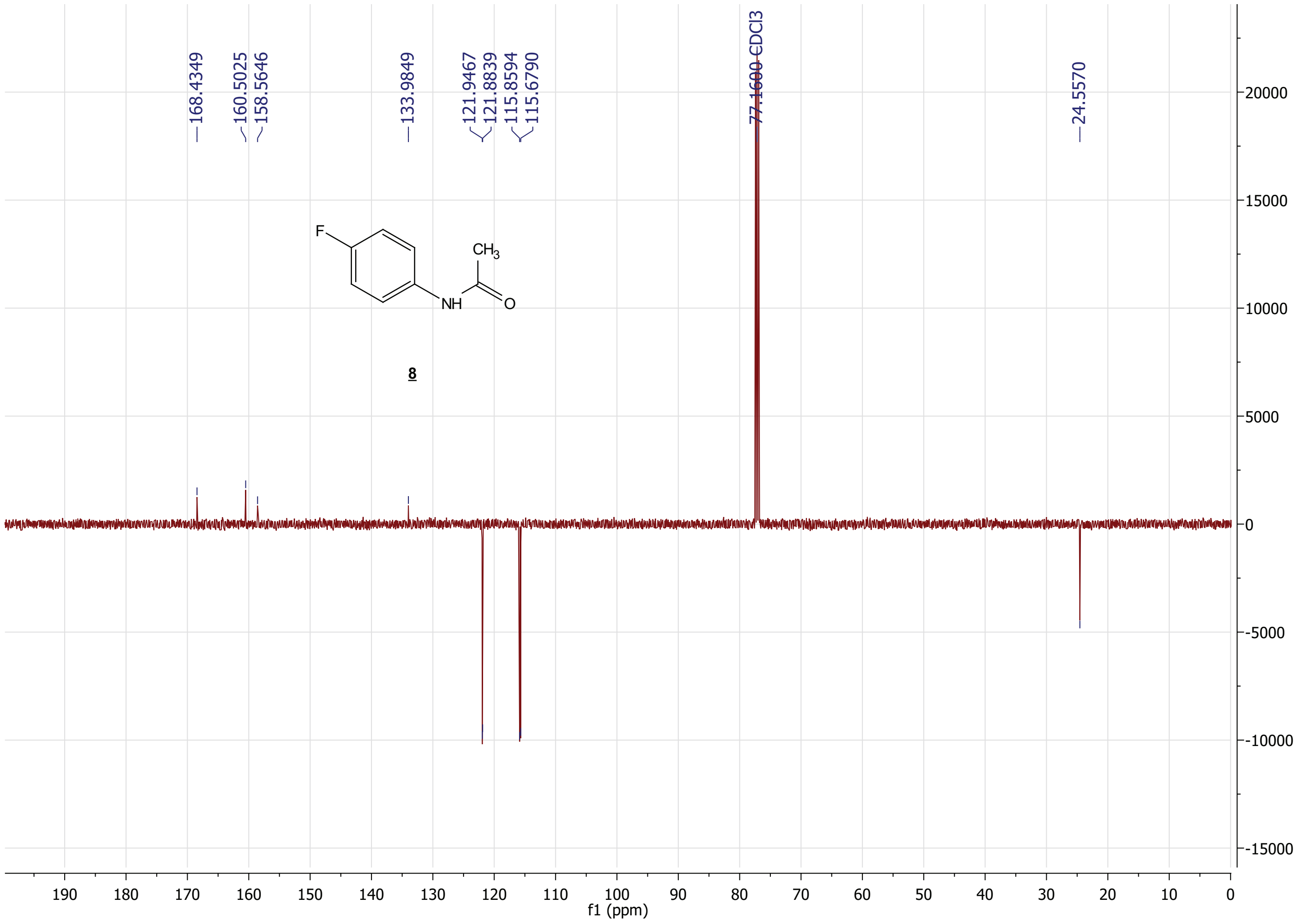

168.4349
160.5025
158.5646
133.9849
121.9467
121.8839
115.8594
115.6790
77.1600 CDCl3
24.5570
8
f1 (ppm)

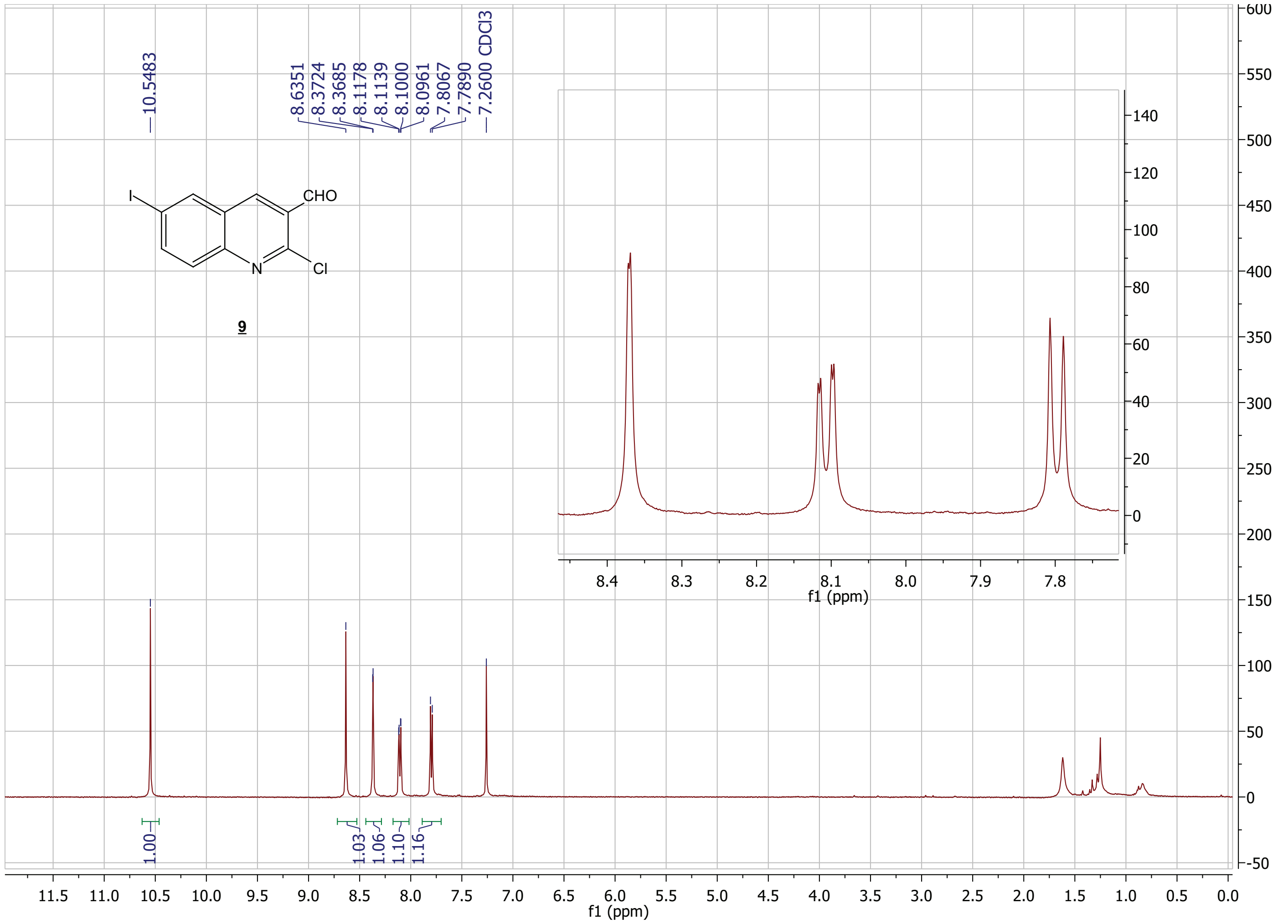
10.5483
8.6351
8.3724
8.3685
8.1178
8.1139
8.1000
8.0961
7.8067
7.7890
7.2600 CDCl3
CHO
I
N
Cl
9
1.00
1.03
1.06
1.10
1.16
f1 (ppm)

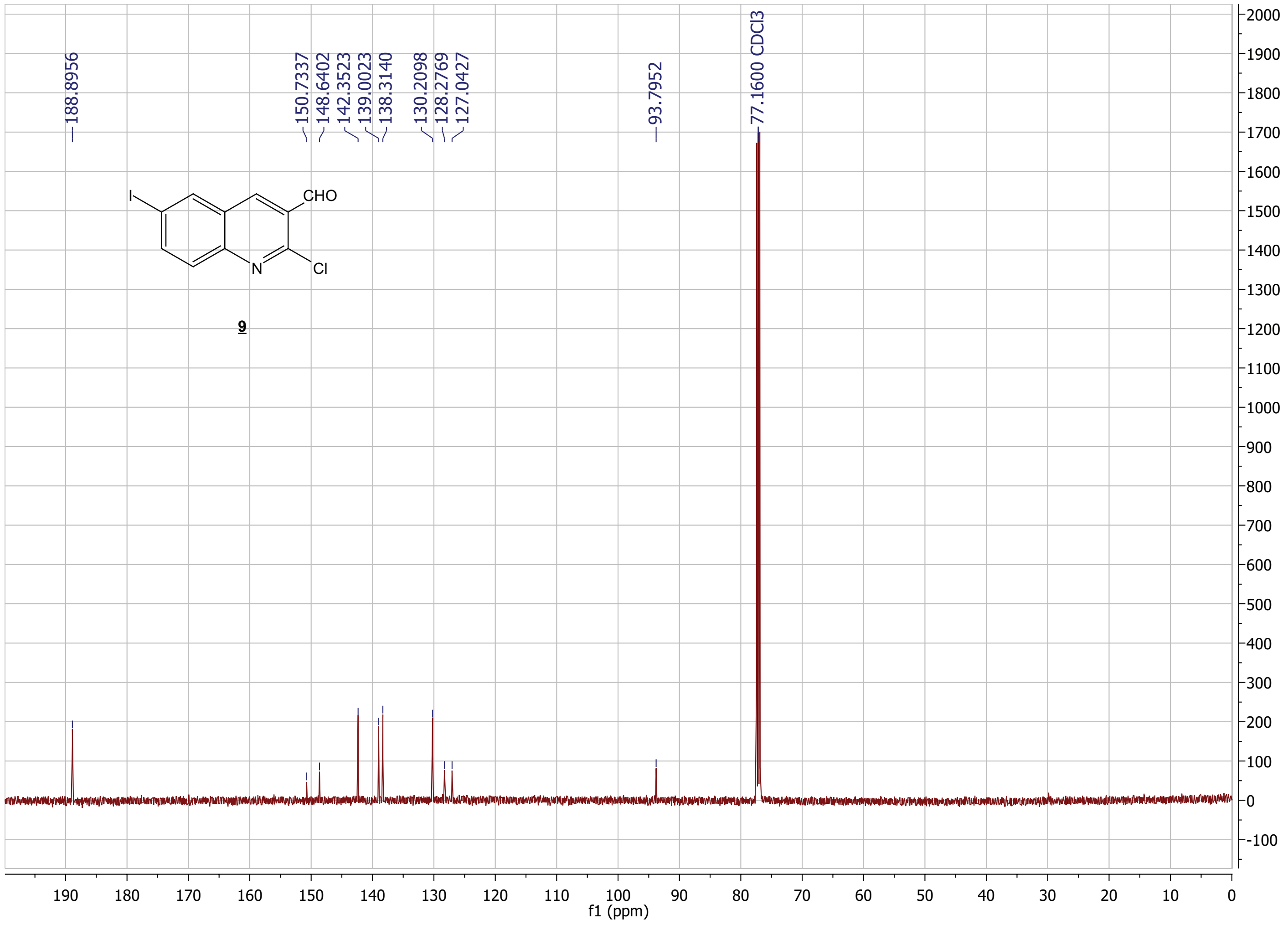
188.8956
150.7337
148.6402
142.3523
139.0023
138.3140
130.2098
128.2769
127.0427
93.7952
77.1600 CDCl3
CHO
I
N
Cl
9
f1 (ppm)

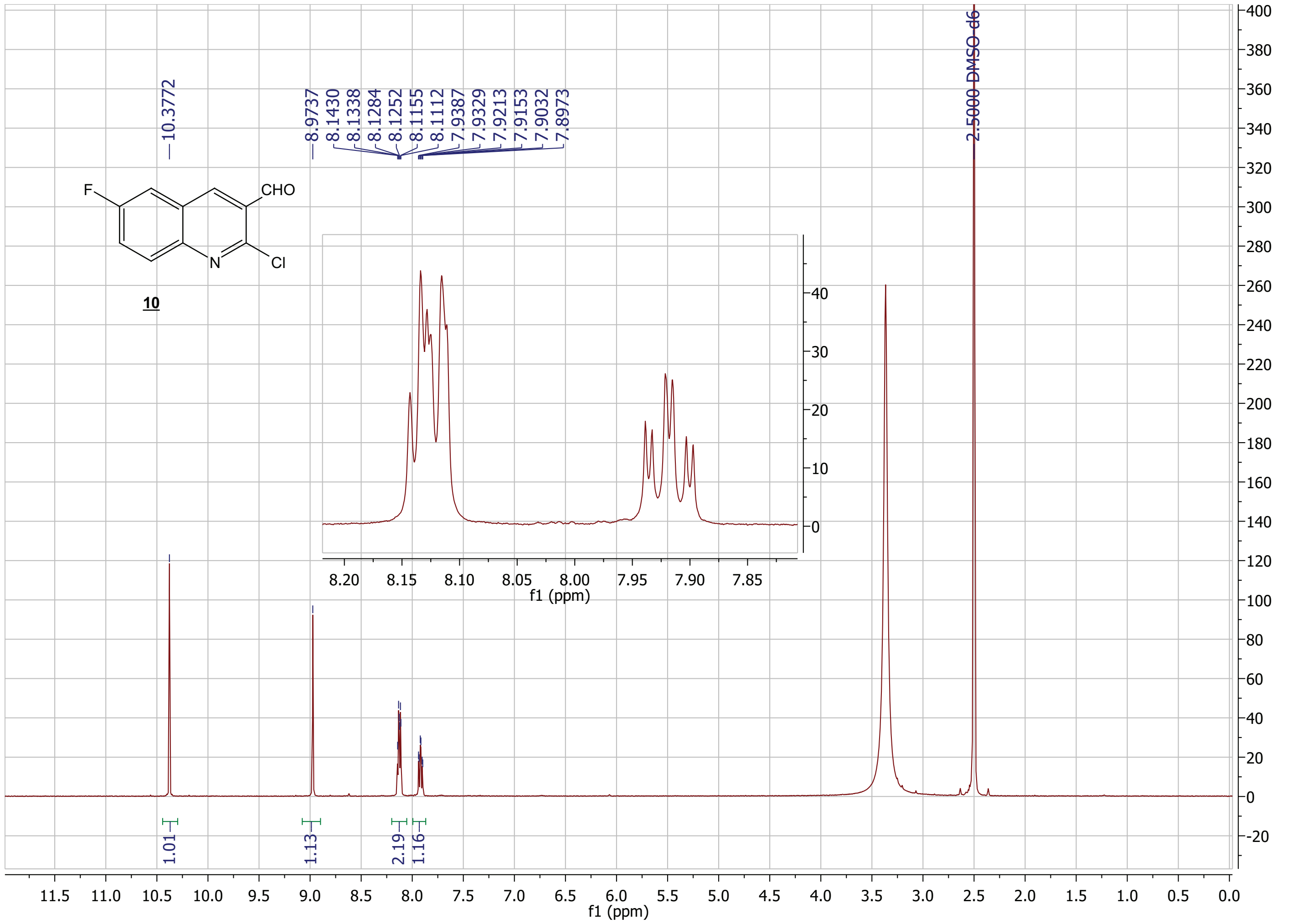
10.3772
8.9737
8.1430
8.1338
8.1284
8.1252
8.1155
8.1112
7.9387
7.9329
7.9213
7.9153
7.9032
7.8973
2.5000 DMSO d6
F
CHO
N
Cl
10
1.01
1.13
2.19
1.16
f1 (ppm)

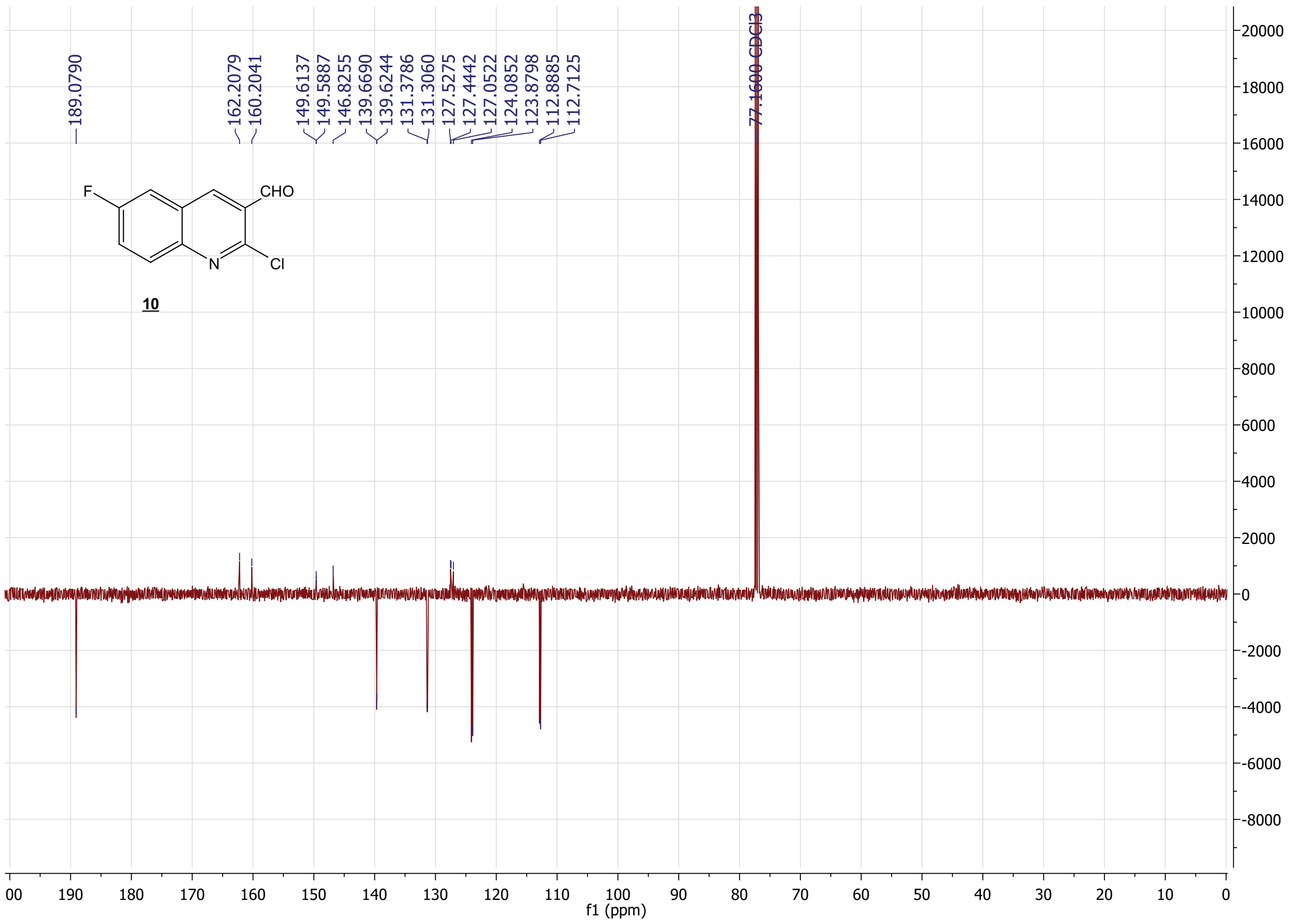
189.0790
162.2079
160.2041
149.6137
149.5887
146.8255
139.6690
139.6244
131.3786
131.3060
127.5275
127.4442
127.0522
124.0852
123.8798
112.8885
112.7125
77.1600 CDCl3
F
CHO
N
Cl
10
f1 (ppm)

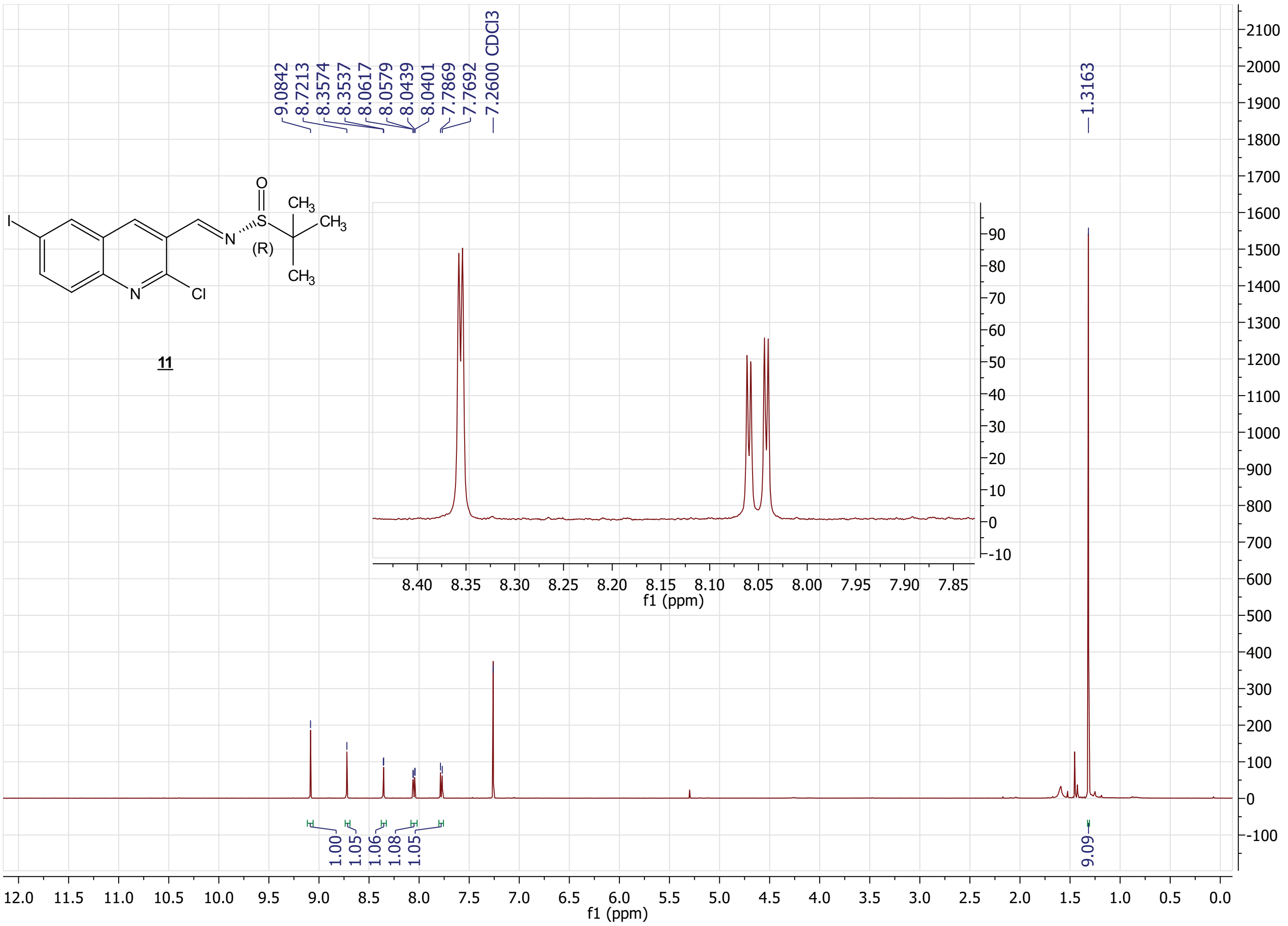

9.0842
8.7213
8.3574
8.3537
8.0617
8.0579
8.0439
8.0401
7.7869
7.7692
7.2600 CDCl3
1.3163
1.00
1.05
1.06
1.08
1.05
9.09
f1 (ppm)

11

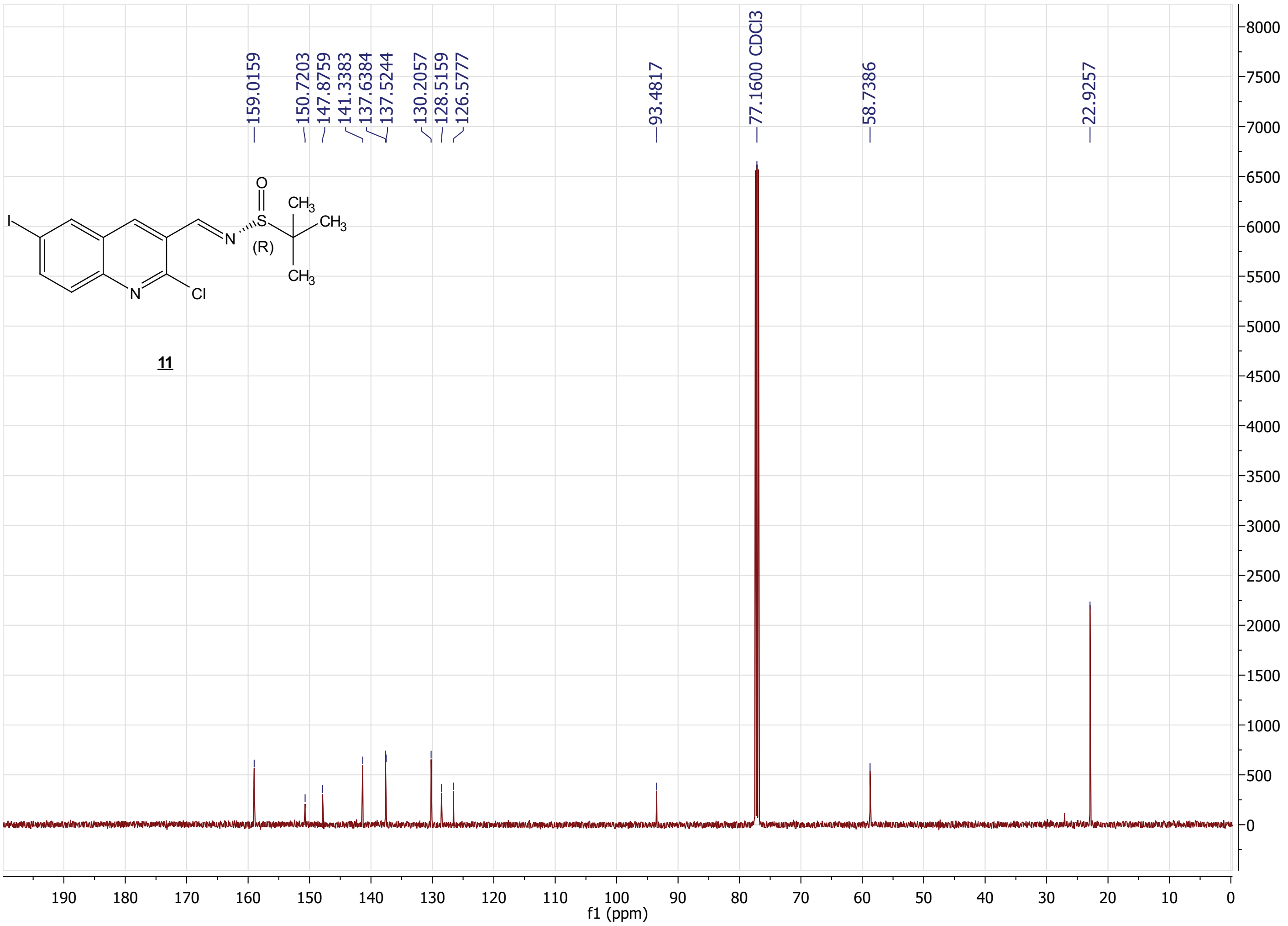

159.0159
150.7203
147.8759
141.3383
137.6384
137.5244
130.2057
128.5159
126.5777
93.4817
77.1600 CDCl3
58.7386
22.9257
f1 (ppm)

11

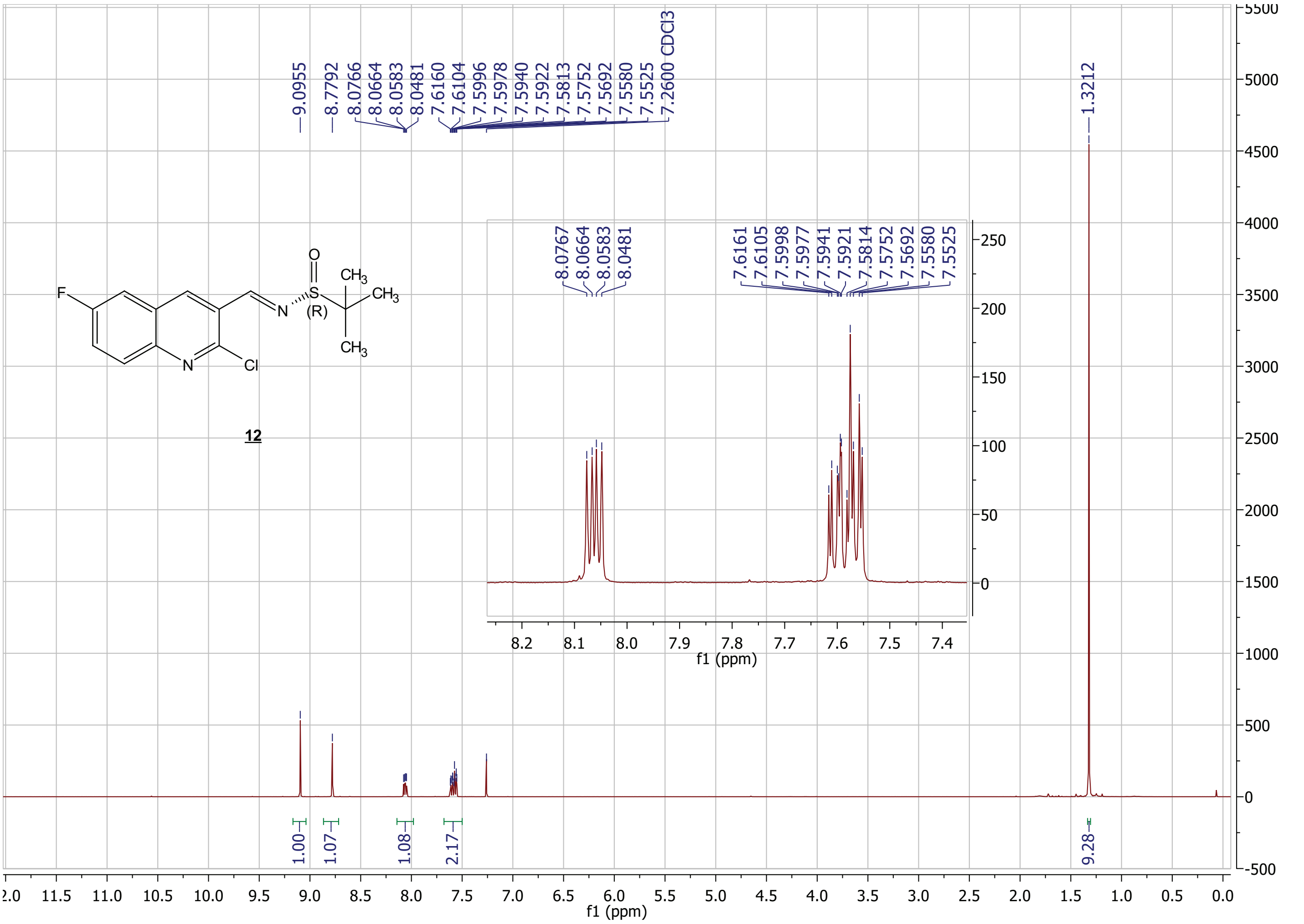
9.0955
8.7792
8.0766
8.0664
8.0583
8.0481
7.6160
7.6104
7.5996
7.5978
7.5940
7.5922
7.5813
7.5752
7.5692
7.5580
7.5525
7.2600 CDCl3
1.3212
1.00
1.07
1.08
2.17
9.28
f1 (ppm)
12

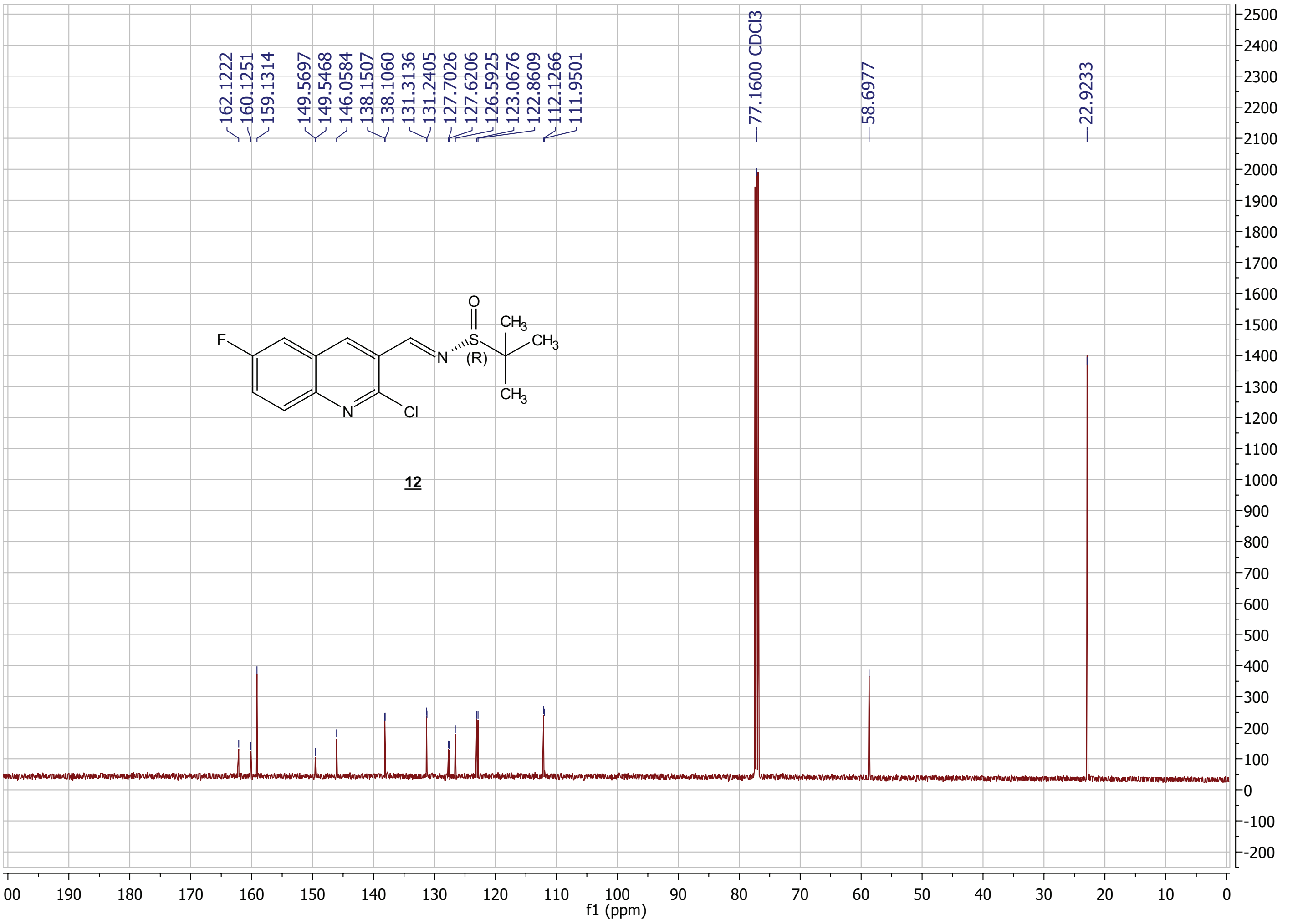
162.1222
160.1251
159.1314
149.5697
149.5468
146.0584
138.1507
138.1060
131.3136
131.2405
127.7026
127.6206
126.5925
123.0676
122.8609
112.1266
111.9501
77.1600 CDCl3
58.6977
22.9233
f1 (ppm)
12

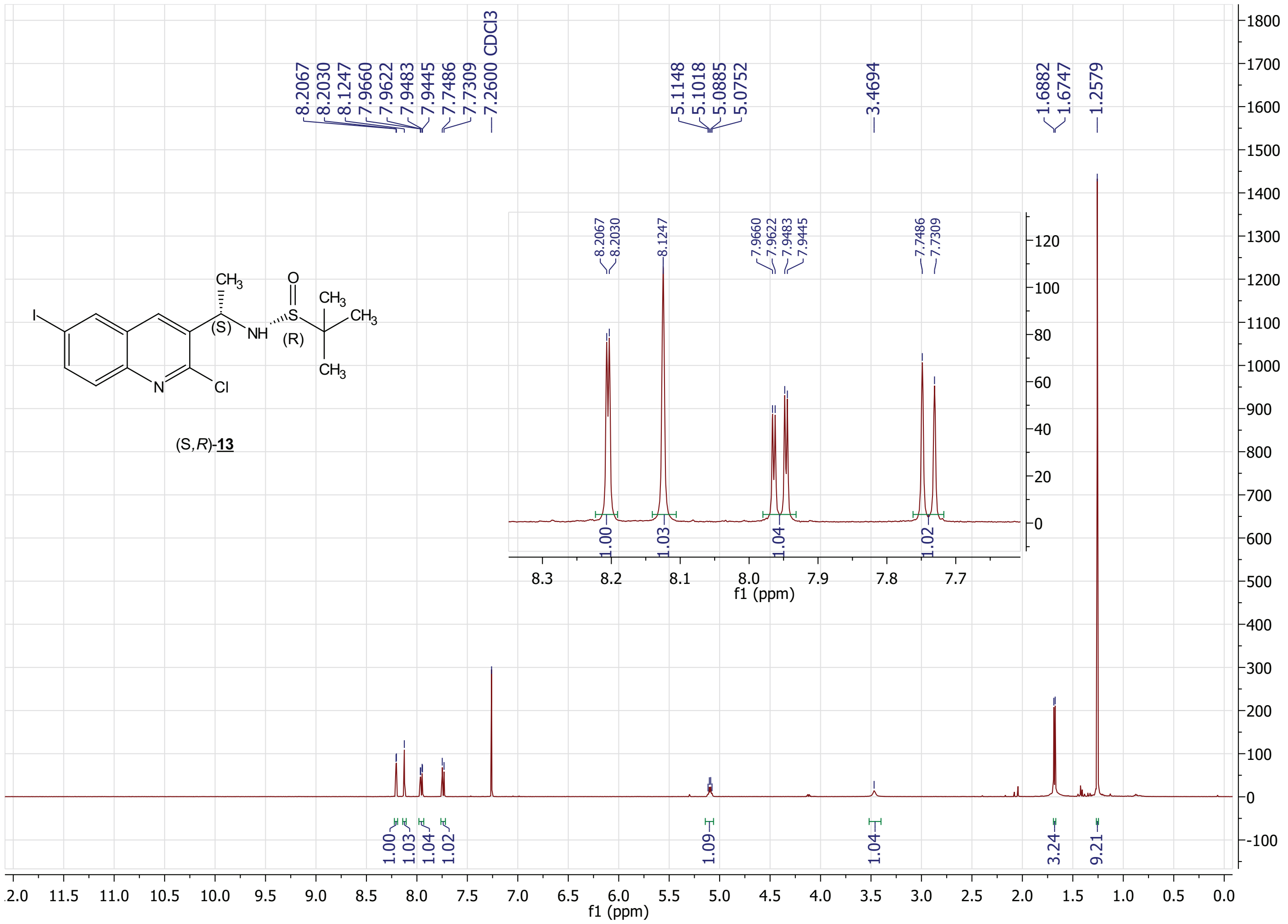
8.2067
8.2030
8.1247
7.9660
7.9622
7.9483
7.9445
7.7486
7.7309
7.2600 CDCl3
5.1148
5.1018
5.0885
5.0752
3.4694
1.6882
1.6747
1.2579
(S,R)-13
1.00
1.03
1.04
1.02
1.09
1.04
3.24
9.21
f1 (ppm)

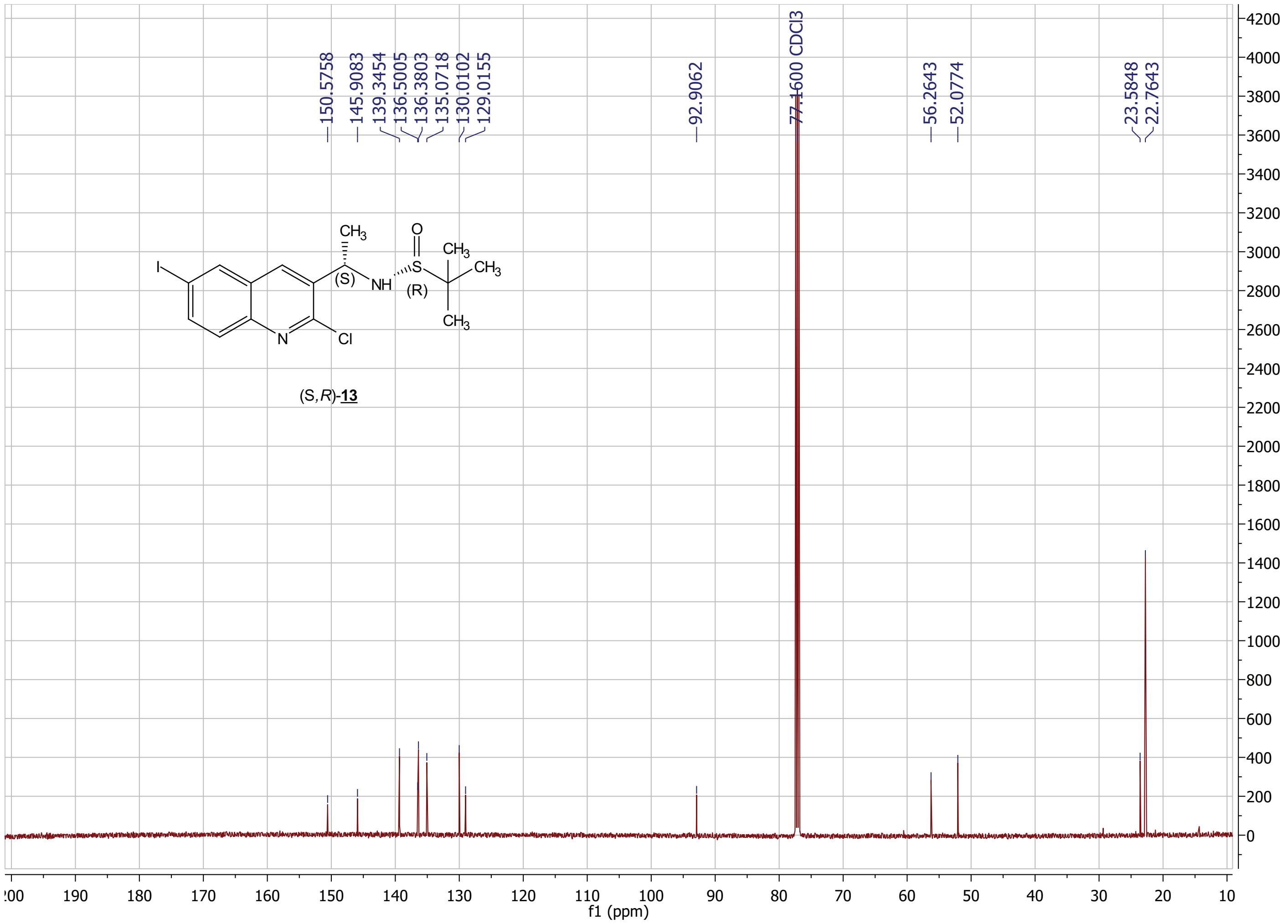
150.5758
145.9083
139.3454
136.5005
136.3803
135.0718
130.0102
129.0155
92.9062
77.1600 CDCl3
56.2643
52.0774
23.5848
22.7643
(S,R)-13
f1 (ppm)

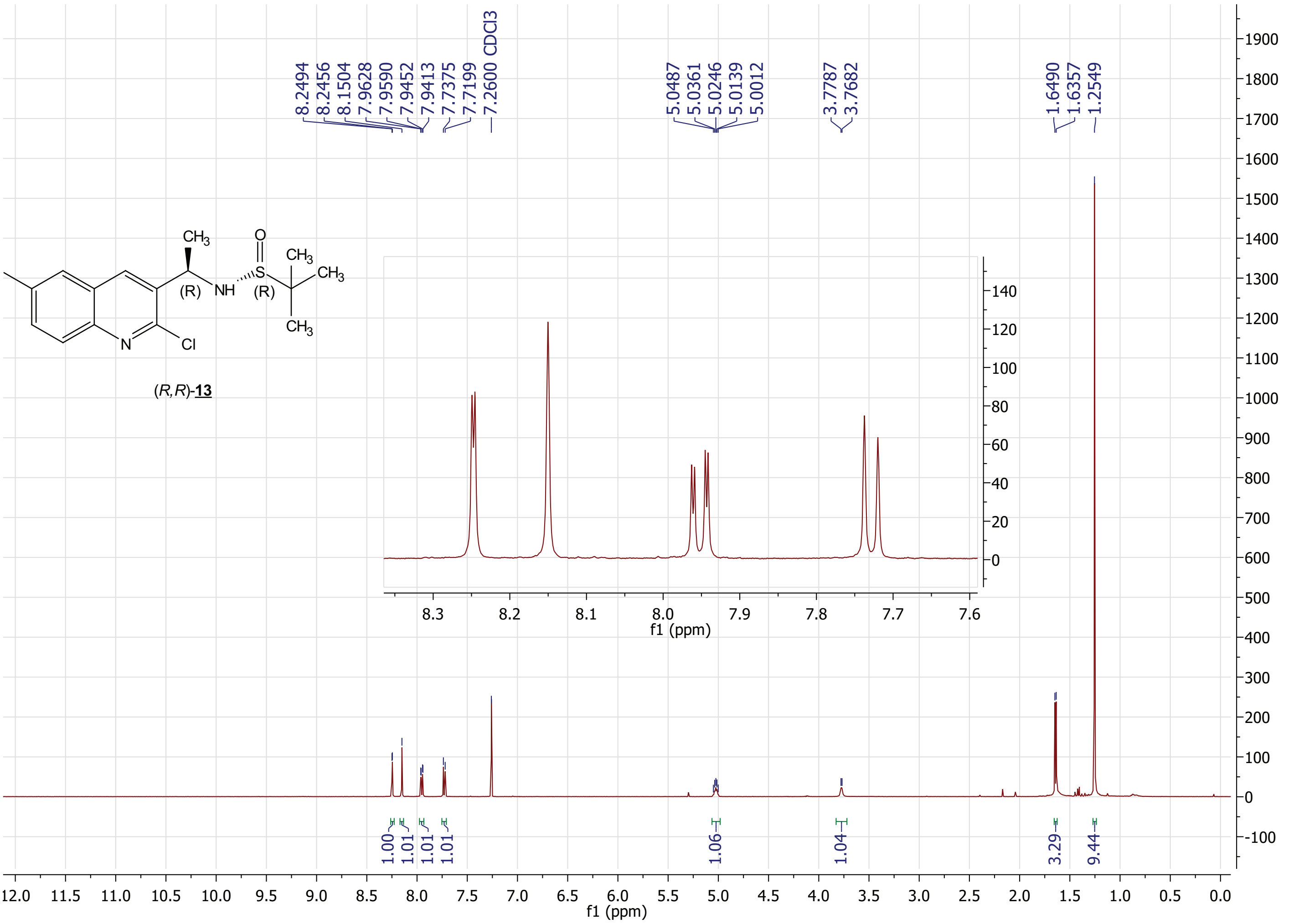
8.2494
8.2456
8.1504
7.9628
7.9590
7.9452
7.9413
7.7375
7.7199
7.2600 CDCl3
5.0487
5.0361
5.0246
5.0139
5.0012
3.7787
3.7682
1.6490
1.6357
1.2549
CH3
O
S
CH3
CH3
(R)
NH
(R)
CH3
N
Cl
(R,R)-13
1.00
1.01
1.01
1.01
1.06
1.04
3.29
9.44
f1 (ppm)

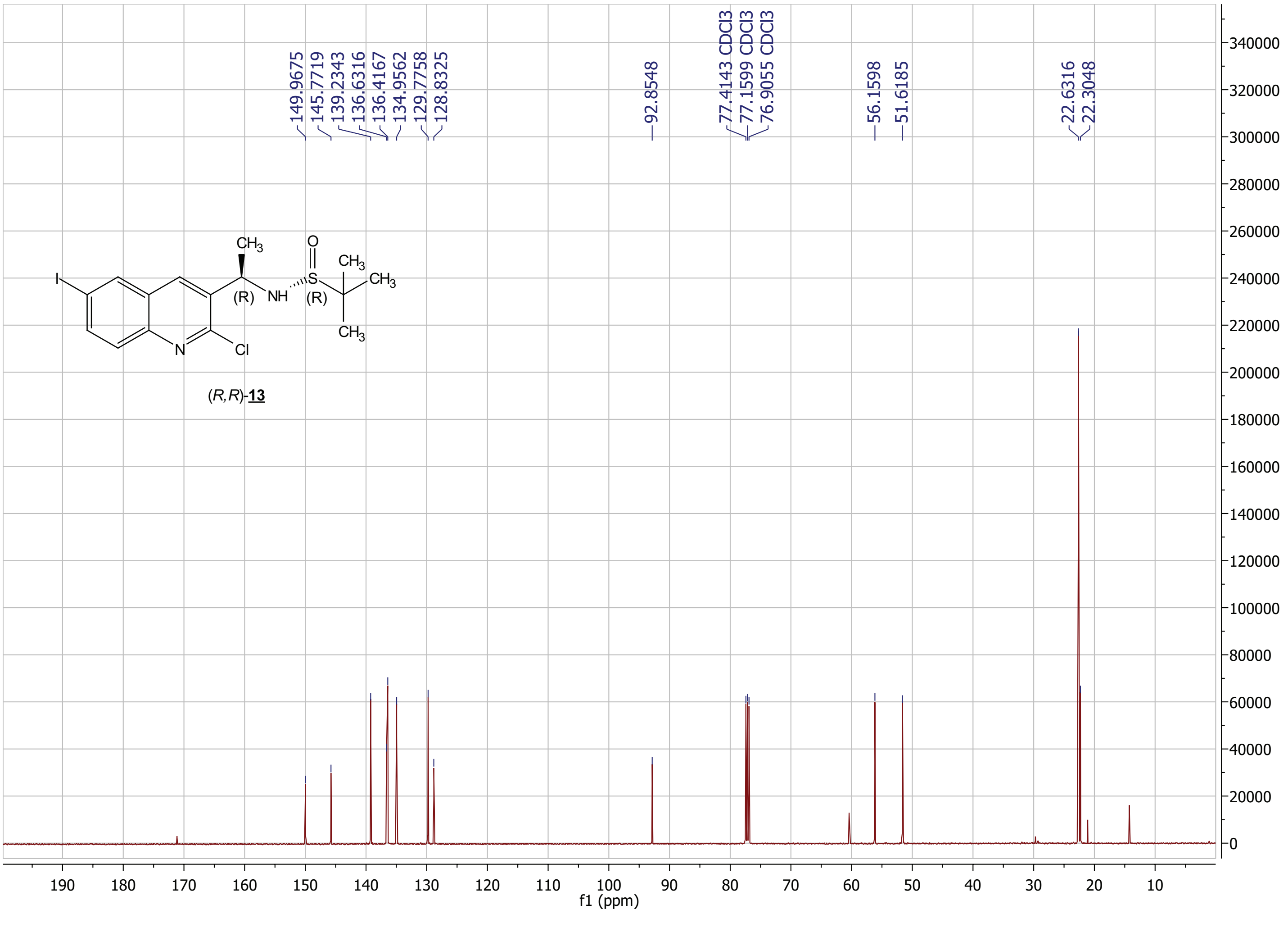
149.9675
145.7719
139.2343
136.6316
136.4167
134.9562
129.7758
128.8325
92.8548
77.4143 CDCl3
77.1599 CDCl3
76.9055 CDCl3
56.1598
51.6185
22.6316
22.3048
I
CH3
O
CH3
CH3
(R)
NH
(R)
S
CH3
N
Cl
(R,R)-13
f1 (ppm)

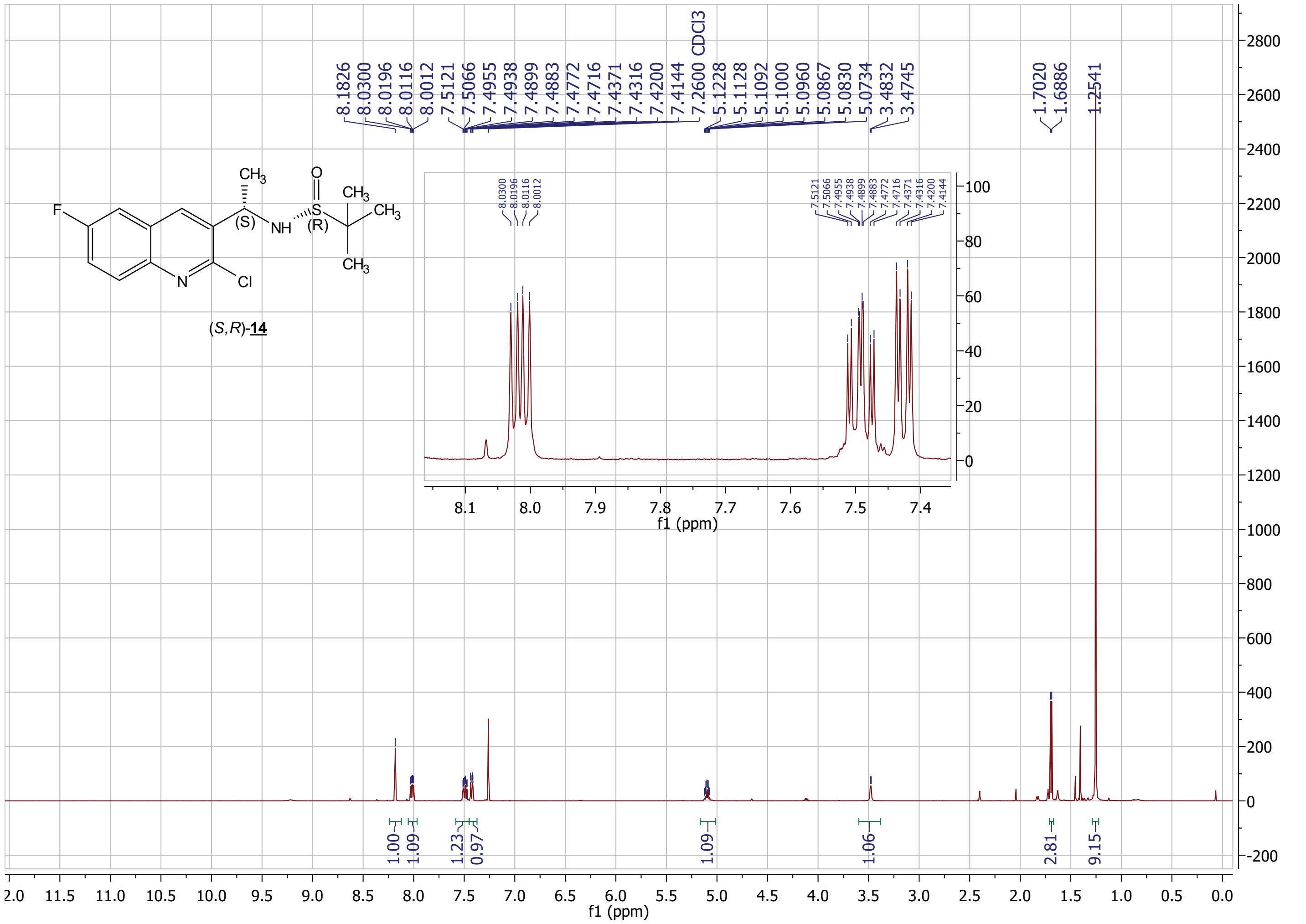
8.1826
8.0300
8.0196
8.0116
8.0012
7.5121
7.5066
7.4955
7.4938
7.4899
7.4883
7.4772
7.4716
7.4371
7.4316
7.4200
7.4144
7.2600 CDCl3
5.1228
5.1128
5.1092
5.1000
5.0960
5.0867
5.0830
5.0734
3.4832
3.4745
1.7020
1.6886
1.2541
(S,R)-14
1.00
1.09
1.23
0.97
1.09
1.06
2.81
9.15
f1 (ppm)

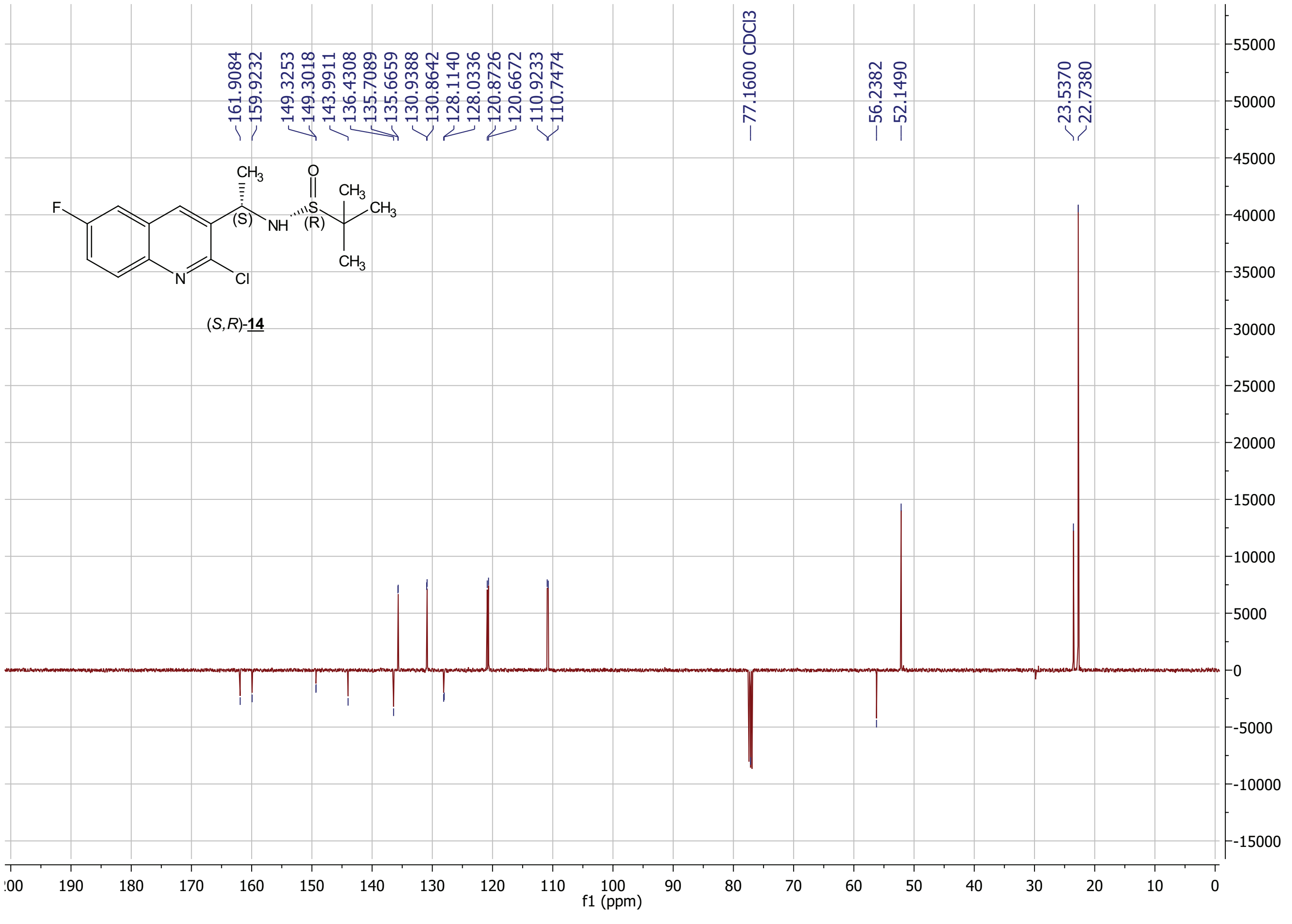
161.9084
159.9232
149.3253
149.3018
143.9911
136.4308
135.7089
135.6659
130.9388
130.8642
128.1140
128.0336
120.8726
120.6672
110.9233
110.7474
77.1600 CDCl3
56.2382
52.1490
23.5370
22.7380
(S,R)-14
f1 (ppm)

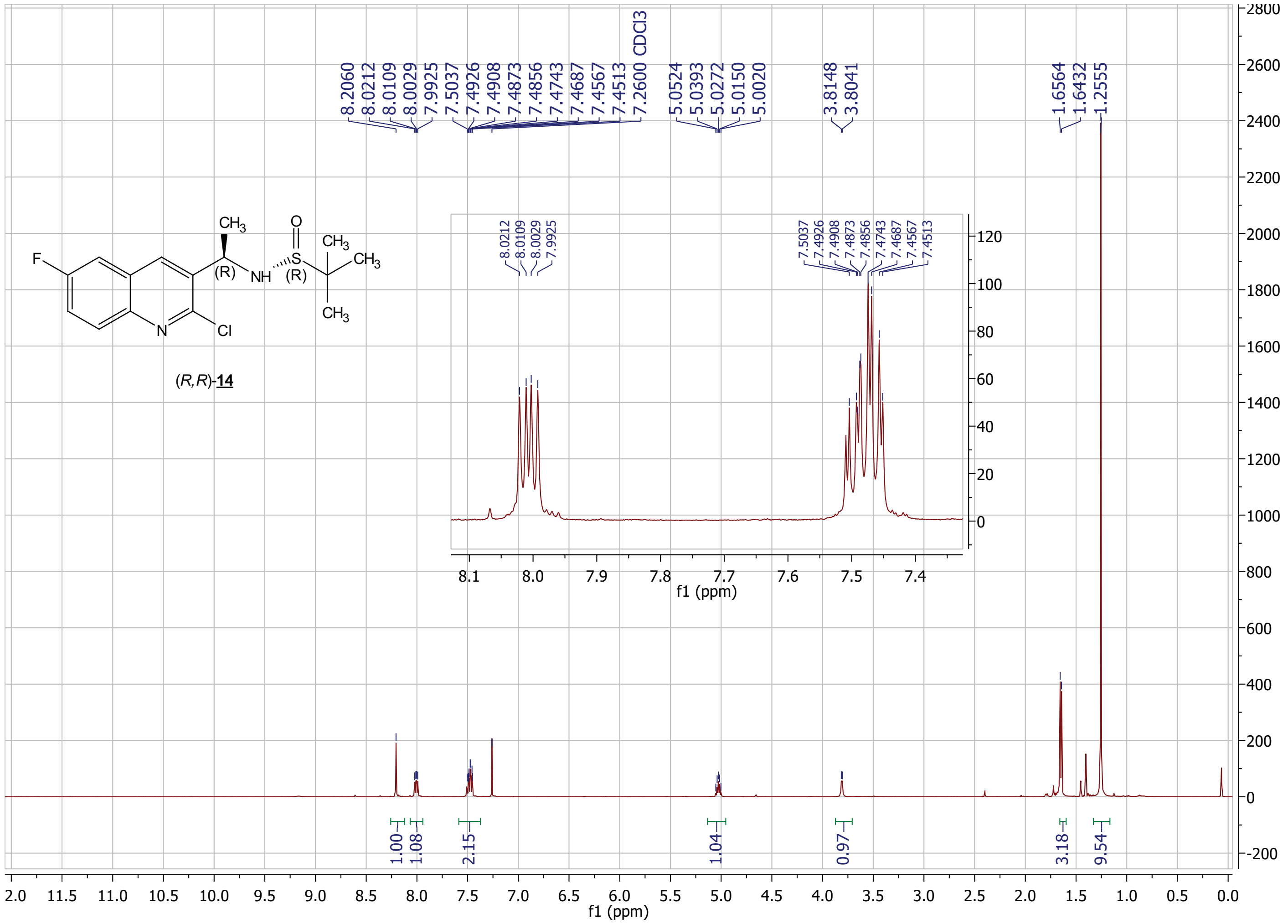

8.2060
8.0212
8.0109
8.0029
7.9925
7.5037
7.4926
7.4908
7.4873
7.4856
7.4743
7.4687
7.4567
7.4513
7.2600 CDCl3
5.0524
5.0393
5.0272
5.0150
5.0020
3.8148
3.8041
1.6564
1.6432
1.2555
(R,R)-14
1.00
1.08
2.15
1.04
0.97
3.18
9.54
f1 (ppm)

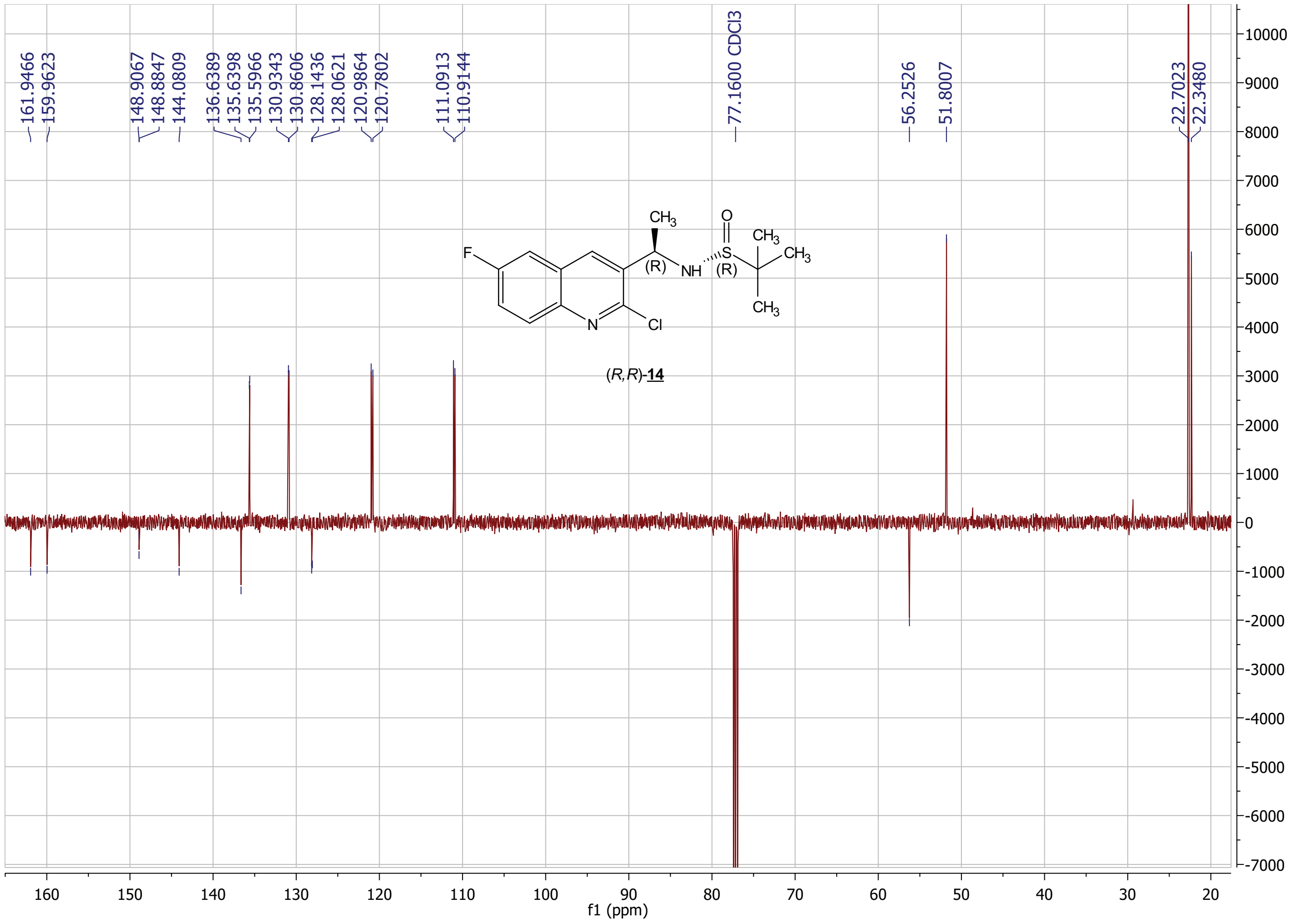

161.9466
159.9623
148.9067
148.8847
144.0809
136.6389
135.6398
135.5966
130.9343
130.8606
128.1436
128.0621
120.9864
120.7802
111.0913
110.9144
77.1600 CDCl3
56.2526
51.8007
22.7023
22.3480
(R,R)-14
f1 (ppm)

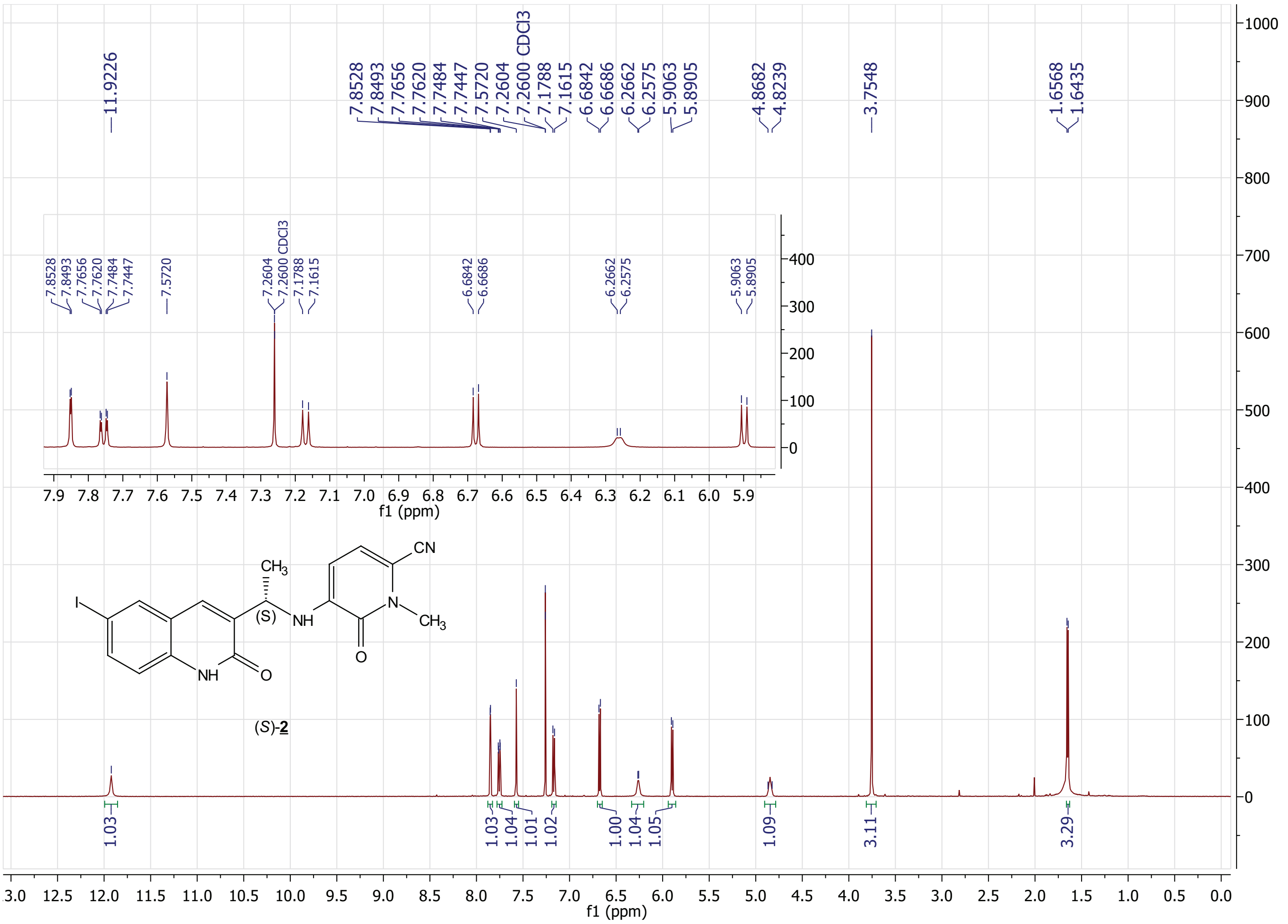
11.9226
7.8528
7.8493
7.7656
7.7620
7.7484
7.7447
7.5720
7.2604
7.2600 CDCl3
7.1788
7.1615
6.6842
6.6686
6.2662
6.2575
5.9063
5.8905
4.8682
4.8239
3.7548
1.6568
1.6435
1.03
1.03
1.04
1.01
1.02
1.00
1.04
1.05
1.09
3.11
3.29
CH3
CN
I
(S)
NH
N
CH3
NH
O
O
(S)-2
f1 (ppm)

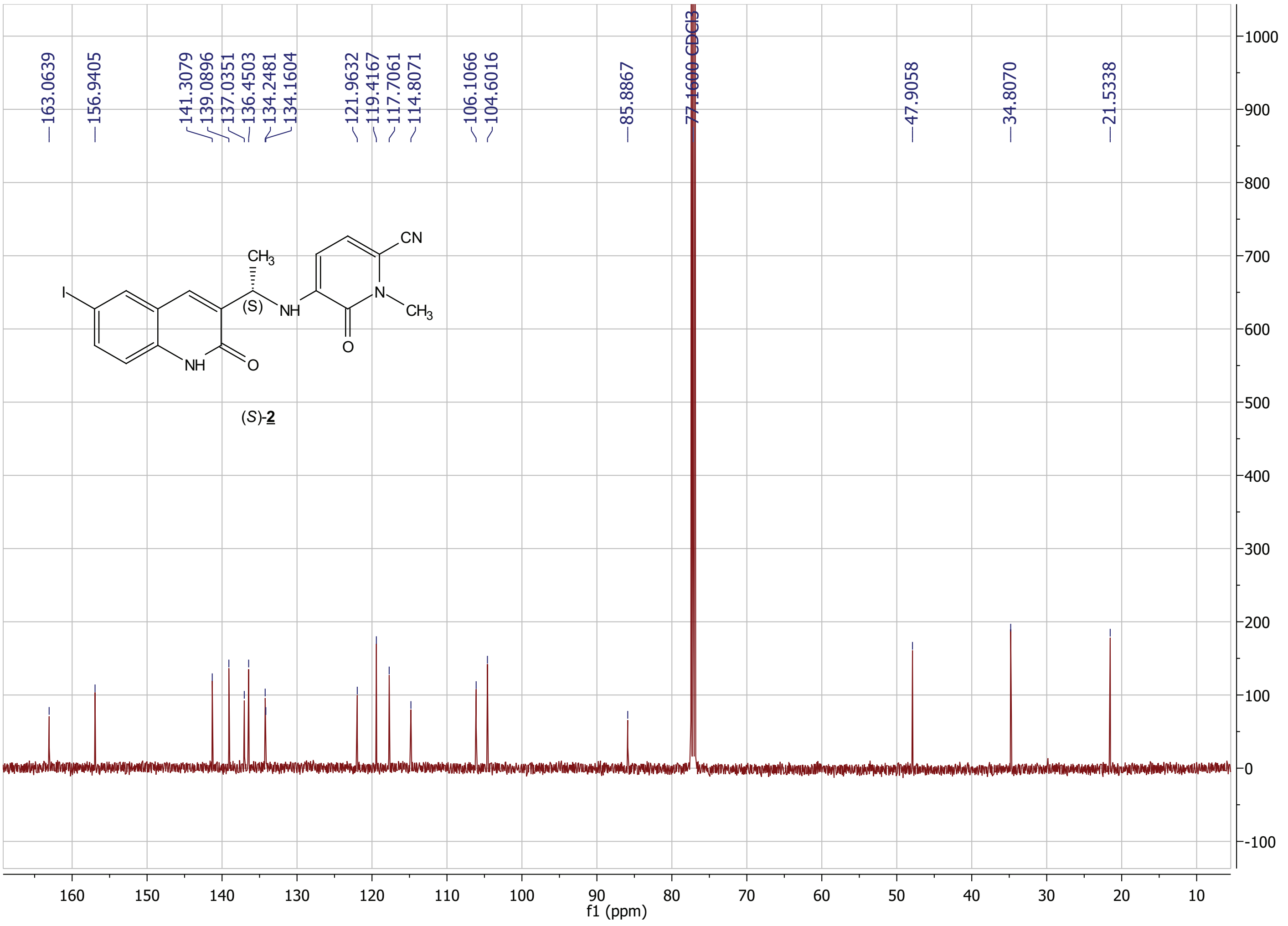
163.0639
156.9405
141.3079
139.0896
137.0351
136.4503
134.2481
134.1604
121.9632
119.4167
117.7061
114.8071
106.1066
104.6016
85.8867
77.1600 CDCl3
47.9058
34.8070
21.5338
CH3
CN
I
(S)
NH
N
CH3
NH
O
O
(S)-2
f1 (ppm)

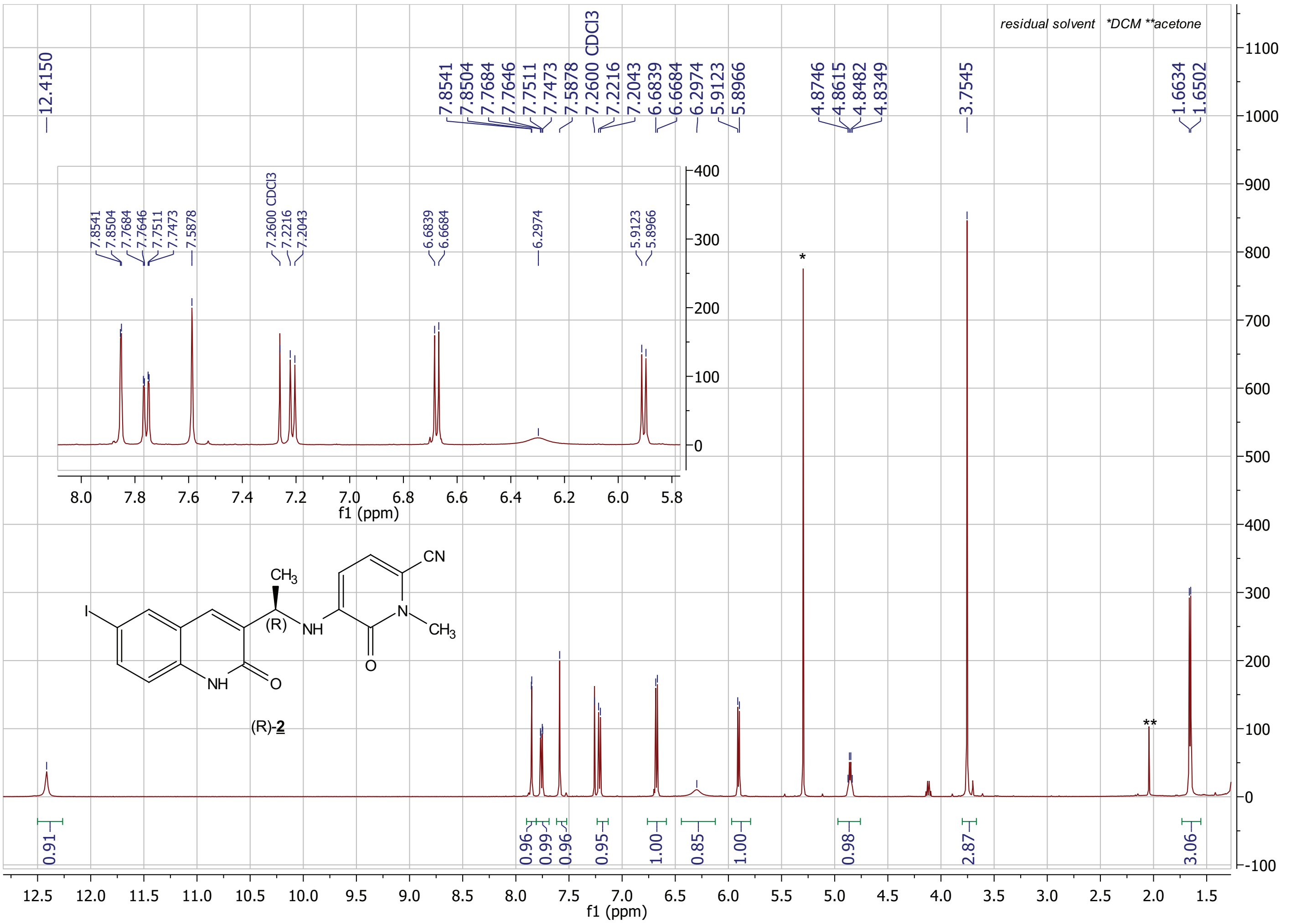

residual solvent *DCM **acetone
12.4150
7.8541
7.8504
7.7684
7.7646
7.7511
7.7473
7.5878
7.2600 CDCl3
7.2216
7.2043
6.6839
6.6684
6.2974
5.9123
5.8966
4.8746
4.8615
4.8482
4.8349
3.7545
1.6634
1.6502
CH3
CN
I
(R)
NH
N
CH3
O
NH
O
(R)-2
0.91
0.96
0.99
0.96
0.95
1.00
0.85
1.00
0.98
2.87
3.06
f1 (ppm)

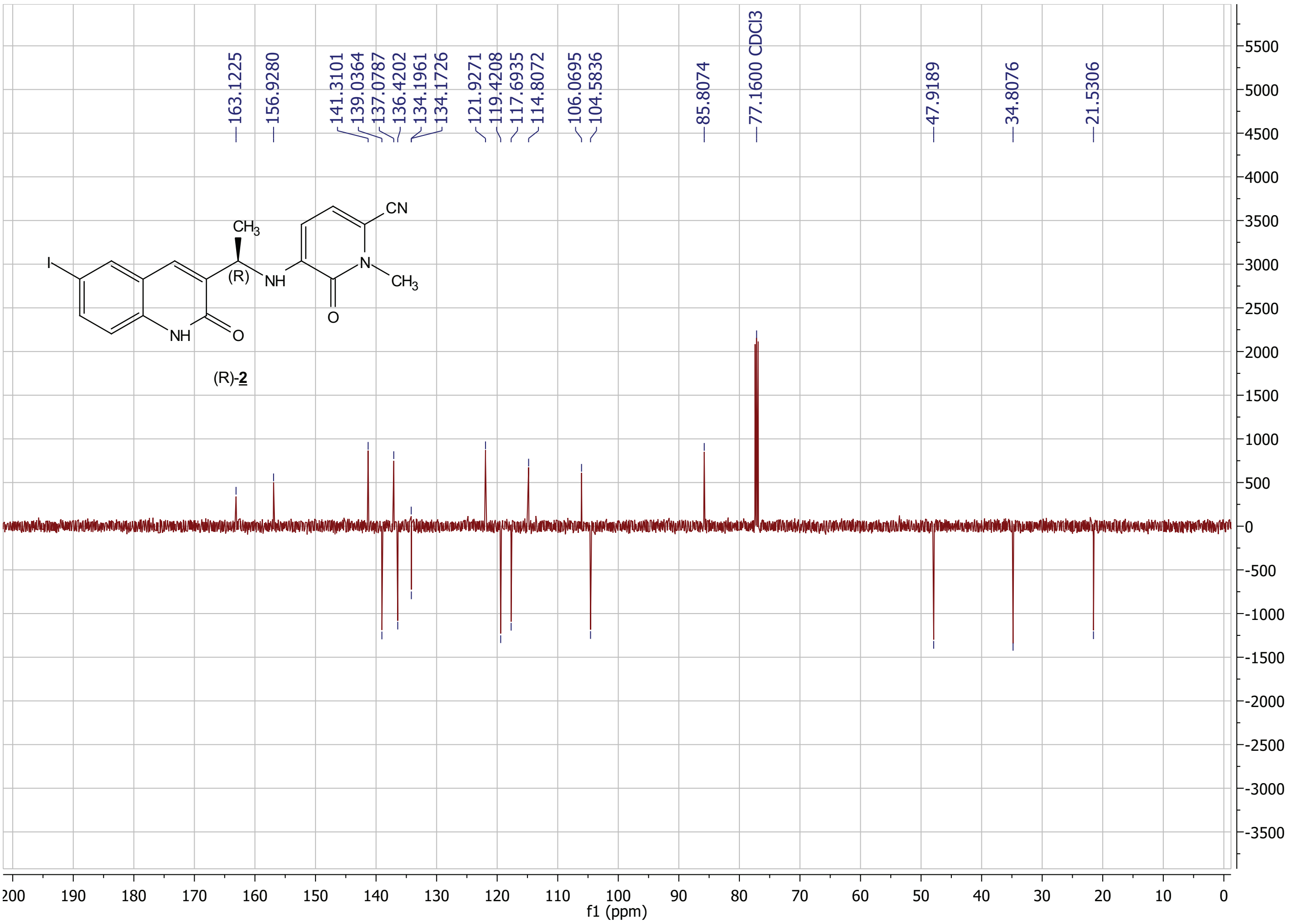

163.1225
156.9280
141.3101
139.0364
137.0787
136.4202
134.1961
134.1726
121.9271
119.4208
117.6935
114.8072
106.0695
104.5836
85.8074
77.1600 CDCl3
47.9189
34.8076
21.5306
CH3
CN
I
(R)
NH
N
CH3
O
NH
O
(R)-2
f1 (ppm)

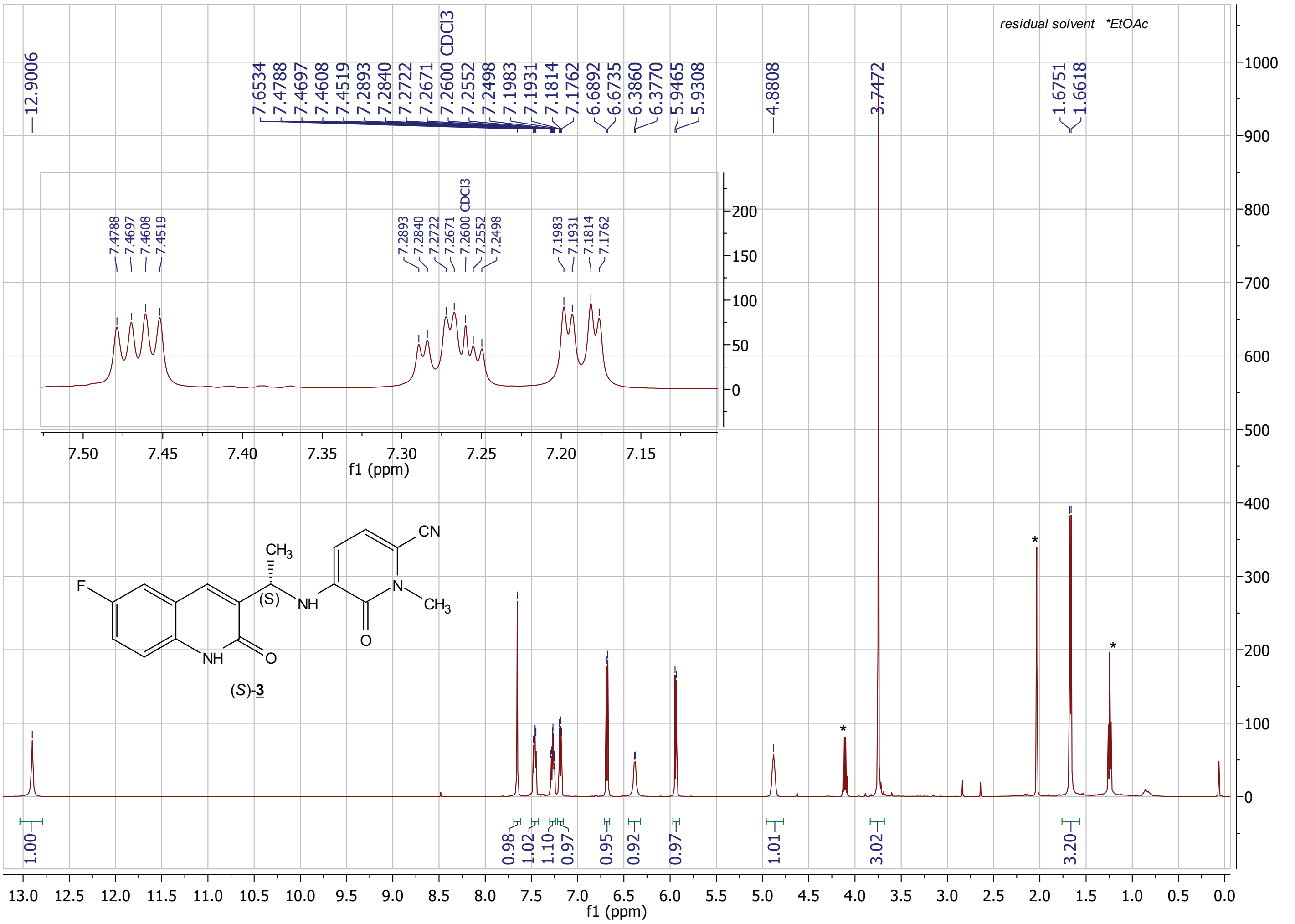
residual solvent *EtOAc
12.9006
7.6534
7.4788
7.4697
7.4608
7.4519
7.2893
7.2840
7.2722
7.2671
7.2600 CDCl3
7.2552
7.2498
7.1983
7.1931
7.1814
7.1762
6.6892
6.6735
6.3860
6.3770
5.9465
5.9308
4.8808
3.7472
1.6751
1.6618
1.00
0.98
1.02
1.10
0.97
0.95
0.92
0.97
1.01
3.02
3.20
f1 (ppm)
CH3
F
(S)
NH
CN
N
CH3
O
NH
O
(S)-3

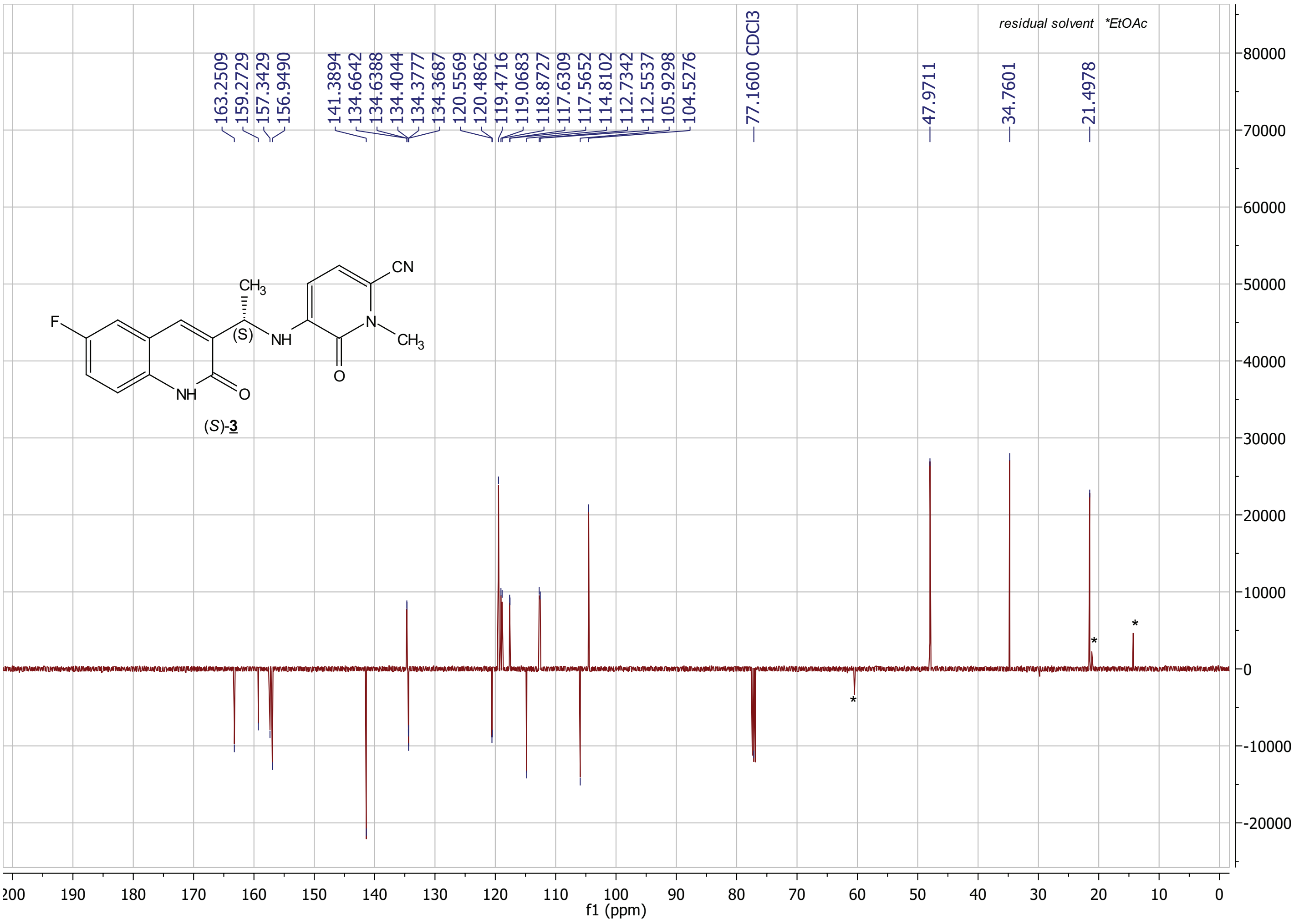
residual solvent *EtOAc
163.2509
159.2729
157.3429
156.9490
141.3894
134.6642
134.6388
134.4044
134.3777
134.3687
120.5569
120.4862
119.4716
119.0683
118.8727
117.6309
117.5652
114.8102
112.7342
112.5537
105.9298
104.5276
77.1600 CDCl3
47.9711
34.7601
21.4978
f1 (ppm)
CH3
F
(S)
NH
CN
N
CH3
O
NH
O
(S)-3

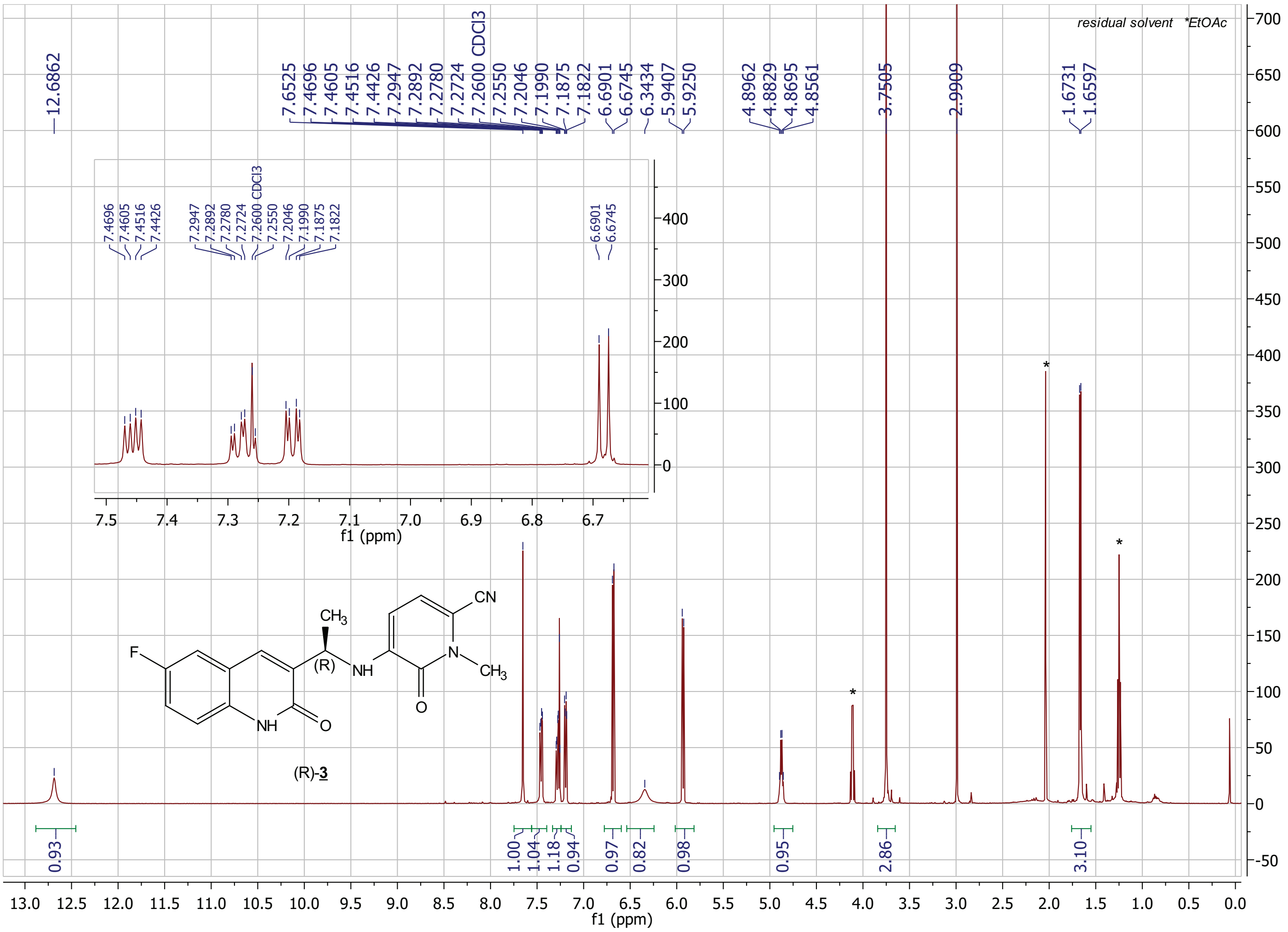
residual solvent *EtOAc
12.6862
7.6525
7.4696
7.4605
7.4516
7.4426
7.2947
7.2892
7.2780
7.2724
7.2600 CDCl3
7.2550
7.2046
7.1990
7.1875
7.1822
6.6901
6.6745
6.3434
5.9407
5.9250
4.8962
4.8829
4.8695
4.8561
3.7505
2.9909
1.6731
1.6597
f1 (ppm)
CH3
CN
F
(R)
NH
N
CH3
NH
O
O
(R)-3
0.93
1.00
1.04
1.18
0.94
0.97
0.82
0.98
0.95
2.86
3.10

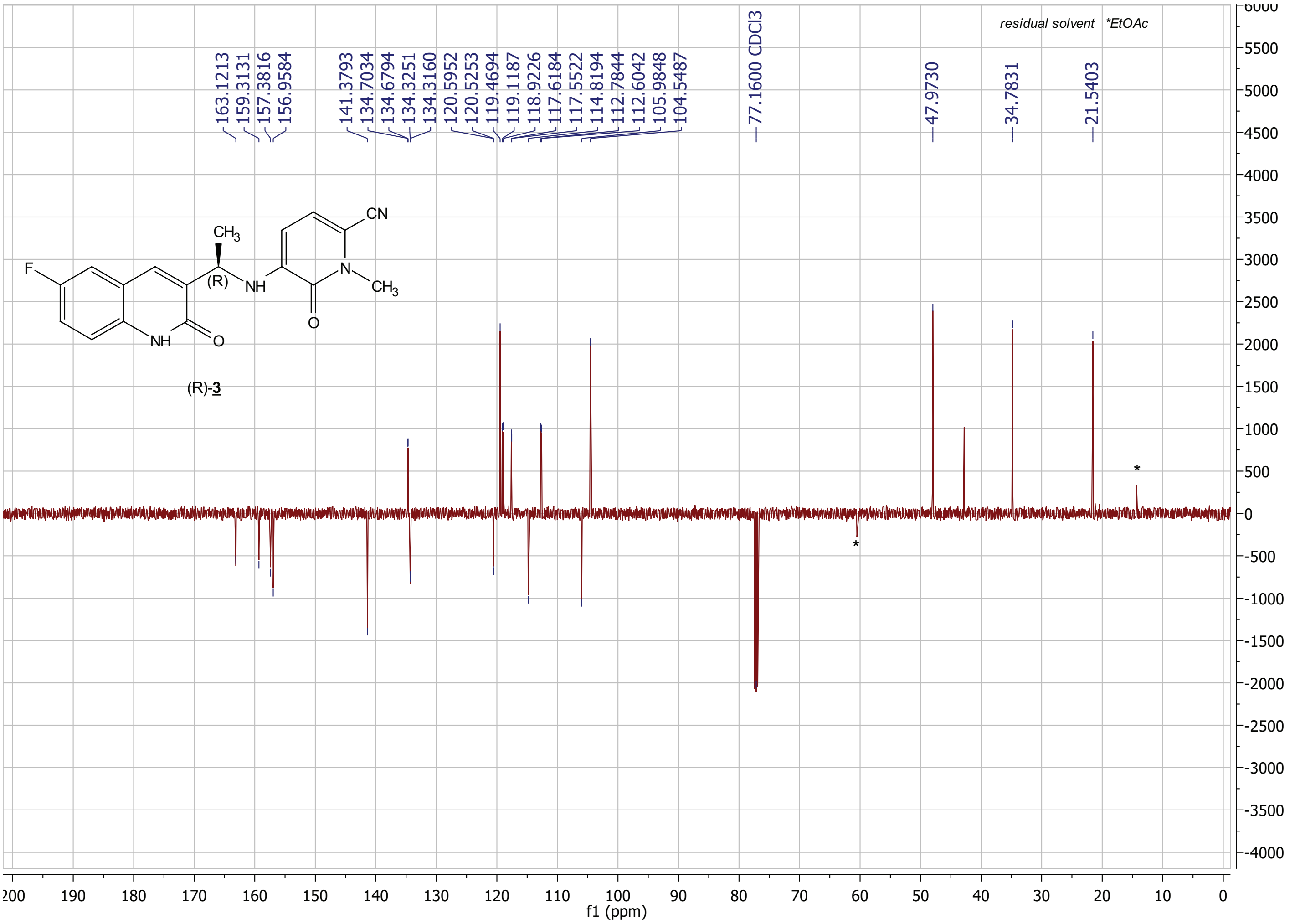
residual solvent *EtOAc
163.1213
159.3131
157.3816
156.9584
141.3793
134.7034
134.6794
134.3251
134.3160
120.5952
120.5253
119.4694
119.1187
118.9226
117.6184
117.5522
114.8194
112.7844
112.6042
105.9848
104.5487
77.1600 CDCl3
47.9730
34.7831
21.5403
f1 (ppm)
CH3
CN
F
(R)
NH
N
CH3
NH
O
O
(R)-3

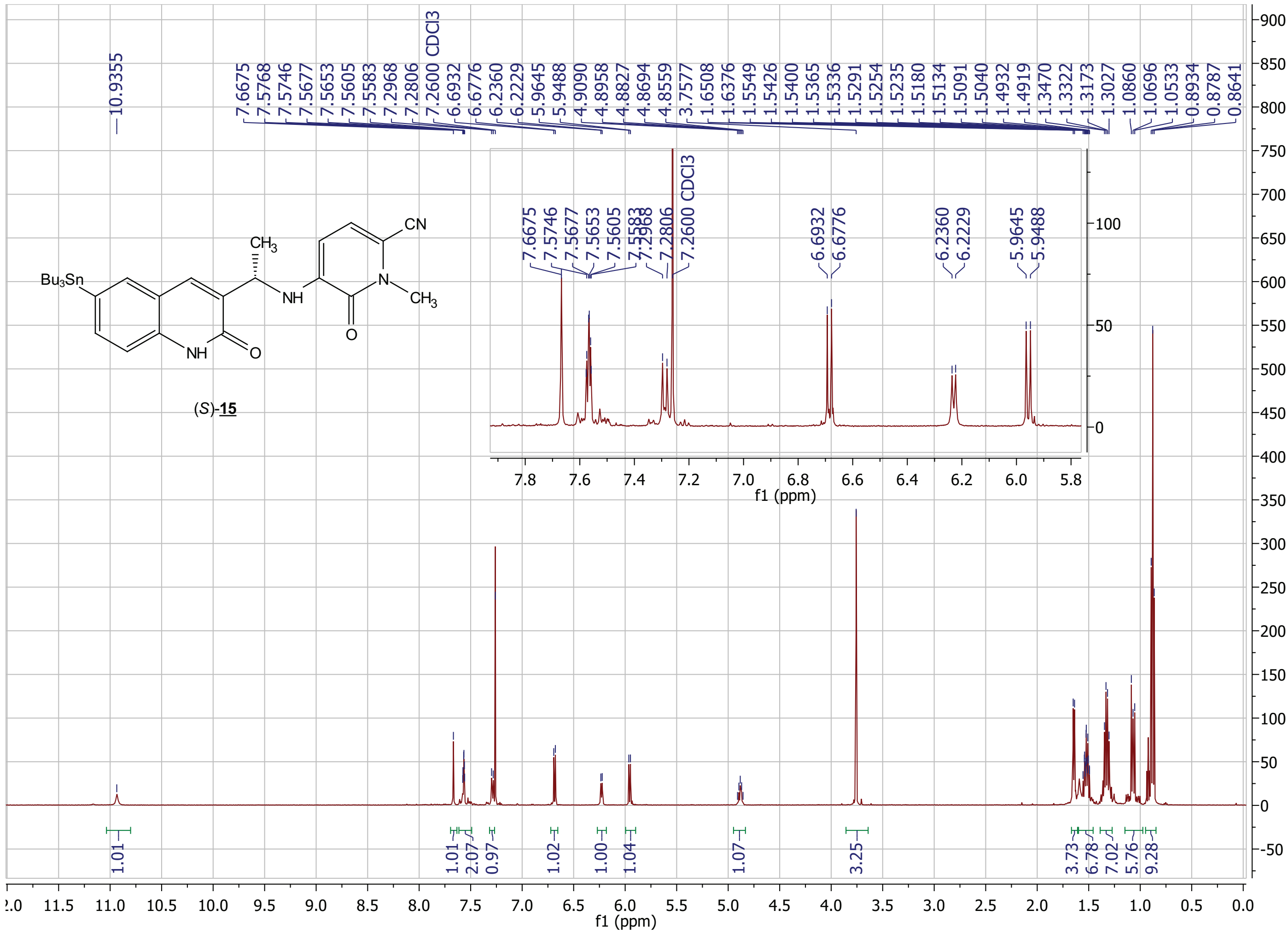
10.9355
7.6675
7.5768
7.5746
7.5677
7.5653
7.5605
7.5583
7.2968
7.2806
7.2600 CDCl3
6.6932
6.6776
6.2360
6.2229
5.9645
5.9488
4.9090
4.8958
4.8827
4.8694
4.8559
3.7577
1.6508
1.6376
1.5549
1.5426
1.5400
1.5365
1.5336
1.5291
1.5254
1.5235
1.5180
1.5134
1.5091
1.5040
1.4932
1.4919
1.3470
1.3322
1.3173
1.3027
1.0860
1.0696
1.0533
0.8934
0.8787
0.8641
Bu3Sn
CH3
CN
NH
N
CH3
O
NH
O
(S)-15
1.01
1.01
2.07
0.97
1.02
1.00
1.04
1.07
3.25
3.73
6.78
7.02
5.76
9.28
f1 (ppm)

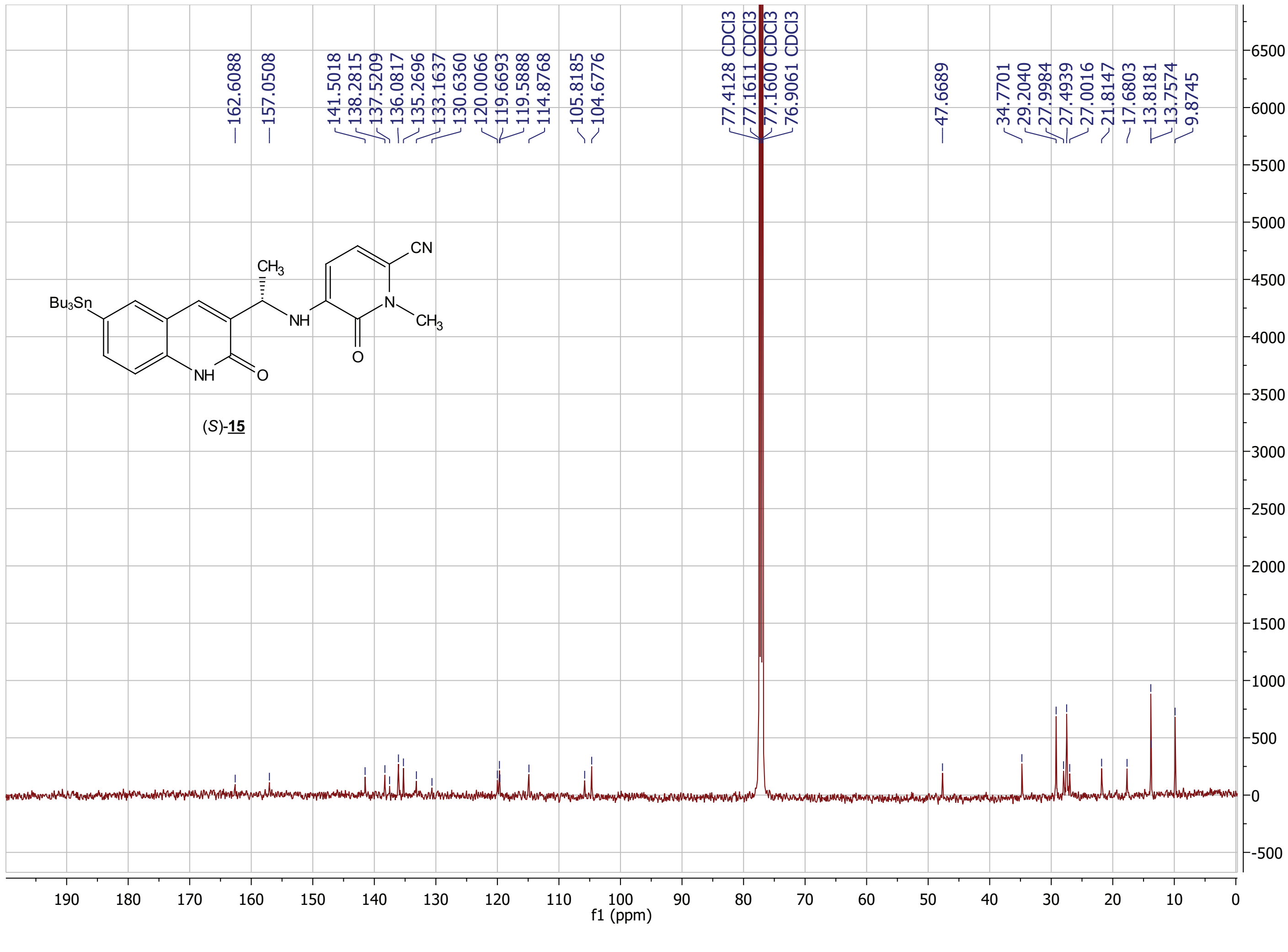
162.6088
157.0508
141.5018
138.2815
137.5209
136.0817
135.2696
133.1637
130.6360
120.0066
119.6693
119.5888
114.8768
105.8185
104.6776
77.4128 CDCl3
77.1611 CDCl3
77.1600 CDCl3
76.9061 CDCl3
47.6689
34.7701
29.2040
27.9984
27.4939
27.0016
21.8147
17.6803
13.8181
13.7574
9.8745
Bu3Sn
CH3
CN
NH
N
CH3
O
NH
O
(S)-15
f1 (ppm)
